# A sister of *NANOG* regulates genes expressed in pre-implantation human development

**DOI:** 10.1098/rsob.170027

**Published:** 2017-04-29

**Authors:** Thomas L. Dunwell, Peter W. H. Holland

**Affiliations:** Department of Zoology, University of Oxford, South Parks Road, Oxford OX1 3PS, UK

**Keywords:** NANOGNB, gene duplication, morula, homeobox, transcription factor

## Abstract

The *NANOG* homeobox gene plays a pivotal role in self-renewal and maintenance of pluripotency in human, mouse and other vertebrate embryonic stem cells, and in pluripotent cells of the blastocyst inner cell mass. There is a poorly studied and atypical homeobox locus close to the *Nanog* gene in some mammals which could conceivably be a cryptic paralogue of *NANOG,* even though the loci share only 20% homeodomain identity. Here we argue that this gene, *NANOGNB (NANOG Neighbour)*, is an extremely divergent duplicate of *NANOG* that underwent radical sequence change in the mammalian lineage. Like *NANOG*, the *NANOGNB* gene is expressed in pre-implantation embryos of human and cow; unlike *NANOG*, *NANOGNB* expression is restricted to 8-cell and morula stages, preceding blastocyst formation. When expressed ectopically in adult cells, human *NANOGNB* elicits gene expression changes, including downregulation of a set of genes that have an expression pulse at the 8-cell stage of pre-implantation development. We conclude that gene duplication and massive sequence divergence in mammals generated a novel homeobox gene that acquired new developmental roles complementary to those of *Nanog.*

## 1. Background

The *Nanog* gene was originally described independently by Wang *et al*. [1], Mitsui *et al*. [2] and Chambers *et al*. [3] and has been placed within the ANTP class of homeobox genes. *NANOG* is highly expressed in mouse and human embryonic stem cells (ESCs) and during pre-implantation stages of mbryo development from the 8-cell stage to the blastocyst, notably in the blastocyst inner cell mass which contributes to all somatic and germ-line tissues of the embryo. In mouse and human, the *Nanog* gene facilitates self-renewal of ESCs in culture and plays a central role in maintaining ESCs in a pluripotent state [2–4]. The gene is thought to have an analogous role in the embryo, being essential for maintenance of pluripotency by cells of the inner cell mass [2].

Although initially thought to be a singleton gene and not part of a gene family, some vertebrate species possess a second *Nanog* gene. Booth & Holland [5] reported that human *NANOG* has 11 pseudogenes, comprising 10 processed pseudogenes dispersed around the genome plus one duplication pseudogene (*NANOGP1*) generated by a segmental duplication involving *NANOG* and an adjacent solute carrier gene. *NANOGP1* was independently named *NANOG2* by Hart *et al*. [6] and this name was adopted as evidence accumulated that the locus was under selection and produced a protein [6–8]. The *NANOG2* locus is shared by humans and chimpanzees [7,9]. An independent duplication of *NANOG* was also reported in chicken [10], and more recently this duplication was found to be prevalent across birds. *Nanog* gene duplications have been noted in other species including guinea pig, coelacanth and gar [9]. In each case the paralogue (sister gene) to *Nanog* has a closely similar sequence to the progenitor gene.

We questioned whether this represents the totality of the *Nanog* gene family. A putative protein-coding locus 15 kb upstream of the human *NANOG* gene includes a highly divergent and anomalous homeobox sequence [11]. The locus was originally labelled LOC360030, then provisionally named homeobox c14, and renamed *NANOGNB* (*NANOG* *Neighbour*) in 2010 by the Human Gene Nomenclature Committee. The name *NANOGNB* was chosen to reflect chromosomal position with no inference made about evolutionary origin. Although the two loci are closely linked physically (in head to tail orientation), the deduced homeodomain of *NANOGNB* shares only 20% sequence identity with that of *NANOG* (12/60 amino acids): far below the normal extent of protein sequence similarity for duplicates within a homeobox gene family. Zhong & Holland [11] placed *NANOGNB* in a separate gene family to *NANOG*, along with three retroposed pseudogenes, and considered the homeodomain sequence to be so divergent that it could not even be placed in one of the 11 classes of bilaterian homeobox genes (by comparison, *NANOG* is clearly in the ANTP class [11,12]). In HomeoDB2, it was stated that if *NANOGNB* evolved by tandem gene duplication from *NANOG,* then it must have undergone ‘*massive sequence divergence’* [13]*.* There is no orthologue in mouse.

Scerbo *et al*. [9] included the *Nanognb* locus in an analysis of synteny around the *Nanog* gene in vertebrates. These authors noted *Nanognb* as present and adjacent to *Nanog* in human, chimpanzee, dog and elephant genomes. This is a far more restricted phylogenetic distribution than the *Nanog* gene itself, which is present in teleost fish, gar, coelacanth, anole lizard, turtle, birds and mammals [9]. Although secondarily lost from *Xenopus*, the *Nanog* gene is also present in urodele amphibians [14]. The *Nanog* gene, therefore, dates back at least to the base of osteichthyans, over 420 million years ago (Ma); in contrast, a recognizable *Nanognb* has only been reported from eutherian mammals, for which the crown-group common ancestor may be as recent as 61 Ma [15]. If *Nanognb* was indeed derived by duplication from *Nanog*, then the massive sequence divergence must have occurred in a relatively short period of time: just 20% homeodomain sequence identity now remains. By contrast, Hox genes such as *HOXA1* and *HOXB1*, separated by duplication over 450 Ma, share 88% homeodomain identity. Even the highly divergent *ARGFX* homeobox gene, dating to the base of eutherian mammals [16], shares 53% homeodomain identity with its progenitor *CRX*. The extremely low sequence similarity between *NANOG* and *NANOGNB* raises questions over whether the two neighbouring genes really share a recent common ancestry. However, it should also be noted that *NANOGNB* shares low sequence similarity with every other homeobox gene yet must have evolved from some progenitor; *NANOG* is perhaps the best candidate due to its physical proximity.

Here we investigate the origin and function of the *NANOGNB* homeobox gene. We show that *NANOGNB* is an extremely divergent duplicate (cryptic paralogue) of *NANOG* and that the human *NANOGNB* gene is expressed predominantly at the 8-cell to morula stages of development, initially concomitantly with *NANOG* but silenced earlier. Ectopic expression of *NANOGNB* in adult cells causes gene expression changes including downregulation of a set of genes which have a sharp expression peak at the 8-cell stage of pre-implantation development, presaging pluripotency. We suggest that gene duplication and massive divergence generated a novel homeobox gene that acquired new developmental roles in mammals complementary to those of *NANOG*.

## 2. Results

### 2.1. *NANOGNB* is a cryptic paralogue of *NANOG*

To examine if *NANOG* and *NANOGNB* are cryptic paralogues, with recent evolutionary history obscured by extensive amino acid sequence divergence, we deployed two strategies: identification of shared motifs and molecular phylogenetic analysis.

**Figure 1.**
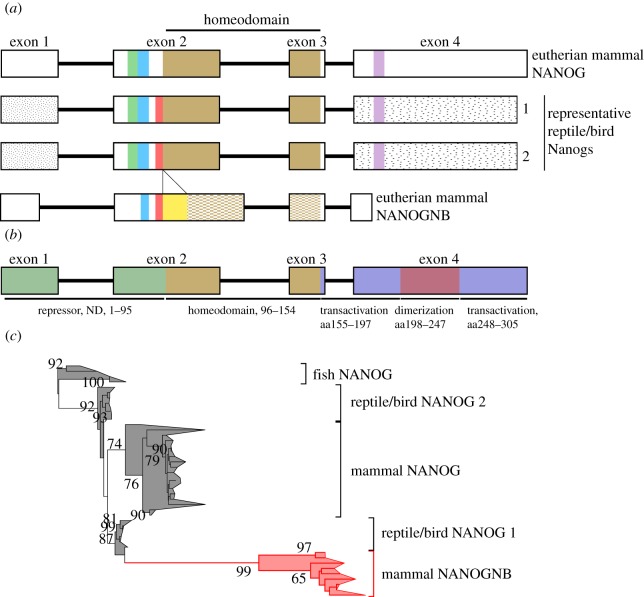
Identification of shared motifs and molecular phylogenetic analysis. (*a*) Schematic showing conserved motifs between NANOG and NANOGNB proteins from eutherians and reptiles/birds. The homeodomain is shaded in light brown; the hatched region in NANOGNB indicates sequence divergence. The blue region indicates a motif conserved between all NANOG and NANOGNB proteins; green and purple regions identify conservation between reptile and eutherian NANOG proteins; the red region represents conservation between reptile NANOG and eutherian NANOGNB; the yellow region represents a NANOGNB-specific insertion; speckled regions between reptile and bird NANOG indicate sequence conservation. (*b*) Known functional domains within mammalian NANOG. (*c*) Maximum-likelihood tree generated from a 147 amino acid alignable region spanning exons 2 and 3 of NANOG and NANOGNB genes, rooted using *Danio* and *Oryzias*. Support values above 65 are shown.

To search for shared motifs, we compared not only eutherian mammal *Nanog* and *Nanognb*, but also the single and duplicated *Nanog* genes of non-mammalian vertebrates (birds, reptiles and ray-finned fish). In total, 147 deduced protein sequences were included; full alignment data have been deposited [17]. Manual inspection revealed a mosaic of alignable and non-alignable sequences (figure 1*a*). Eutherian mammal Nanog proteins could be aligned well with each other, as could reptile/bird/fish Nanog proteins, but alignment between the two groups was limited outside the homeodomain despite their undoubted common ancestry. Short shared motifs were found both N-terminal and C-terminal to the homeodomain. Eutherian Nanognb proteins showed less similarity to either group, and the homeodomain sequence was markedly different. However, two small motifs N-terminal to the Nanognb homeodomain were identified as similar to motifs in Nanog proteins: the first motif (blue in figure 1*a*) is putatively shared with all Nanog proteins; the second (red in figure 1*a*) is identifiable in Nanog proteins of reptiles and birds, but not eutherian mammals. These motifs are located further from the homeodomain in Nanognb proteins due to a putative insertion. The presence of shared motifs is suggestive of common ancestry rather than definitive proof. We suggest that these motifs were present in a common ancestral vertebrate Nanog protein, and the mosaic pattern of conservation reflects differential retention in different genes and lineages. Comparison to the known functional domains of eutherian mammal NANOG suggests that NANOGNB does not contain the C-terminal transactivation and dimerization domains, while an N-terminal repressor domain is partially conserved (figure 1*b*).

For phylogenetic analysis, we first examined placement of *NANOGNB* within a phylogenetic tree of ANTP class homeodomains. If *NANOGNB* is a cryptic duplicate of *NANOG*, it would be expected to group with *NANOG* within the NKL subclass of ANTP genes. Phylogenetic analysis using all ANTP class homeodomains of chicken, human and zebrafish supported this prediction, with a distinct clustering of NANOG and NANOGNB sequences within the NKL subclass (electronic supplementary material, file S1: figure S1). However, this result is sensitive to sampling because when non-ANTP class homeodomains are included, the placement of NANOGNB is disrupted, perhaps due to long-branch effects (electronic supplementary material, file S1: figure S2). A phylogenetic analysis involving only NANOG and NANOGNB proteins permitted a longer sequence alignment to be used (exons 2 and 3) and is particularly revealing (figure 1*c*). When rooted using the single *Nanog* genes of teleost fish, the most logical root position, *NANOGNB* is placed nested within the amniote *NANOG* genes consistent with an origin from *NANOG*. A series of putative gene duplication events can be deduced. The two *Nanog* genes of reptiles and birds (denoted *Nanog1* and *Nanog2*) form deeply separated clades, although with *Pelodiscus* sequences are aberrantly placed; this suggests that an ancient gene duplication generated these genes prior to the radiation of extant reptiles and birds. Interestingly, the two genes are not grouped together to the exclusion of mammalian *Nanog*, suggesting that the duplication may be older than the reptile/mammal divergence. In this scenario, mammalian *Nanog* can only be orthologous to one of the reptile/bird genes, raising the question of what is the mammalian orthologue of the other reptile/bird *Nanog*? In our analysis, *Nanognb* falls as a sister group to the reptile/bird *Nanog1* gene, albeit on a long branch, and is suggested to be the missing orthologue of reptile/bird *Nanog1* (or conceivably of *Nanog2*), which underwent extensive sequence change specifically in eutherian mammals.

Hence, we propose that a single ancestral *Nanog* gene underwent tandem gene duplication before the divergence of extant reptiles, birds and mammals. After the origin of the mammalian lineage, one of these genes underwent radical sequence divergence to become *Nanognb*. Placement of sequences from the platypus (*Ornithorhychus*, a monotreme) suggests some of this sequence divergence occurred before divergence of monotremes and therian mammals, with divergence further pronounced in the eutherian mammals. Mouse has lost the *Nanognb* gene secondarily. Interestingly, *Nanognb* was not identified in marsupial mammals (*Sarcophilus*, *Monodelphis*), but two *Nanog* genes in *Sarcophilus* (Tasmanian devil) are both closer to *Nanog* in our phylogenetic analysis, possibly reflecting gene conversion or a separate gene duplication. Within fish, gar apparently has an independent duplication.

### 2.2. *NANOGNB* expression is more temporally restricted than *NANOG* expression

**Figure 2.**
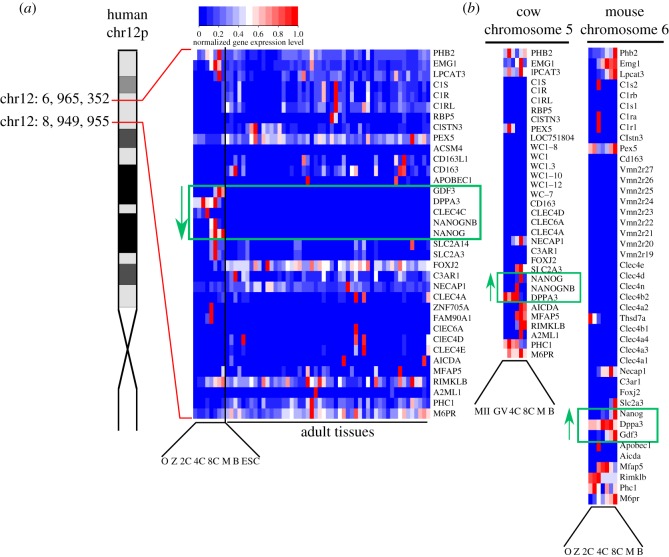
Expression analysis of genes in the chromosomal vicinity of NANOGNB in human and syntenic regions in mouse and cow. (*a*) Region surrounding *NANOGNB* on the short arm of human chromosome 12. Expanded view shows expression of 35 genes spanning a 2 Mb region (O, oocyte; Z, zygote; 2C, 2-cell embryo; 4C, 4-cell embryo; 8C, 8-cell embryo; M, morula; B, late blastocyst; ESC, embryonic stem cell). Adult tissues, left to right: adipose, adrenal gland, appendix, B-cell, bladder, bone marrow, brain: amygdala, brain: cerebellum, brain: cerebral cortex, brain: corpus callosum, brain: whole fetal, brain hippocampus, brain: parietal lobe, brain: substantis nigra, brain: whole, breast, CD34+ cells, CD4+ cells, CD8+ cells, colon, duodenum, endometrium, oesophagus, fallopian tube, gallbladder, heart, kidney, liver, lung, lymph node, macular retina, macular RPE, monocytes, natural killer cells, neutrophils, ovary, pancreas, placenta, prostate, salivary gland, skeletal muscle, skin, small intestine, smooth muscle, spleen, stomach, testes, thymus, thyroid, tonsil, whole blood. (*b*) Expression of genes in the syntenic regions in cow and mouse. MII and GV are oocyte stages. Expression values are normalized to the sample in each species where each gene is most highly expressed. Expression values below an FPKM of 2 are treated as unexpressed.

Genes closely linked to *NANOG* in human and mouse, notably *GDF3* and *DPPA3*, are similarly expressed in pluripotent embryonic stem cells and blastocysts [18], and evidence is accumulating for a chromatin domain with shared regulatory input around the *NANOG* gene extending at least as far as *GDF3* [19–21]. Since the human (and cow) *NANOGNB* gene lies within this region, between *NANOG* and *DPPA3*, we asked whether the gene shares a temporal expression profile with *NANOG*. Mapping of RNA-Seq reads for human and cow pre-implantation stages to a revised annotation of coding genes revealed that *NANOGNB* is expressed during pre-implantation development, but is limited to the 8-cell and morula stages (figure 2). In human it is clear that strong expression of the *NANOGNB* gene does not persist until blastocysts and is not detected in ESCs, unlike *NANOG*. Expression of *NANOGNB* therefore peaks and then drops earlier than *NANOG*.

Comparing temporal expression profiles to genomic order in the vicinity of *NANOG* in human, cow and mouse clearly demarcates a shared ‘pre-implantation gene expression’ domain, encompassing *NANOG*, *NANOGNB*, *CLEC4C*, *DPPA3* and *GDF3* (in human), *NANOG*, *NANOGNB* and *DPPA3* (in cow), or *Nanog*, *Dppa3* and *Gdf3* (in mouse). There is clear synteny between the chromosomal regions of these three species, albeit with inversions (figure 2; raw FPKM values in electronic supplementary material, file S2).

### 2.3. Targets down-regulated by *NANOGNB* are enriched for pre-implantation genes

Determining putative downstream targets of *NANOGNB* is complicated by the inaccessibility to experimentation of 8-cell and morula stages of human development, absence of expression in ESCs and lack of an orthologue in mice. In this situation, ablation of function by gene targeting is not a viable option for investigating function. Hence we used ectopic expression of V5-tagged human *NANOGNB* in primary adult human fibroblast cells to test if *NANOGNB* could elicit transcriptomic changes. We identified a total of 1070 upregulated and 1155 down-regulated genes with at least 1.3-fold expression change compared to cells transfected with empty vector control (electronic supplementary material, file S3).

To determine the biological significance of the transcriptome changes, we deployed a method to examine overlap with temporal profiles of gene expression in human development. This enables attention to be focused on those *NANOGNB*-responsive genes most likely to be direct or indirect targets of *NANOGNB* during pre-implantation development. Using RNA-Seq data from seven stages from oocyte to late blastocyst, we generated 69 distinct expression profiles (C1–C69). Of the 8837 human genes in these temporal profiles, 560 genes were upregulated and 584 down-regulated by ectopic *NANOGNB* in adult cells. These genes were not uniformly distributed between the 69 profiles (Pearson's *χ*^2^ test: UP *p* = 2.9 × 10^−7^, DOWN *p* = 1.0 × 10^−10^).

**Figure 3.**
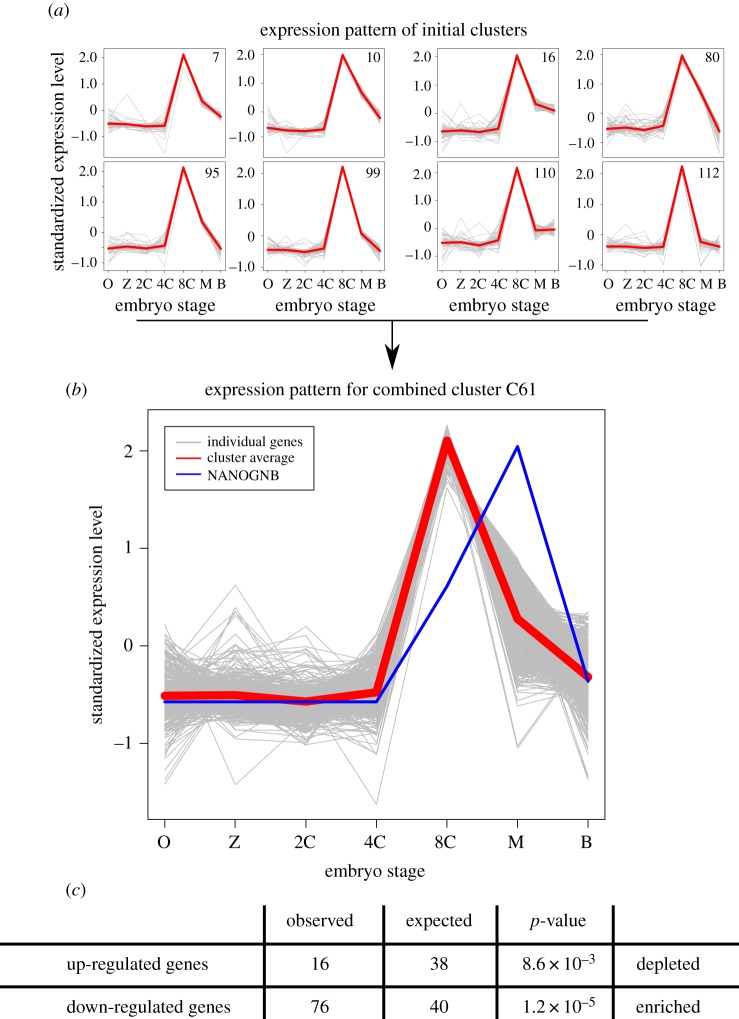
Enrichment of *NANOGNB*-responsive genes within gene expression profiles generated from embryo expression data. (*a*) Expression plots for individual genes and profile averages for the eight initial profiles which were combined to create combined profile C61. (*b*) Plots for all individual gene expression and profile average for combined profile C61. (*c*) Overlap of genes from the up- and downregulated genes after *NANOGNB* overexpression in fibroblast Fisher's exact test *p*-values. Grey lines represent standardized expression patterns for individual genes. Red lines represent the average standardized expression pattern for each profile. The blue line shows the relative standardized expression pattern for *NANOGNB* superimposed on profile C61. O, oocyte; Z, zygote; 2C, 2-cell embryo; 4C, 4-cell embryo; 8C, 8-cell embryo; M, morula; B, late blastocyst.

Three profiles showed significant enrichment for upregulated genes (profiles C17, C63, C67; combined Fisher's exact test *p* = 2.1 × 10^−6^), and three profiles were enriched for downregulated genes (profiles C19, C31, C61; combined Fisher's exact test *p* = 1.5 × 10^−11^) (electronic supplementary material, file S1: figure S3). The enrichment for downregulated genes in profile C61 is far larger than for any other profile, up- or downregulated (Fisher's exact test *p* = 6.7 × 10^−9^). The profile is also depleted for upregulated genes (Fisher's exact test *p* = 5.2 × 10^−5^). It is striking that this profile describes genes with a sharp peak in expression at the 8-cell stage in human development, decreasing rapidly in expression level precisely at the time when *NANOGNB* is increasing in expression (figure 3). This temporal correlation, together with the experimentally demonstrated transcriptomic effect, is consistent with a model in which *NANOGNB* represses the activity of genes showing a marked pulse of pre-morula and pre-blastocyst expression.

**Figure 4.**
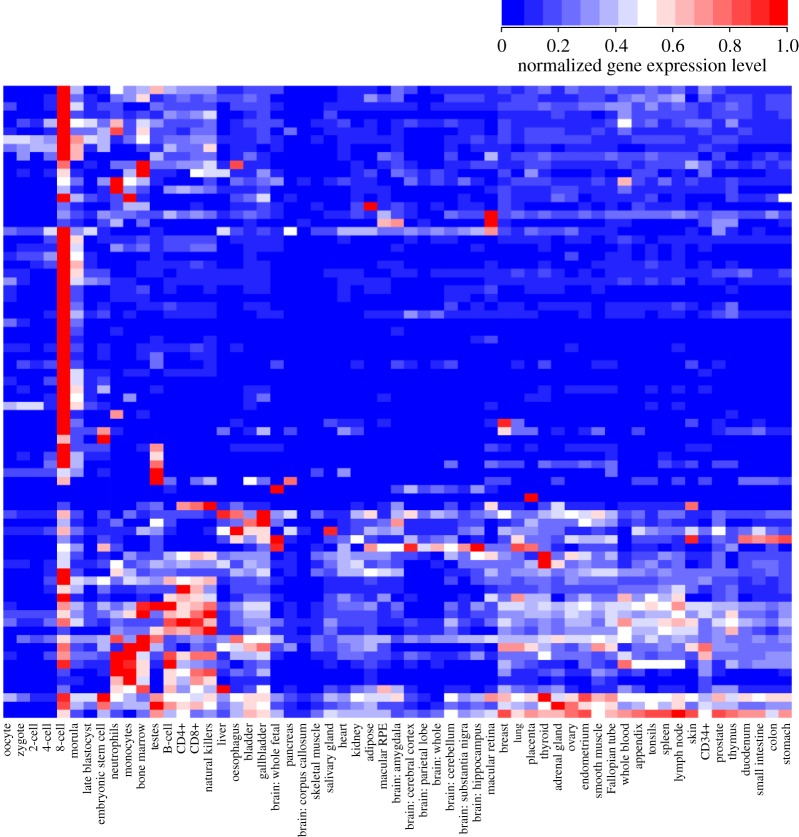
Heat map showing gene expression of the overlap genes between *NANOGNB* downregulated genes and expression profile C61. Expression values are normalized to the sample where each gene is highest expressed. Expression values below an FPKM of 2 are treated as unexpressed.

Temporal profile C61 contains 594 human genes, of which 76 are downregulated by ectopic *NANOGNB* in adult cells. To investigate the function of these 76 putative targets, we plotted their normal expression patterns in human early development and in adult tissues. An expected strong peak of expression at the 8-cell stage is clearly observed, but it is also evident that most of the putative targets are deployed again in adult tissues, with particular sets of genes expressed in testis and in the immune system (B cells, T cells, NK cells, monocytes, neutrophils; figure 4). The genes code for a wide range of proteins including a dual-specificity kinase CLK4 and several transcription factors (IRX1, HEY1 and RELB). The roles of the majority of the 76 genes in pre-implantation embryos are unknown.

## 3. Discussion

The *NANOGNB* gene has long been an enigma. First identified in human but absent in mouse, the gene was subsequently found in several other mammals. Its evolutionary origins were obscure because, although neighbouring the well-known *NANOG* gene, the homeodomain of *NANOGNB* is extremely divergent from any other homeodomain sequence known. Here we argue that *NANOGNB* was generated by tandem duplication of the *NANOG* gene followed by extensive, indeed dramatic, protein sequence divergence. Furthermore, we propose that the sequence divergence did not occur in all evolutionary lineages inheriting the duplicate genes, with birds/reptiles retaining two quite similar *Nanog* genes, while in mammals one paralogue diverged to become *Nanognb*.

Extensive sequence divergence after tandem duplication has been reported for other homeobox genes, although not as extreme as with *Nanognb*. For example, the human PRD class genes *ARGFX*, *DPRX*, *TPRX1*, *TPRX2* and *LEUTX* are divergent duplicates of the *CRX* gene, a member of the Otx gene family [16]. As with *NANOG* and *NANOGNB*, the ‘parental’ gene retained a sequence similar to that of the unduplicated condition, while the paralogues accumulated extensive sequence change. Such ‘asymmetric’ evolution of duplicated homeobox genes has also been reported in other taxa, such as duplicated Hox genes of Lepidoptera and TALE class genes of molluscs (reviewed by [22]).

It is interesting that *ARGFX*, *DPRX*, *TPRX* and *LEUTX* genes are, like *NANOGNB*, expressed in pre-implantation human embryos, and can regulate the expression of embryonic genes when ectopically expressed in adult cells and ESCs [16,23,24]. This may reveal a propensity of novel genes to be recruited to early developmental stages; for example, co-option to pathways regulating the formation of distinct cell lineages for embryo and placenta. This finding is also compatible with the phylotypic egg-timer model in which early and late stages of development are more amenable to evolutionary modification [25]. Alternatively, recruitment to early developmental stages may simply be a product of a paralogue inheriting or sharing *cis*-regulatory information with its progenitor gene. In this context, the *cis*-regulatory landscape around the *Nanog* gene is relevant.

It has long been recognized that three genes expressed in pluripotent human and mouse embryonic stem cells map as chromosomal neighbours: *Gdf3*, *Dppa3* and *Nanog* [18]. There is accumulating evidence for a degree of co-regulation of these genes. Mapping of local three-dimensional chromatin configuration around the human *NANOG* promoter using DNAse Hi-C revealed a single chromatin domain in human ESCs encompassing *GDF3*, *DPPA3* and *NANOG*, plus two other genes located between them: *NANOGNB* and *CLEC4C* [19]. Examination of chromosomal contacts using 3C in mouse ESCs has shown that the syntenic 160 kb domain folds into a physical loop from *Nanog* to *Gdf3* [20]. These authors also demonstrated that the looped region is enriched for binding sites for transcription factors involved in ESC self-renewal and that the genes are co-regulated. The latter study [20] and a recent functional dissection of the region by Blinka *et al*. [21] used mouse ESCs. In this species the *Nanognb* gene has been lost, leaving intergenic DNA between *Nanog* and *Dppa3*. If equivalent looping and co-regulation occurs in human ESCs, as suggested by DNase Hi-C [19], then it is expected that human *NANOGNB* would share some *cis*-regulatory input with *NANOG*.

Although *NANOGNB* and *NANOG* most probably share *cis*-regulatory input, their expression patterns are not identical. Specifically, expression of *NANOGNB* drops dramatically before the blastocyst is formed (FPKM value decreasing by over 90% from the morula level) and is undetectable in human ESCs. The peak of expression for *NANOGNB* is at the morula stage, and hence its functions must be different from those of *NANOG* which has a pivotal role in self-renewal of pluripotent cells in the inner cell mass of the blastocyst. Ectopic expression of *NANOGNB* reveals that the protein is capable of modulating gene expression, with a particularly strong effect being downregulation of genes that peak at the 8-cell stage of pre-implantation development. Hence, we conclude that the *in vivo* function of *NANOGNB* is to regulate a sharp pulse of gene expression that occurs at the 8-cell to morula stage, before cell fate commitment, comprising a suite of genes involved in transcription, intracellular signalling and cell division.

## 4. Methods

### 4.1. Embryo and adult tissue RNA-Seq profiling

Mapped and processed RNA-Seq expression data for normal human adult tissues and developmental stages and mouse developmental stages are from Dunwell & Holland [26], including a corrected gene model for human *NANOGNB*. RNA-Seq reads from cow embryonic stages were aligned to the bosTau8 reference genome using the STAR RNA-Seq aligner using the default settings with the addition of --outSAMstrandField intronMotif (electronic supplementary material, file S4; [27]). Expression levels for each sample were generated in the form of FPKM (fragments per kilobase per million reads) using Cufflinks [28].

### 4.2. Motif and phylogenetic analysis

Predicted peptide sequences were obtained from NCBI, Ensembl and HomeoDB2 [13], and are listed in the electronic supplementary material, file S5. Amino acid sequence alignments were generated using MAFFT v. 7.123b [29] and putatively homologous peptide motifs identified by eye; phylogenetic analysis was performed using RAxML v. 8.2.4 [30], after manual removal of poorly aligned regions, using settings: -T 30 -f a -k -x 12345 -p 12345 -d -# 500 -m PROTGAMMALG.

### 4.3. Ectopic *NANOGNB* gene expression

The human *NANOGNB* coding sequence was synthesized by GenScript USA and cloned in-frame with a C-terminal V5 tag under the control of a CMV promoter in a GFP co-expressing vector (pSF-CMV-Ub-daGFP AscI, Oxford Genetics #OG244). Electroporation into primary adult fibroblasts was performed as described [16]; cells were cultured for 48 h, harvested by trypsinization and resuspended in sorting buffer (2 mM EDTA, 25 mM HEPES, 0.5% BSA in Mg^2+^/Cl^2+^-free PBS). GFP-positive cells were enriched by sorting using a BD FACSARIA III, collecting 75 000–283 000 cells per replicate, and RNA was extracted using RNeasy Mini Kit (Qiagen). Three biological replicates were performed for *NANOGNB* and empty vector transfections. TruSeq (Illumina) libraries were prepared using 400 ng RNA per replicate at the Oxford Genomics Centre and sequenced using the Illumina HiSeq4000 platform generating between 42.8 and 61.3 million paired-end reads per replicate. Reads were aligned to the human reference genome GRCh38, raw read counts were generated with featureCounts and differential gene expression analysed in DESeq2 [31,32]. FPKM expression values for protein-coding genes were also generated using Cufflinks. Differentially expressed genes identified by DESeq2 were filtered by applying the following criteria: adjusted *p*-value ≤ 0.05 (Benjamini–Hochberg correction); upregulated to FPKM ≥ 2 or downregulated from FPKM ≥ 2; fold change ≥ 30% increase or decrease. Raw RNA-Seq reads and mapped featureCount data have been deposited to NCBI Gene Expression Omnibus (www.ncbi.nlm.nih.gov/geo) under accession GSE94053.

### 4.4. Embryo temporal profile enrichment analysis

To reveal which *NANOGNB*-responsive genes were probable *in vivo* targets, we examined overlap with temporal profiles of human gene expression, modified from the method described by Maeso *et al*. [16]. Gene expression values (FPKM) for seven human developmental time points (oocyte, zygote, 2-cell, 4-cell, 8-cell, morula and late blastocyst) were filtered to retain all expressed genes with a variance ≥ 5, resulting in a total of 8837 genes. The genes were then initially grouped into a total of 160 different expression profiles by Mfuzz based on expression changes across embryo stages, profile IDs 1–160 [33]. Clusters with similar temporal profiles, those with a pairwise Pearson correlation ≥ 0.95, were combined to generate a final collection of 69 distinct profiles, as designated collapsed profile IDs C1–C69.

A stepwise test was used to identify temporal profiles enriched or depleted for genes affected by *NANOGNB* ectopic expression. First, for each of the up- or downregulated genes, we identified in which of the 69 developmental profiles it was present; genes not present in any profile were removed. Second, a Pearson's *χ*^2^ test tested if *NANOGNB*-responsive genes were differentially assigned among profiles. As a statistically significant difference was seen (*p*-value ≤ 0.05), the Pearson's statistic for each profile was calculated to reveal which profiles contributed most to the difference. Third, to verify that identified profiles were responsible, a Pearson's *χ*^2^ test on the remaining profiles tested for significant difference among them. Fourth, temporal profiles found to be enriched or deleted in NANOGNB-responsive genes were combined and the significance of enrichment or depletion was determined using Fisher's exact test.

## Supplementary Material

Additional phylogenetic trees and profiles of NANOGNB-responsive genes

## Supplementary Material

Expression data for genes around NANOGNB

## Supplementary Material

Expression analysis of NANOGNB-responsive genes

## Supplementary Material

SRA datasets

## Supplementary Material

Amino acid sequences

## Data accessibility

Raw transcriptome data (sequencing reads) are deposited on SRA – GEO accession GSE94053 https://www.ncbi.nlm.nih.gov/geo/query/acc.cgi?token=elkvcusuxxibruz&acc=GSE94053. The datasets supporting this article have also been uploaded as part of the electronic supplementary material.
