# Supplementary material for: A sister of NANOG regulates genes expressed in pre-implantation human development
Source: Open Biol. 2017 Apr 29;7(4):170027. doi: 10.1098/rsob.170027 (PMC5413911; doi:10.1098/rsob.170027)
Supplement: Additional phylogenetic trees and profiles of NANOGNB-responsive genes [file rsob170027supp1.pdf]

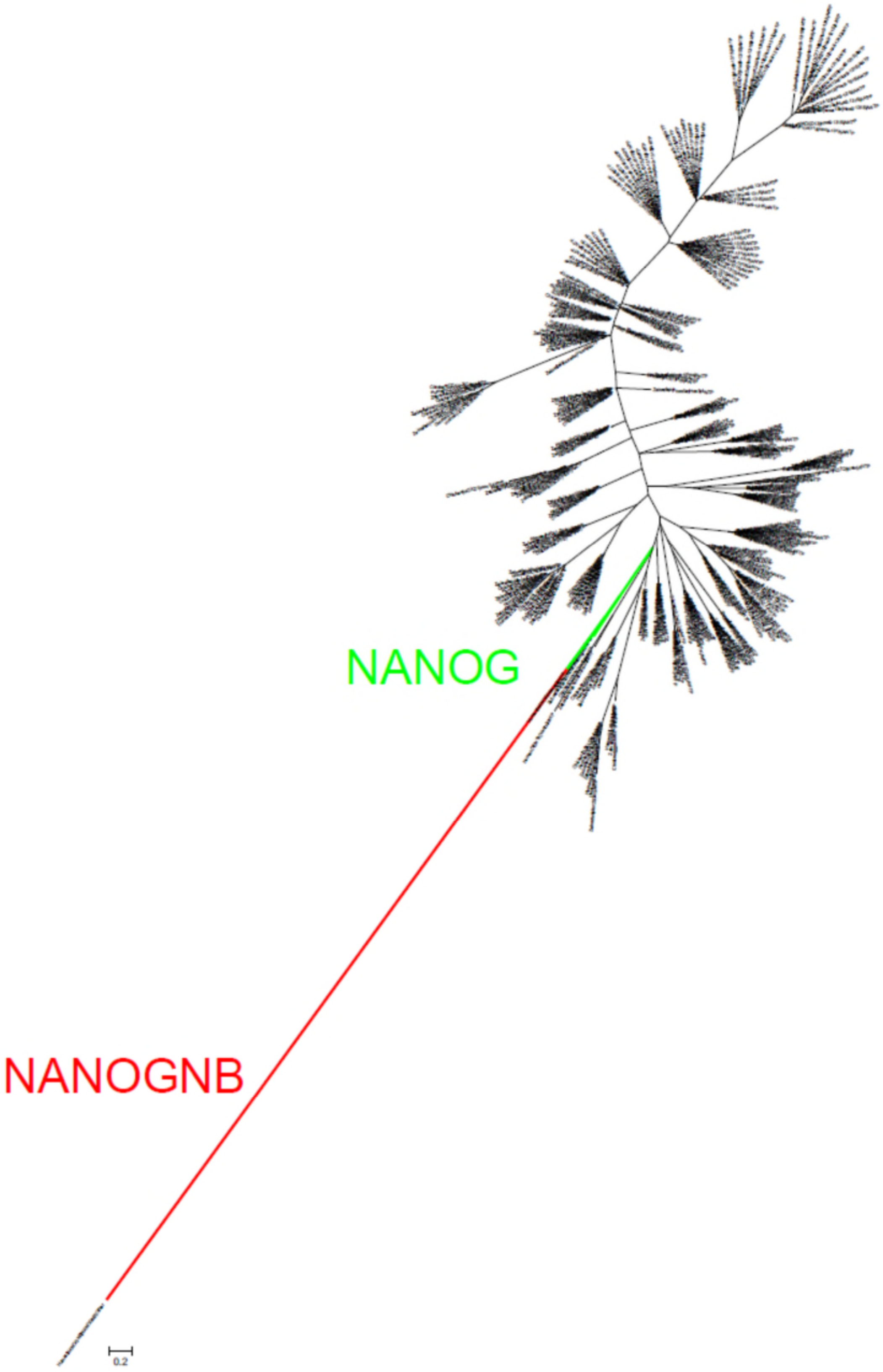

Supplementary Figure 1. Tree of all human, chicken, and zebrafish ANTP homeodomain sequences.

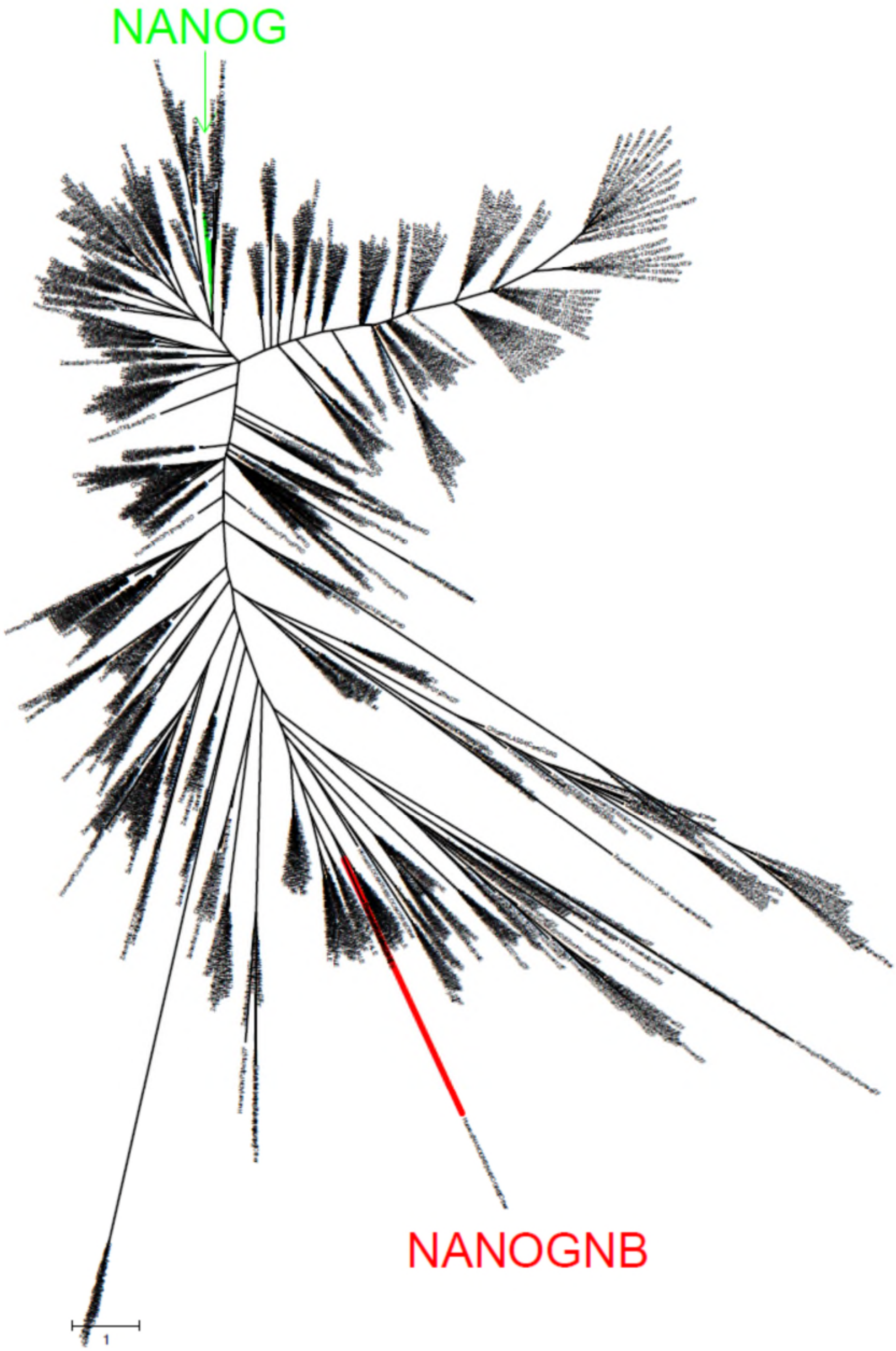

Supplementary Figure 2. Tree of all human, chicken, and zebra fish homeodomain sequences.

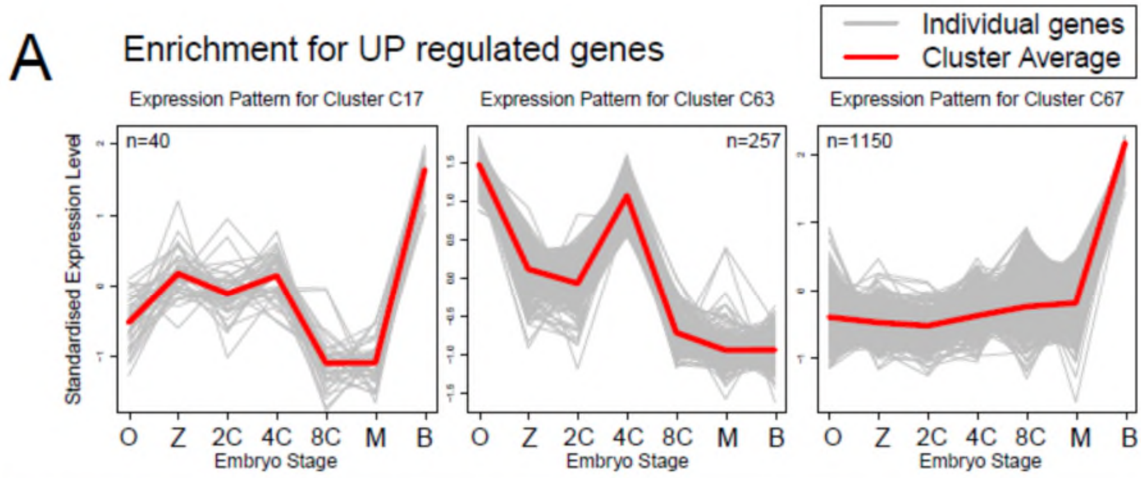

Number in cluster	7	30	97
p-Value	0.0128	0.0018	1.4×10^{-04}
Combined p-Value		2.081×10^{-6}	

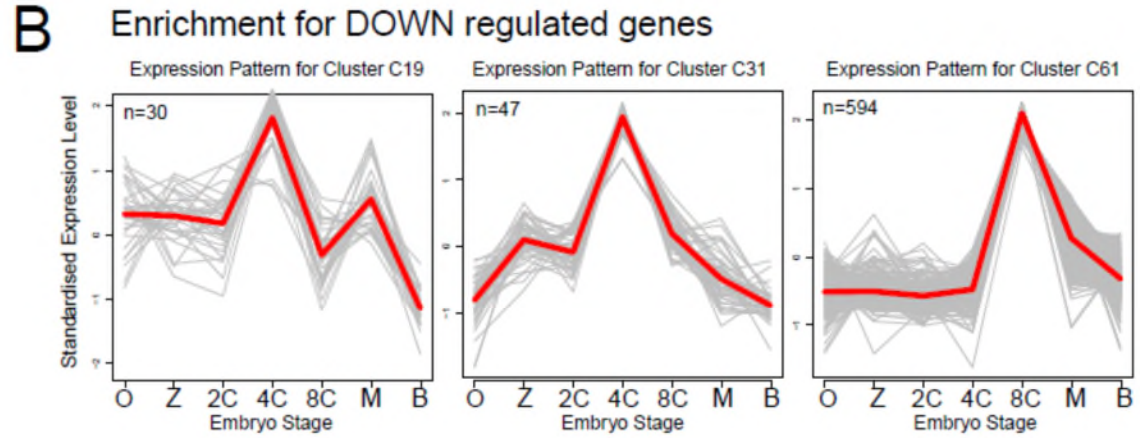

Number in cluster	6	9	76
p-Value	0.0115	0.0019	6.743×10^{-09}
Combined p-Value		1.466×10^{-11}	

Supplementary Figure 3. Expression profiles enriched for up- or down-regulated *NANOGNB*-responsive genes.
