# Supplementary material for: A sister of NANOG regulates genes expressed in pre-implantation human development
Source: Open Biol. 2017 Apr 29;7(4):170027. doi: 10.1098/rsob.170027 (PMC5413911; doi:10.1098/rsob.170027)
Supplement: Amino acid sequences [file rsob170027supp5.docx]

**ESM File 5: Amino acid sequences used in phylogenetic and domain analysis**

**Section A: Homeodomains from mouse, human and chicken used in phylogenetic analysis**

**Section B: NANOG and NANOGNB proteins used in phylogenetic and domain analysis**

**Section A:**

>Human|ADNP|Adnp|ZF

PKGHEDDSYEARKSFLTKYFNKQPYPTRREIEKLAASLWLWKSDIASHFSNKRKKCVRDC

>Chicken|ADNP|Adnp|ZF

PKGHEDDSYEARKTFLTKYFNKQPYPTRREIEKLAASLWLWKSDIASHFSNKRKKCVRDC

>Zebrafish|Adnp|Adnp|ZF

PKGYGDKSYEVRKAFLTAYFSRHPYPSQREVEKLAASLWLWKSDVASHFGNHRRLCDRD

>Zebrafish|adnp|Adnp|ZF

PKGYENESYETRKAFLTQYFNKQPYPTRREVEKLASSLWLWKSDIASHFSNRRRRCLLNC

>Human|ADNP2|Adnp|ZF

PKKYEGRSYEEKKQFLKDYFHKKPYPSKKEIELLSSLFWVWKIDVASFFGKRRYICMKAI

>Zebrafish|adnp2a|unassigned|Other

PMGMERTSFEDRKDFLSQYFHRKPYVTKTEIELLASRLWINKADVKAHFNSKLTKCLK

>Zebrafish|adnp2b|unassigned|Other

PAGMELRTFEDRREFVNKYFNTQPYPLKKEIIALSARLLLNKTDVACQISSKRTRCMKN

>Human|ALX1|Alx|PRD

KRRHRTTFTSLQLEELEKVFQKTHYPDVYVREQLALRTELTEARVQVWFQNRRAKWRKRE

>Chicken|ALX1|Alx|PRD

KRRHRTTFTSLQLEELEKVFQKTHYPDVYVREQLALRTELTEARVQVWFQNRRAKWRKRE

>Human|ALX3|Alx|PRD

KRRNRTTFSTFQLEELEKVFQKTHYPDVYAREQLALRTDLTEARVQVWFQNRRAKWRKRE

>Zebrafish|Alx3-l|Alx|PRD

KRRNRTTFSTFQLEELEKVFQKTHYPDVYAREQLALRTELTEARVQVWFQNRRAKWRKRE

>Human|ALX4|Alx|PRD

KRRNRTTFTSYQLEELEKVFQKTHYPDVYAREQLAMRTDLTEARVQVWFQNRRAKWRKRE

>Chicken|ALX4|Alx|PRD

KRRNRTTFTSYQLEELEKVFQKTHYPDVYAREQLAMRTDLTEARVQVWFQNRRAKWRKRE

>Zebrafish|alx4a|Alx|PRD

KRRNRTTFTSYQLEELEKVFQKTHYPDVYAREQLALRTDLTEARVQVWFQNRRAKWRKRE

>Zebrafish|alx4b|Alx|PRD

KRRNRTTFTSYQLEELEKVFQKTHYPDVYAREQLALRTDLTEARVQVWFQNRRAKWRKRE

>Human|ARGFX|Argfx|PRD

RHKERTSFTHQQYEELEALFSQTMFPDRNLQEKLALRLDLPESTVKVWFRNRRFKLKKQQ

>Human|ARX|Arx|PRD

QRRYRTTFTSYQLEELERAFQKTHYPDVFTREELAMRLDLTEARVQVWFQNRRAKWRKRE

>Chicken|ARX|Arx|PRD

QRRYRTTFSTFQLEELERAFRKSHYPDVFTREELALRLELTEARVQVWFQNRRAKWRKRE

>Zebrafish|arx|Arx|PRD

QRRYRTTFTSYQLEELERAFQKTHYPDVFTREELAMRLDLTEARVQVWFQNRRAKWRKRE

>Human|BARHL1|Barhl|ANTP

PRKARTAFTDHQLAQLERSFERQKYLSVQDRMELAASLNLTDTQVKTWYQNRRTKWKRQT

>Zebrafish|barhl1.1|Barhl|ANTP

PRKARTAFTDHQLAQLERSFERQKYLSVQDRMELAASLNLTDTQVKTWYQNRRTKWKRQT

>Zebrafish|barhl1.2|Barhl|ANTP

PRKARTAFTDHQLAQLERSFERQKYLSVQDRMELAASLNLTDTQVKTWYQNRRTKWKRQT

>Human|BARHL2|Barhl|ANTP

PRKARTAFSDHQLNQLERSFERQKYLSVQDRMDLAAALNLTDTQVKTWYQNRRTKWKRQT

>Zebrafish|barhl2|Barhl|ANTP

PRKARTAFSDHQLNQLERSFERQKYLSVQDRMDLAAALNLTDTQVKTWYQNRRTKWKRQT

>Human|BARX1|Barx|ANTP

GRRSRTVFTELQLMGLEKRFEKQKYLSTPDRIDLAESLGLSQLQVKTWYQNRRMKWKKIV

>Zebrafish|barx1|Barx|ANTP

GRRSRTVFTELQLMGLEKRFEKQKYLSTPDRIDLAESLGLSQLQVKTWYQNRRMKWKKIV

>Human|BARX2|Barx|ANTP

PRRSRTIFTELQLMGLEKKFQKQKYLSTPDRLDLAQSLGLTQLQVKTWYQNRRMKWKKMV

>Chicken|BARX2|Barx|ANTP

PRRSRTIFTELQLMGLEKKFQKQKYLSTPDRLDLAQSLGLTQLQVKTWYQNRRMKWKKMV

>Zebrafish|barx2|Barx|ANTP

PRRSRTIFTELQLLGLEKKFQKQKYLSTPDRLDLAQSLGLTQLQVKTWYQNRRMKWKKMV

>Zebrafish|bon|Mix|PRD

RRKRTNFTQQQIDVLEKVYLDTKYPDIYLREKLEALTGLPESRIQVWFQNRRAKSRRQ

>Zebrafish|brn1.2|Pou3|POU

KRKKRTSIEVSVKGALESHFLKCPKPGASEINSLADSLQLEKEVVRVWFCNRRQKEKRMT

>Human|BSX|Bsx|ANTP

RRKARTVFSDSQLSGLEKRFEIQRYLSTPERVELATALSLSETQVKTWFQNRRMKHKKQL

>Chicken|BSX|Bsx|ANTP

RRKARTVFSDSQLSGLEKRFEIQRYLSTPERVELATALSLSETQVKTWFQNRRMKHKKQL

>Zebrafish|bsx|Bsx|ANTP

RRKARTVFSDSQLSGLEKRFEIQRYLSTPERVELATALSLSETQVKTWFQNRRMKHKKQL

>Zebrafish|cart1|Alx|PRD

KRRHRTTFTSAQLEELEKVFQKTHYPDVYVREQLAMRTELTEARVQVWFQNRRAKWRKRE

>Human|CDX1|Cdx|ANTP

KDKYRVVYTDHQRLELEKEFHYSRYITIRRKSELAANLGLTERQVKIWFQNRRAKERKVN

>Chicken|CDX1|Cdx|ANTP

KDKYRVVYTDHQRLELEKEFHYSRYITIRRKAELAAALGLTERQVKIWFQNRRAKERKVN

>Zebrafish|cdx1a|Cdx|ANTP

KDKYRVVYSDVQRLELEKEFHFSRYITIRRKAELAGTLNLSERQVKIWFQNRRAKERKMN

>Zebrafish|cdx1b|Cdx|ANTP

KDKYRVVYTDHQRLELEKEFHYSRYITIRRKAELATALSLSERQVKIWFQNRRAKERKIN

>Human|CDX2|Cdx|ANTP

KDKYRVVYTDHQRLELEKEFHYSRYITIRRKAELAATLGLSERQVKIWFQNRRAKERKIN

>Chicken|CDX2|Cdx|ANTP

KDKYRVVYTDHQRLELEKEFHYSRYITIRRKAELASSLGLSERQVKIWFQNRRAKERKIN

>Human|CDX4|Cdx|ANTP

KEKYRVVYTDHQRLELEKEFHCNRYITIQRKSELAVNLGLSERQVKIWFQNRRAKERKMI

>Chicken|CDX4|Cdx|ANTP

REKYRVVYTDHQRLELEKEFHYNRYITIRRKSELAANLRLSERQVKIWFQNRRAKERKL

>Zebrafish|cdx4|Cdx|ANTP

KEKYRVVYTDHQRLELEKEFHFNRYITIRRKSELAVNLGLSERQVKIWFQNRRAKERKLI

>Chicken|CDX4-2|Cdx|ANTP

KEKYRVVYTDHQRLELEKEFHCNRYITIRRKSELAANLGLSERQVKIWFQN

>Chicken|Cers-l|Cers|CERS

QSPDGKRLEGLSKQLDWDVRKIQRWFRHRRNQDKP

>Zebrafish|Cers-l|Cers|CERS

RHRAEHNTVLEQHFTTKSKNPGQADIDGLCKKCGWSSRQVERWFRRRRNQDRP

>Zebrafish|Cers-l|Cers|CERS

IQANGPQKAQPNAILEKVFTAITKHPDEKRLEGLSKQLDWDVRTIQRWFRQRRNQEKPST

>Human|CERS2|Cers|CERS

NIKEKTRLRAPPNATLEHFYLTSGKQPKQVEVELLSRQSGLSGRQVERWFRRRRNQDRPSL

>Human|CERS3|Cers|CERS

GIKETVRKVTPNTVLENFFKHSTRQPLQTDIYGLAKKCNLTERQVERWFRSRRNQERPSR

>Human|CERS4|Cers|CERS

GVRDQTRRQVKPNATLEKHFLTEGHRPKEPQLSLLAAQCGLTLQQTQRWFRRRRNQDRPQL

>Human|CERS5|Cers|CERS

GIEDSGPYQAQPNAILEKVFISITKYPDKKRLEGLSKQLDWNVRKIQCWFRHRRNQDKPPT

>Human|CERS6|Cers|CERS

NIQANGPQIAPPNAILEKVFTAITKHPDEKRLEGLSKQLDWDVRSIQRWFRQRRNQEKPST

>Zebrafish|chr04.1|Alx|PRD

RRIRTTFTVTQLQELERVFQDTHYPDVQTRDLLACRTQLTEARVQVWFQNRRAKWRR

>Zebrafish|chr05.1|Gsc|PRD

RRHRTIFTEEQLQALEDLFTHNQYPDIHTREQLALKTQLREERVEVWFKNRRAKWRRQK

>Chicken|chr10.1|Onecut|CUT

KPRLVFTDVQRRTLHAIFKENKRPSKELQITISQQLGLELSTVSNFFMNARR

>Zebrafish|chr19_01|unassigned|Other

EQQMKlLEKSFQKNSCPSYSEVESLQISSRLSREDVESWFVGRRA

>Zebrafish|chr19_02|unassigned|Other

LISAFSRFPYPTPAELTGLTAASTHPEQQIRLWFSTQRLKQGI

>Chicken|chrUn.1|unassigned|Other

RTKFSAVQLQELERSFREQRYIGASEKRRLAAALDLSQSQIKTWFQNRRMKFKRE

>Chicken|chrZ.1|Onecut|CUT

KSRLVFTDLQRRTLFAIFKENKRPSKEMQITISQQLGLELTTVSNFFMNARRR

>Chicken|CNOT2|Noto|ANTP

KRVRTIFTSDQLARLEKEFARQQYMVGTERCLLASALHLTEEQVKVWFQNRRIKWRK

>Human|CPHX1|Cphx|Other

KTKHRHKFSEELLQELKEIFGENCYPDYTTRKTLAIKFDCPVNVIDNWFQNKRARLPPAE

>Human|CPHX2|Cphx|Other

KTKHRHKFSEELLQELKEIFGENGYPDFTTRKTLANKFDCPVNVINNWFQNNRARLPPEE

>Human|CRX|Otx|PRD

QRRERTTFTRSQLEELEALFAKTQYPDVYAREEVALKINLPESRVQVWFKNRRAKCRQQR

>Zebrafish|crx|Otx|PRD

QRRERTTFTRTQLDILEALFTKTRYPDIFMREEVALKINLPESRVQVWFKNRRAKCRQQQ

>Human|CUX1|Cux|CUT

LKKPRVVLAPEEKEALKRAYQQKPYPSPKTIEDLATQLNLKTSTVINWFHNYRSRIRREL

>Chicken|CUX1|Cux|CUT

LKKPRVVLAPEEKEALKRAYQQKPYPSPKTIEELATQLNLKTSTVINWFHNYRSRIRREL

>Human|CUX2|Cux|CUT

IKKPRVVLAPEEKEALRKAYQLEPYPSQQTIELLSFQLNLKTNTVINWFHNYRSRMRREM

>Chicken|CUX2|Cux|CUT

IKKPRVVLAPEEKEALKKAYQLEPYPSQQTIELLSFQLNLKTNTVINWFHNYR

>Zebrafish|Cux2-l|Cux|CUT

KKPRVVLAAEEKEALRKAYLQEPYPSQHTIEMLAAQLNLKTNTVINWFHNYRSRMRREV

>Human|DBX1|Dbx|ANTP

GMLRRAVFSDVQRKALEKMFQKQKYISKPDRKKLAAKLGLKDSQVKIWFQNRRMKWRNSK

>Chicken|DBX1|Dbx|ANTP

GMLRRAVFSDVQRKALEKMFQKQKYISKPDRKKLAAKLGLKDSQVKIWFQNRRMKWRNSK

>Zebrafish|dbx1a|Dbx|ANTP

GMLRRAVFSDVQRKALEKMFQKQKYISKPDRKKLAAKLGLKDSQVKIWFQNRRMKWRNSK

>Zebrafish|dbx1b|Dbx|ANTP

GMLRRAVFSDVQRKALEKMFQKQKYISKPDRKKLATKLGLKDSQVKIWFQNRRMKWRNSK

>Human|DBX2|Dbx|ANTP

GILRRAVFSEDQRKALEKMFQKQKYISKTDRKKLAINLGLKESQVKIWFQNRRMKWRNSK

>Chicken|DBX2|Dbx|ANTP

GILRRAVFSEDQRKALEKMFQKQKYISKTDRKKLAINLGLKESQVKIWFQNRRMKWRNSK

>Zebrafish|dbx2|Dbx|ANTP

GILRRAVFSEEQRRELEKTFSKQKYISKTERNRLASELSLKETQVKIWFQNRRMKWRNSR

>Zebrafish|dharma|unassigned|Other

RIRTVFTDNQTEQLERLFAVTDYPTVETRAELAQNTGLSEETVRVWFKNRRARRKRQ

>Human|DLX1|Dlx|ANTP

IRKPRTIYSSLQLQALNRRFQQTQYLALPERAELAASLGLTQTQVKIWFQNKRSKFKKLM

>Chicken|DLX1|Dlx|ANTP

IRKPRTIYSSLQLQALNRRFQQTQYLALPERAELAASLGLTQTQVKIWFQNKRSKFKKLM

>Zebrafish|dlx1a|Dlx|ANTP

IRKPRTIYSSLQLQALNRRFQQTQYLALPERAELAASLGLTQTQVKIWFQNKRSKFKKLM

>Human|DLX2|Dlx|ANTP

VRKPRTIYSSFQLAALQRRFQKTQYLALPERAELAASLGLTQTQVKIWFQNRRSKFKKMW

>Zebrafish|dlx2a|Dlx|ANTP

VRKPRTIYSTFQLAALQRRFQKTQYLALPERAELAASLGLTQTQVKIWFQNRRSKFKKLW

>Zebrafish|dlx2b|Dlx|ANTP

VRKPRTIYSSFQLAALQRRFQKTQYLALPERAELAASLGLTQTQVKIWFQNRRSKFKKLW

>Human|DLX3|Dlx|ANTP

VRKPRTIYSSYQLAALQRRFQKAQYLALPERAELAAQLGLTQTQVKIWFQNRRSKFKKLY

>Chicken|Dlx3-l|Dlx|ANTP

RKPRTIYSSYQLAALQRRFQKAQYLALPERAELAAQLGLTQTQV

>Zebrafish|dlx3b|Dlx|ANTP

IRKPRTIYSSYQLAALQRRFQKAQYLALPERAELAAQLGLTQTQVKIWFQNRRSKFKKLY

>Human|DLX4|Dlx|ANTP

LRKPRTIYSSLQLQHLNQRFQHTQYLALPERAQLAAQLGLTQTQVKIWFQNKRSKYKKLL

>Zebrafish|dlx4a|Dlx|ANTP

IRKPRTIYSSLQLQALNQRFQQTQYLALPERADLAAKLGLTQTQVKIWFQNKRSKYKKIM

>Zebrafish|dlx4b|Dlx|ANTP

IRKPRTIYSSVQLQALHQRFQQTQYLALPERADLAAKLGLTQTQVKIWFQNKRSKYKKIM

>Human|DLX5|Dlx|ANTP

VRKPRTIYSSFQLAALQRRFQKTQYLALPERAELAASLGLTQTQVKIWFQNKRSKIKKIM

>Chicken|DLX5|Dlx|ANTP

VRKPRTIYSSFQLAALQRRFQKTQYLALPERAELAASLGLTQTQVKIWFQNKRSKIKKIM

>Zebrafish|dlx5a|Dlx|ANTP

VRKPRTIYSSFQLAALQRRFQNTQYLALPERAELAASLGLTQTQVKIWFQNKRSKLKKIM

>Human|DLX6|Dlx|ANTP

IRKPRTIYSSLQLQALNHRFQQTQYLALPERAELAASLGLTQTQVKIWFQNKRSKFKKLL

>Chicken|DLX6|Dlx|ANTP

IRKPRTIYSSLQLQALNHRFQQTQYLALPERAELAASLGLTQTQVKIWFQNKRSKFKKLL

>Zebrafish|dlx6a|Dlx|ANTP

IRKPRTIYSSLQLQALNHRFQQTQYLALPERAELAASLGLTQTQVKIWFQNKRSKFKKLL

>Human|DMBX1|Dmbx|PRD

QRRSRTAFTAQQLEALEKTFQKTHYPDVVMRERLAMCTNLPEARVQVWFKNRRAKFRKKQ

>Chicken|DMBX1|Dmbx|PRD

QRRSRTAFTAQQLEALEKTFQKTHYPDVVMRERLAMCTNLPEARVQVWFKNRRAKFRKKQ

>Zebrafish|Dmbx1-l|Dmbx|PRD

QRRSRTAFSVSQLQALEKAFQQTQYPDVGMRERLAVCINLPEARIQVWFKNRRAKFRKGQ

>Zebrafish|dmbx1a|Dmbx|PRD

QRRSRTAFTAQQLEALEKTFQKTHYPDVVMRERLAMCTNLPEARVQVWFKNRRAKFRKKQ

>Zebrafish|dmbx1b|Dmbx|PRD

QRRSRTAFTAQQLEALEKTFQKTHYPDVVMRERLAMCTNLPEARVQVWFKNRRAKFRKKQ

>Human|DPRX|Dprx|PRD

SHRKRTMFTKKQLEDLNILFNENPYPNPSLQKEMASKIDIHPTVLQVWFKNHRAKLKKAK

>Human|DRGX|Drgx|PRD

QRRNRTTFTLQQLEALEAVFAQTHYPDVFTREELAMKINLTEARVQVWFQNRRAKWRKTE

>Chicken|DRGX|Drgx|PRD

QRRNRTTFTLQQLEALEAVFAQTHYPDVFTREELAMKINLTEARVQVWFQNRRAKWRKTE

>Zebrafish|drgx|Drgx|PRD

QRRNRTTFTLQQLEALEAVFAQTHYPDVFAREELAMKINLTEARVQVWFQNRRAKWRKTE

>Human|DUX4|HD1|Dux|PRD

GRRRRLVWTPSQSEALRACFERNPYPGIATRERLAQAIGIPEPRVQIWFQNERSRQLRQH

>Human|DUX4|HD2|Dux|PRD

GRRKRTAVTGSQTALLLRAFEKDRFPGIAAREELARETGLPESRIQIWFQNRRARHPGQG

>Human|Dux4l|HD1|Dux|PRD

QQRIVLNSSQKDALQALFKQNSYPGIPTREQLAKEIGSLESRIQIWFQNQ

>Human|Dux4l|HD2|Dux|PRD

AITKPQTGVLLQAFQRNRFPGTAIKEELAKQTGIPESRI

>Human|DUX4L10|HD1|Dux|PRD

GRRRRLVWTPSQSEALRACFERNPYPGIATRERLAQAIGIPEPRVQIWFQNERSRQLRQH

>Human|DUX4L10|HD2|Dux|PRD

GRRKRTAVTGSQTALLLRAFEKDRFPGIAAREELARETGLPESRIQIWFQNRRARHPGQG

>Human|DUX4L11|HD1|Dux|PRD

GRRRRLVWTPSQSEALRACFERNPYPGIATRERLAQAIGIPEPRVQIWFQNERSRQLRQH

>Human|DUX4L11|HD2|Dux|PRD

GRRKRTAVTGSQTALLLRAFEKDRFPGIAAREELARETGLPESRIQIWFQNRRARHPGQG

>Human|DUX4L12|HD1|Dux|PRD

GRRRRLVWTPSQSEALRACFERNPYPGIATRERLAQAIGIPEPRVQIWFQNERSRQLRQH

>Human|DUX4L12|HD2|Dux|PRD

GRRKRTAVTGFQTALLLRAFEKDRFPGIAAREELARETGLPESRIQIWFQNRRARHPGQG

>Human|DUX4L13|HD1|Dux|PRD

GRRRRLVWTPSQSEALRACFERNPYPGIATRERLAQAIGIPEPRVQIWFQNERSRQLRQH

>Human|DUX4L13|HD2|Dux|PRD

GRRKRTAVTGSQTALLLRAFEKDRFPGIAAREELARETGLPESRIQIWFQNRRARHPGQG

>Human|DUX4L14|HD1|Dux|PRD

GRRRRLVWTPSQSEALRACFERNPYPGIATRERLAQAIGIPEPRVQIWFQNERSRQLRQH

>Human|DUX4L14|HD2|Dux|PRD

GRRKRTAVTGSQTALLLRAFEKDRFPGIAAREELARETGLPESRIQIWFQNRRARHPGQG

>Human|DUX4L15|HD1|Dux|PRD

GRRKRTAVTGSQTALLLRAFEKDRFPGIAAREELARETGLPESRIQIWFQNRRARHPGQG

>Human|DUX4L15|HD2|Dux|PRD

GRRRRLVWTPSQSEALRACFERNPYPGIATRERLAQAIGIPEPRVQIWFQNERSRQLRQH

>Human|DUX4L2|HD1|Dux|PRD

GRRRRLVWTPSQSEALRACFERNPYPGIATRERLAQAIGIPEPRVQIWFQNERSRQLRQH

>Human|DUX4L2|HD2|Dux|PRD

GRRKRTAVTGSQTALLLRAFEKDRFPGIAAREELARETGLPESRIQIWFQNRRARHPGQG

>Human|DUX4L3|HD1|Dux|PRD

GRRRRLVWTPSQSEALRACFERNPYPGIATRERLAQAIGIPEPRVQIWFQNERSRQLRQH

>Human|DUX4L3|HD2|Dux|PRD

GRRKRTAVTGSQTALLLRAFEKDRFPGIAAREELARETGLPESRIQIWFQNRRARHPGQG

>Human|DUX4L4|HD1|Dux|PRD

GRRRRLVWTPSQSEALRACFERNPYPGIATRERLAQAIGIPEPRVQIWFQNERSRQLRQH

>Human|DUX4L4|HD2|Dux|PRD

GRRKRTAVTGSQTALLLRAFEKDRFPGIAAREELARETGLPESRIQIWFQNRRARHPGQG

>Human|DUX4L5|HD1|Dux|PRD

GRRRRLVWTPSQSEALRACFERNPYPGIATRERLAQAIGIPEPRVQIWFQNERSRQLRQH

>Human|DUX4L5|HD2|Dux|PRD

GRRKRTAVTGSQTALLLRAFEKDRFPGIAAREELARETGLPESRIQIWFQNRRARHPGQG

>Human|DUX4L6|HD1|Dux|PRD

GRRRRLVWTPSQSEALRACFERNPYPGIATRERLAQAIGIPEPRVQIWFQNERSRQLRQH

>Human|DUX4L6|HD2|Dux|PRD

GRRKRTAVTGSQTALLLRAFEKDRFPGIAAREELARETGLPESRIQIWFQNRRARHPGQG

>Human|DUX4L7|HD1|Dux|PRD

GRRRRLVWTPSQSEALRACFERNPYPGIATRERLAQAIGIPEPRVQIWFQNERSRQLRQH

>Human|DUX4L7|HD2|Dux|PRD

GRRKRTAVTGSQTALLLRAFEKDRFPGIAAREELARETGLPESRIQIWFQNRRARHPGQG

>Human|DUX4L8|HD1|Dux|PRD

GRRRRLVWTPSQSEALRACFERNPYPGIATRERLAQAIGIPEPRVQIWFQNERSRQLRQH

>Human|DUX4L8|HD2|Dux|PRD

GRRKRTAVTGSQTALLLRAFEKDRFPGIAAREELARETGLPESRIQIWFQNRRARHPGQG

>Human|DUX4L9|HD1|Dux|PRD

GRRRRLVWTPSQSEALRACFERNPYPGIATRERLAQAIGIPEPRVQIWFQNERSRQLRQH

>Human|DUX4L9|HD2|Dux|PRD

GRRKRTAVTGSQTALLLRAFEKDRFPGIAAREELARETGLPESRIQIWFQNRRARHPGQG

>Human|DUXA|HD1|Dux|PRD

HRRCRTKFTEEQLKILINTFNQKPYPGYATKQKLALEINTEESRIQIWFQNRRARHGFQK

>Human|DUXA|HD2|Dux|PRD

ARRCRTTYSASQLHTLIKAFMKNPYPGIDSREELAKEIGVPESRVQIWFQNRRSRLLLQR

>Human|DUXB|HD1|Dux|PRD

FWRNRIQYNQSQKDILQSWFQHDPFPDKAAREQLAKEIGVPESNIQVWFKNYRVKQRKLD

>Human|DUXB|HD2|Dux|PRD

ARQKQTFITWTQKNRLVQAFERNPFPDIATRKKLAEQTGLQESRIQMWFQKQRSLYLKKS

>Human|EMX1|Emx|ANTP

PKRIRTAFSPSQLLRLERAFEKNHYVVGAERKQLAGSLSLSETQVKVWFQNRRTKYKRQK

>Chicken|EMX1|Emx|ANTP

PKRIRTAFSPSQLLRLERAFEKNHYVVGAERKQLASSLSLSETQVKVWFQNRRTKYKRQK

>Zebrafish|emx1|Emx|ANTP

PKRIRTAFSPSQLLRLERAFEKNHYVVGAERKQLANSLSLSETQVKVWFQNRRTKYKRQK

>Human|EMX2|Emx|ANTP

PKRIRTAFSPSQLLRLEHAFEKNHYVVGAERKQLAHSLSLTETQVKVWFQNRRTKFKRQK

>Chicken|EMX2|Emx|ANTP

PKRIRTAFSPSQLLRLEHAFEKNHYVVGAERKQLAHSLSLTETQVKVWFQNRRTKFKRQK

>Zebrafish|emx2|Emx|ANTP

PKRIRTAFSPSQLLRLEHAFEKNHYVVGAERKQLAHSLSLTETQVKVWFQNRRTKFKRQK

>Zebrafish|emx3|Emx|ANTP

PKRIRTAFSPSQLLRLERAFEKNHYVVGAERKQLANGLCLTETQVKVWFQNRRTKHKRQK

>Human|EN1|En|ANTP

DKRPRTAFTAEQLQRLKAEFQANRYITEQRRQTLAQELSLNESQIKIWFQNKRAKIKKAT

>Chicken|EN1|En|ANTP

DKRPRTAFTAEQLQRLKAEFQANRYITEQRRQSLAQELSLNESQIKIWFQNKRAKIKKAT

>Human|EN2|En|ANTP

DKRPRTAFTAEQLQRLKAEFQTNRYLTEQRRQSLAQELSLNESQIKIWFQNKRAKIKKAT

>Zebrafish|eng1a|En|ANTP

DKRPRTAFTAEQLQRLKAEFQTSRYITEQRRQALARELGLNESQIKIWFQNKRAKIKKSS

>Zebrafish|eng1b|En|ANTP

RPRTAFTAEQLQRLKAEFQANRYITEQRRQSLAQELNLNESQIKIWFQNKRAKIKKAS

>Zebrafish|eng2a|En|ANTP

DKRPRTAFTAEQLQRLKAEFQTNRYLTEQRRQSLAQELGLNESQIKIWFQNKRAKIKKAS

>Zebrafish|eng2b|En|ANTP

DKRPRTAFTAEQLQRLKNEFQNNRYLTEQRRQALAQELGLNESQIKIWFQNKRAKIKKAT

>Human|ESX1|Esx|PRD

KRRRRTAFTQFQLQELENFFDESQYPDVVARERLAARLNLTEDRVQVWFQNRRAKWKRNQ

>Zebrafish|eve1|Evx|ANTP

RRHRTAFTREQLTRLEQEYCKESYVSRPRRCELAAALNLPETTIKVWFQNRRMKDKRQR

>Human|EVX1|Evx|ANTP

MRRYRTAFTREQIARLEKEFYRENYVSRPRRCELAAALNLPETTIKVWFQNRRMKDKRQR

>Chicken|EVX1|Evx|ANTP

MRRYRTAFTREQIARLEKEFYRENYVSRPRRCELAAALNLPETTIKVWFQNRRMKDKRQR

>Zebrafish|evx1|Evx|ANTP

MRRYRTAFTREQIARLEKEFYRENYVSRPRRCELAAALNLPETTIKVWFQNRRMKDKRQR

>Human|EVX2|Evx|ANTP

VRRYRTAFTREQIARLEKEFYRENYVSRPRRCELAAALNLPETTIKVWFQNRRMKDKRQR

>Chicken|EVX2|Evx|ANTP

VRRYRTAFTREQIARLEKEFYRENYVSRPRRCELAAALNLPETTIKV

>Zebrafish|evx2|Evx|ANTP

VRRYRTAFTREQIGRLEKEFYRENYVSRPRRCELAAALNLPETTIKVWFQNRRMKDKRQR

>Zebrafish|flh|Noto|ANTP

RMRTSFTNDQLSRLEKEFARQQYMVGSERFLLASALQLTEAQVKVWFQNRRIKWRKQS

>Human|GBX1|Gbx|ANTP

SRRRRTAFTSEQLLELEKEFHCKKYLSLTERSQIAHALKLSEVQVKIWFQNRRAKWKRIK

>Chicken|GBX1|Gbx|ANTP

SRRRRTAFTSEQLLELEKEFHCKKYLSLTERSQIAHALKLSEVQVKIWFQNRRAKWKRIK

>Zebrafish|gbx1|Gbx|ANTP

SRRRRTAFTSEQLLELEKEFHCKKYLSLTERSQIAHALKLSEVQVKIWFQNRRAKWKRIK

>Human|GBX2|Gbx|ANTP

NRRRRTAFTSEQLLELEKEFHCKKYLSLTERSQIAHALKLSEVQVKIWFQNRRAKWKRVK

>Chicken|GBX2|Gbx|ANTP

NRRRRTAFTSEQLLELEKEFHCKKYLSLTERSQIAHALKLSEVQVKIWFQNRRAKWKRVK

>Zebrafish|gbx2|Gbx|ANTP

NRRRRTAFTSEQLLELEKEFHCKKYLSLTERSQIAHALKLSEVQVKIWFQNRRAKWKRVK

>Chicken|gga_chr02.1|Hox5|ANTP

KRARTAYTRYQTLELEKEFHFNRYLTRRRRIEIAHALCLSERQIKIWFQNRRMKWKKD

>Chicken|gga_chr03.1|Hlx|ANTP

SRAVFSNLQRKGLEKRFEIQKYVTKPDRKQLAAMLGLTDAQV

>Chicken|GNOT1|Noto|ANTP

KRVRTVFKPEQLERLEQEFLKQQYMVGTERVDLAATLRLTETQVKVWFQNRRIKWRKQS

>Human|GSC|Gsc|PRD

KRRHRTIFTDEQLEALENLFQETKYPDVGTREQLARKVHLREEKVEVWFKNRRAKWRRQK

>Chicken|GSC|Gsc|PRD

KRRHRTIFTDEQLEALENLFQETKYPDVGTREQLARKVHLREEKVEVWFKNRRAKWRRQK

>Zebrafish|gsc|Gsc|PRD

KRRHRTIFTDEQLEALENLFQETKYPDVGTREQLARKVHLREEKVEVWFKNRRAKWRRQK

>Human|GSC2|Gsc|PRD

TRRHRTIFSEEQLQALEALFVQNQYPDVSTRERLAGRIRLREERVEVWFKNRRAKWRHQK

>Chicken|GSC2|Gsc|PRD

TRRHRTIFTEEQLQALETLFHQNQYPDVITREHLANRIHLKEERVEVWFKNRRAKWRHQK

>Human|GSX1|Gsx|ANTP

SKRMRTAFTSTQLLELEREFASNMYLSRLRRIEIATYLNLSEKQVKIWFQNRRVKHKKEG

>Zebrafish|gsx1|Gsx|ANTP

SKRMRTAFTSTQLLELEREFTSNMYLSRLRRIEIATYLNLSEKQVKIWFQNRRVKHKKEG

>Human|GSX2|Gsx|ANTP

GKRMRTAFTSTQLLELEREFSSNMYLSRLRRIEIATYLNLSEKQVKIWFQNRRVKHKKEG

>Zebrafish|gsx2|Gsx|ANTP

GKRMRTAFTSTQLLELEREFSSNMYLSRLRRIEIATYLNLSEKQVKIWFQNRRVKHKKEG

>Human|HDX|Hdx|POU

SRKRALQDRTQFSDRDLATLKKYWDNGMTSLGSVCREKIEAVATELNVDCEIVRTWIGNRRRKYRLMG

>Chicken|HDX|HD1|Hdx|POU

RSVFTVEQQRILQRYYENGMTNQSKNCFQLILQCAQETKLDFSVVRTWVGNKRRKM

>Chicken|HDX|HD2|Hdx|POU

SRKRALQDRTQFSDRDLATLKKYWDNGMTSLGSVCREKIEAVAAELNVDCEIVRTWIGNRRRKYRLMG

>Human|HESX1|Hesx|PRD

GRRPRTAFTQNQIEVLENVFRVNCYPGIDIREDLAQKLNLEEDRIQIWFQNRRAKLKRSH

>Chicken|HESX1|Hesx|PRD

GRRPRTAFTRNQIEVLENVFKMNSYPGIDIREELARKLDLEEDRIQIWFQNRRAKLKRSH

>Zebrafish|hesx1|Hesx|PRD

GRRPRTAFSSVQIKILESVFQVNSYPGIDIREELAKKLQLDEDRIQIWFQNRRAKLKRSH

>Human|HHEX|Hhex|ANTP

RKGGQVRFSNDQTIELEKKFETQKYLSPPERKRLAKMLQLSERQVKTWFQNRRAKWRRLK

>Chicken|HHEX|Hhex|ANTP

RKGGQVRFSNEQTIELEKKFETQKYLSPPERKRLAKLLQLSERQVKTWFQNRRAKWRRLK

>Zebrafish|hhex|Hhex|ANTP

RKGGQVRFSNDQTIELEKKFETQKYLSPPERKRLAKMLQLSERQVKTWFQNRRAKWRRLK

>Human|HLX|Hlx|ANTP

RSWSRAVFSNLQRKGLEKRFEIQKYVTKPDRKQLAAMLGLTDAQVKVWFQNRRMKWRHSK

>Zebrafish|hlx1|Hlx|ANTP

RSWSRAVFSNLQRKGLEKRFEIQKYVTKPDRKQLAAMLGLTDAQVKVWFQNRRMKWRHSK

>Zebrafish|hlxb9la|Mnx|ANTP

CRRPRTAFTSQQLLELENQFKLNKYLSRPKRFEVATSLMLTETQVKIWFQNRRMKWKRSR

>Zebrafish|hlxb9lb|Mnx|ANTP

CRRPRTAFTSQQLLELENQFKLNKYLSRPKRFEVATSLMLTETQVKIWFQNRRMKWKRSR

>Human|HMBOX1|Hmbox|HNF

RRGSRFTWRKECLAVMESYFNENQYPDEAKREEIANACNAVIQKPGKKLSDLERVTSLKVYNWFANRRKEIKRRA

>Chicken|HMBOX1|Hmbox|HNF

RRGSRFTWRKECLAVMESYFNENQYPDEAKREEIANACNAVIQKPGKKLSDLERVTSLKVYNWFANRRKEIKRRA

>Zebrafish|hmbox1a|Hmbox|HNF

RRGSRFTWRKECLAVMESYFNDNQYPDEAKREEIANACNAVIQKPGKKLSDLERVTSLKVYNWFANRRKEIKRRA

>Zebrafish|hmbox1b|Hmbox|HNF

RRGSRFTWRKECLAVMESYFSDNQYPDEAKREEIANACNAVIQKPGKKLSDLERVTSLKVYNWFANRRKEIKRRA

>Human|HMX1|Nk5/Hmx|ANTP

KKKTRTVFSRSQVFQLESTFDLKRYLSTAERAGLAASLQLTETQVKIWFQNRRNKWKRHV

>Chicken|HMX1|Nk5/Hmx|ANTP

KKKTRTVFSRSQVFQLESTFDVKRYLSSSERAGLAASLHLTETQVKIWFQNRRNKWKRQL

>Zebrafish|hmx1|Nk5/Hmx|ANTP

KKKTRTVFSRSQVFQLESTFDMKRYLSSSERAGLAASLHLTETQVKIWFQNRRNKWKRQL

>Human|HMX2|Nk5/Hmx|ANTP

KKKTRTVFSRSQVYQLESTFDMKRYLSSSERACLASSLQLTETQVKTWFQNRRNKWKRQL

>Chicken|HMX2|Nk5/Hmx|ANTP

KKKTRTVFSRSQVYQLESTFDMKRYLSSSERACLASSLQLTETQVKTWFQNRRNKWKRQL

>Zebrafish|hmx2|Nk5/Hmx|ANTP

KKKTRTVFSRSQVYQLESTFDMKRYLSSSERACLASSLQLTETQVKTWFQNRRNKWKRQL

>Chicken|HMX2-like|Nk5/Hmx|ANTP

KKKTRTVFSRSQVYQLESTFDMKRYLSSSERACLASSLQLTETQVKTWFQNRRNKWKRQL

>Human|HMX3|Nk5/Hmx|ANTP

KKKTRTVFSRSQVFQLESTFDMKRYLSSSERAGLAASLHLTETQVKIWFQNRRNKWKRQL

>Chicken|HMX3|Nk5/Hmx|ANTP

KKKTRTVFSRSQVFQLESTFDMKRYLSSSERAGLAASLHLTETQVKIWFQNRRNKWKRQL

>Zebrafish|hmx3|Nk5/Hmx|ANTP

KKKTRTVFSRSQVFQLESTFDMKRYLSSSERAGLAASLHLTETQVKIWFQNRRNKWKRQL

>Zebrafish|hmx4|Nk5/Hmx|ANTP

KKKTRTIFSKRQIFQLESTFDMKRYLSSAERACLANSLQLTETQVKIWFQNRRNKLKRQL

>Human|HNF1A|Hnf1|HNF

GRRNRFKWGPASQQILFQAYERQKNPSKEERETLVEECNRAECIQRGVSPSQAQGLGSNLVTEVRVYNWFANRRKEEAFRH

>Chicken|HNF1A|Hnf1|HNF

GRRNRFKWGPASQQILFQAYERQKNPSKEEREALVEECNRAECIQRGVSPSQAQGLGSNLVTEVRVYNWFANRRKEEAFRH

>Zebrafish|hnf1a|Hnf1|HNF

GRRNRFKWGPASLQILFQAYERQKNPSKEEREGLVEECNRAECLQRGVSPSQLAGLGSNLVTEVRVYNWFANRRKEEAFRH

>Human|HNF1B|Hnf1|HNF

MRRNRFKWGPASQQILYQAYDRQKNPSKEEREALVEECNRAECLQRGVSPSKAHGLGSNLVTEVRVYNWFANRRKEEAFRQ

>Chicken|HNF1B|HD1|Hnf1|HNF

RRNRFKWGPASQQILYQAYDRQKNPSKEEREALVEECNR

>Chicken|HNF1B|HD2|Hnf1|HNF

HRAECLQRGVSPSKAHGLGSNLVTEVRVYNWFANRRKEEAFRQ

>Zebrafish|hnf1ba|Hnf1|HNF

LRRNRFKWGPASQQILYQAYERQKNPSKEEREALVEECNRAECLQRGVSPSKAHGLGSNLVTEVRVYNWFANRRKEEAFRQ

>Zebrafish|hnf1bb|Hnf1|HNF

MRRNRFKWGPASQEILYQAYERQKNPSKEEREALVEECNRAECVQRGVSPSKAHGLGSNLVTEVRVYNWFANRRKEEAFRQ

>Human|HOMEZ|HD1|Zhx/Homez|ZF

WTQAAQTSELDSNEHLLKTFSYFPYPSLADIALLCLRYGLQMEKVKTWFMAQRLRCGISW

>Human|HOMEZ|HD2|Zhx/Homez|ZF

RQRKTKRKTKEQLAILKSFFLQCQWARREDYQKLEQITGLPRPEIIQWFGDTRYALKHGQ

>Human|HOMEZ|HD3|Zhx/Homez|ZF

TPPLPIPPPPPDIQPLERYWAAHQQLRETDIPQLSQASRLSTQQVLDWFDSRLPQPAEVV

>Zebrafish|homez|HD1|Zhx/Homez|ZF

WTQSDQTRELDSIPELVQAFNIFPYPTSQEVSTLARVCTLPLDKVKVWFMVQRIKYGISW

>Zebrafish|homez|HD2|Zhx/Homez|ZF

KKSKAQLTALRKSFLQENWPDETELQRLQDETSLTRNEIRKWFSDSRYQLRMGR

>Zebrafish|homez|HD3|Zhx/Homez|ZF

RKTKEQLTILKEYFQHCPWPKSGEYTEVVELTSLPRADVIQWFGDTRYAVKNG

>Zebrafish|homez|HD4|Zhx/Homez|ZF

DVSPLELYFRQTGPLQEKDLDNLCRKSKMSYQQVRDWFASK

>Human|HOPX|Hopx|PRD

SAETASGPTEDQVEILEYNFNKVDKHPDSTTLCLIAAEAGLSEEETQKWFKQRLAKWRRSE

>Chicken|HOPX|Hopx|PRD

ATEKSVTPTEEQLEILEYNFCKVNKHPDPTTLCLIAAETGLSEEQTLKWFKQRLAEWRKSE

>Chicken|Hox-10|Hox9-13(15)|ANTP

RKKRCPYTKHQTLELEKEFLFNMYLTRERRLEISKSINLTDRQVKIWFQNRRMKLKKM

>Chicken|Hox-6|Hox6-8|ANTP

ELEKEFHFNRYLTRRRRIEIAHSLCLTERQIKIWFQNRRMKWKKE

>Chicken|Hox-7|Hox6-8|ANTP

RKRGRQTYTRYQTLELEKEFHYNRYLTRRRRIEIAHALCLTERQIKIWFQNRRMKWKKE

>Human|HOXA1|Hox1|ANTP

PNAVRTNFTTKQLTELEKEFHFNKYLTRARRVEIAASLQLNETQVKIWFQNRRMKQKKRE

>Chicken|HOXA1|Hox1|ANTP

VRTNFTTKQLTELEKEFHFNKYLTRARRVEIAASLQLNETQVKIWFQNRRMKQKKRE

>Human|HOXA10|Hox9-13(15)|ANTP

GRKKRCPYTKHQTLELEKEFLFNMYLTRERRLEISRSVHLTDRQVKIWFQNRRMKLKKMN

>Chicken|HOXA10|Hox9-13(15)|ANTP

GRKKRCPYTKHQTLELEKEFLFNMYLTRERRLEISRTVHLTDRQVKIWFQNRRMKLKKMN

>Zebrafish|hoxa10b|Hox9-13(15)|ANTP

GRKKRCPYTKHQTLELEKEFLFNMYLTRERRLEISRSVHLTDRQVKIWFQNRRMKLKKMS

>Human|HOXA11|Hox9-13(15)|ANTP

TRKKRCPYTKYQIRELEREFFFSVYINKEKRLQLSRMLNLTDRQVKIWFQNRRMKEKKIN

>Chicken|HOXA11|Hox9-13(15)|ANTP

TRKKRCPYTKYQIRELEREFFFSVYINKEKRLQLSRMLNLTDRQVKIWFQNRRMKEKKIN

>Zebrafish|hoxa11a|Hox9-13(15)|ANTP

RKKRCPYTKFQIRELEREFFFSVYINKEKRLQLSRMLNLTDRQVKMWFQNRRMKEKKLN

>Zebrafish|hoxa11b|Hox9-13(15)|ANTP

TRKKRCPYTKYQIRELEREFFFSVYINKEKRLQLSRMLNLTDRQVKIWFQNRRMKEKKLN

>Human|HOXA13|Hox9-13(15)|ANTP

GRKKRVPYTKVQLKELEREYATNKFITKDKRRRISATTNLSERQVTIWFQNRRVKEKKVI

>Zebrafish|hoxa13a|Hox9-13(15)|ANTP

GRKKRVPYTKVQLKELEREYATNKFITKDKRRRISAQTNLSERQVTIWFQNRRVKEKKVV

>Zebrafish|hoxa13b|Hox9-13(15)|ANTP

RKKRVPYTKVQLKELEREYATNKFITKDKRRRISAHTNLTERQVTIWFQNRRVKEKK

>Zebrafish|hoxa1a|Hox1|ANTP

PNTVRTNFSTKQLTELEKEFHFNKYLTRARRVEIAASLQLNETQVKIWFQNRRMKQKKRE

>Human|HOXA2|Hox2|ANTP

SRRLRTAYTNTQLLELEKEFHFNKYLCRPRRVEIAALLDLTERQVKVWFQNRRMKHKRQT

>Zebrafish|hoxa2b|Hox2|ANTP

TRRLRTAYTNTQLLELEKEFHFNKYLCRPRRVEIAALLDLTERQVKVWFQNRRMKHKRQT

>Human|HOXA3|Hox3|ANTP

SKRARTAYTSAQLVELEKEFHFNRYLCRPRRVEMANLLNLTERQIKIWFQNRRMKYKKDQ

>Chicken|HOXA3|Hox3|ANTP

SKRARTAYTSAQLVELEKEFHFNRYLCRPRRVEMANLLNLTERQIKIWFQNRRMKYKKDQ

>Zebrafish|hoxa3a|Hox3|ANTP

SKRARTAYTSAQLVELEKEFHFNRYLCRPRRVEMANLLNLTERQIKIWFQNRRMKYKKDQ

>Human|HOXA4|Hox4|ANTP

PKRSRTAYTRQQVLELEKEFHFNRYLTRRRRIEIAHTLCLSERQVKIWFQNRRMKWKKDH

>Chicken|HOXA4|Hox4|ANTP

PKRSRTAYTRQQVLELEKEFHFNRYLTRRRRIEIAHTLCLSERQVKIWFQNRRMKWKKDH

>Zebrafish|hoxa4a|Hox4|ANTP

PKRSRTAYTRQQALELEKEFHFNRYLTRRRRVEIAHTMCLSERQVKIWFQNRRMKWKKDH

>Human|HOXA5|Hox5|ANTP

GKRARTAYTRYQTLELEKEFHFNRYLTRRRRIEIAHALCLSERQIKIWFQNRRMKWKKDN

>Zebrafish|hoxa5a|Hox5|ANTP

GKRPRTAYTRYQTLELEKEFHFNRYLTRRRRIEIAHTLCLSERQIKIWFQNRRMKWKKDN

>Human|HOXA6|Hox6-8|ANTP

GRRGRQTYTRYQTLELEKEFHFNRYLTRRRRIEIANALCLTERQIKIWFQNRRMKWKKEN

>Chicken|HOXA6|Hox6-8|ANTP

LELEKEFHFNRYLTRRRRIEIANALCLTERQIKIWFQNRRMKWKKEN

>Human|HOXA7|Hox6-8|ANTP

RKRGRQTYTRYQTLELEKEFHFNRYLTRRRRIEIAHALCLTERQIKIWFQNRRMKWKKEH

>Chicken|HOXA7|Hox6-8|ANTP

RKRGRQTYTRYQTLELEKEFHFNRYLTRRRRIEIAHALCLTERQIKIWFQNRRMKW

>Human|HOXA9|Hox9-13(15)|ANTP

TRKKRCPYTKHQTLELEKEFLFNMYLTRDRRYEVARLLNLTERQVKIWFQNRRMKMKKIN

>Chicken|HOXA9|Hox9-13(15)|ANTP

TRKKRCPYTKHQTLELEKEFLFNMYLTRDRRYEVARLLNLTERQVKIWFQNRRMKMKKIN

>Zebrafish|hoxa9a|Hox9-13(15)|ANTP

TRKKRCPYTKHQILELEKEFLFNTYLTRDRRYEVARLLNLTERQVKIWFQNRRMKMKKFN

>Zebrafish|hoxa9b|Hox9-13(15)|ANTP

TRKKRCPYTKHQTLELEKEFLFNMYLSRDRRYEVARLLNLTERQVKIWFQNRRMKMKKCN

>Human|HOXB1|Hox1|ANTP

PSGLRTNFTTRQLTELEKEFHFNKYLSRARRVEIAATLELNETQVKIWFQNRRMKQKKRE

>Chicken|HOXB1|Hox1|ANTP

PNTIRTNFTTKQLTELEKEFHFNKYLTRARRVEIAATLELNETQVKIWFQNRRMKQKKRE

>Zebrafish|hoxb10a|Hox9-13(15)|ANTP

GRKKRCPYSKHQILELEKEFLFNMYLTRERRLEISRSINLTDRQVKIWFQNRRMKLKKMT

>Human|HOXB13|Hox9-13(15)|ANTP

GRKKRIPYSKGQLRELEREYAANKFITKDKRRKISAATSLSERQITIWFQNRRVKEKKVL

>Chicken|HOXB13|Hox9-13(15)|ANTP

GRKKRVPYSKGQLKELEKEYASSKFITRDKRRKISAATNLTERQITIWFQNRRVKEKKVV

>Zebrafish|hoxb13a|Hox9-13(15)|ANTP

GRKKRIPYTKVQLKELEKEYAANKFITKDKRRKISAVTNLSERQITIWFQNRRVKEKKFI

>Zebrafish|hoxb1a|Hox1|ANTP

NTIRTNFTTKQLTELEKEFHFSKYLTRARRVEIAATLELNETQVKIWFQNRRMKQKKRE

>Zebrafish|hoxb1b|Hox1|ANTP

NIIRTNFTTKQLTELEKEFHFNKYLTRARRVEVAATLELNETQVKIWFQNRRMKQKKRE

>Human|HOXB2|Hox2|ANTP

ARRLRTAYTNTQLLELEKEFHFNKYLCRPRRVEIAALLDLTERQVKVWFQNRRMKHKRQT

>Chicken|HOXB2|Hox2|ANTP

SRRLRTAYTNTQLLELEKEFHFNKYLCRPRRVEIAALLDLTERQVKVWFQNRRMKHKRQT

>Zebrafish|hoxb2a|Hox2|ANTP

SRRLRTAYTNTQLLELEKEFHFNKYLCRPRRVEIAALLDLTERQVKVWFQNRRMKHKRQT

>Human|HOXB3|Hox3|ANTP

SKRARTAYTSAQLVELEKEFHFNRYLCRPRRVEMANLLNLSERQIKIWFQNRRMKYKKDQ

>Chicken|HOXB3|Hox3|ANTP

SKRARTAYTSAQLVELEKEFHFNRYLCRPRRVEMANLLNLSERQIKIWFQNRRMKYKKDQ

>Zebrafish|hoxb3a|Hox3|ANTP

SKRARTAYTSAQLVELEKEFHFNRYLCRPRRVEMANLLNLSERQIKIWFQNRRMKYKKDQ

>Human|HOXB4|Hox4|ANTP

PKRSRTAYTRQQVLELEKEFHYNRYLTRRRRVEIAHALCLSERQIKIWFQNRRMKWKKDH

>Zebrafish|hoxb4a|Hox4|ANTP

PKRSRTAYTRQQVLELEKEFHYNRYLTRRRRVEIAHTLCLSERQIKIWFQNRRMKWKKDH

>Human|HOXB5|Hox5|ANTP

GKRARTAYTRYQTLELEKEFHFNRYLTRRRRIEIAHALCLSERQIKIWFQNRRMKWKKDN

>Chicken|HOXB5|Hox5|ANTP

GKRARTAYTRYQTLELEKEFHFNRYLTRRRRIEIAHALCLSERQIKIWFQNRRMKWKKDN

>Zebrafish|hoxb5a|Hox5|ANTP

GKRARTAYTRYQTLELEKEFHFNRYLTRRRRIEIAHALCLSERQIKIWFQNRRMKWKKDN

>Zebrafish|hoxb5b|Hox5|ANTP

GKRARTAYTRYQTLELEKEFHFNRYLTRRRRIEIAHALCLSERQIKIWFQNRRMKWKKDN

>Human|HOXB6|Hox6-8|ANTP

GRRGRQTYTRYQTLELEKEFHYNRYLTRRRRIEIAHALCLTERQIKIWFQNRRMKWKKES

>Zebrafish|hoxb6a|Hox6-8|ANTP

GRRGRQTYTRYQTLELEKEFHFNRYLTRRRRIEIAHALCLTERQIKIWFQNRRMKWKKEN

>Zebrafish|hoxb6b|Hox6-8|ANTP

GRRGRQTYTRFQTLELEKEFHFNRYLTRRRRIEISHALCLTERQIKIWFQNRRMKWKKEN

>Human|HOXB7|Hox6-8|ANTP

RKRGRQTYTRYQTLELEKEFHYNRYLTRRRRIEIAHTLCLTERQIKIWFQNRRMKWKKEN

>Zebrafish|hoxb7a|Hox6-8|ANTP

RKRGRQTYSRYQTLELEKEFHFNRYLSRRRRIEIAHALCLTERQIKIWFQNRRMKWKKEN

>Human|HOXB8|Hox6-8|ANTP

RRRGRQTYSRYQTLELEKEFLFNPYLTRKRRIEVSHALGLTERQVKIWFQNRRMKWKKEN

>Chicken|HOXB8|Hox6-8|ANTP

RRRGRQTYSRYQTLELEKEFLFNPYLTRKRRIEVSHALGLTERQVKIWFQNRRMKWKKEN

>Zebrafish|hoxb8a|Hox6-8|ANTP

RRRGRQTYSRYQTLELEKEFLFNPYLTRKRRIEVSHALGLTERQVKIWFQNRRMKWKKEN

>Zebrafish|hoxb8b|Hox6-8|ANTP

RRRGRQTYSRYQTLELEKEFLFNPYLTRKRRIEVSHALALTERQVKIWFQNRRMKWKKEH

>Human|HOXB9|Hox9-13(15)|ANTP

SRKKRCPYTKYQTLELEKEFLFNMYLTRDRRHEVARLLNLSERQVKIWFQNRRMKMKKMN

>Chicken|HOXB9|Hox9-13(15)|ANTP

SRKKRCPYTKYQTLELEKEFLFNMYLTRDRRHEVARLLNLSERQVKIWFQNRRMKMKKMN

>Zebrafish|hoxb9a|Hox9-13(15)|ANTP

SRKKRCPYTKYQTLELEKEFLFNMYLTRDRRHEVARLLNLTERQVKIWFQNRRMKMKKMN

>Human|HOXC10|Hox9-13(15)|ANTP

GRKKRCPYTKHQTLELEKEFLFNMYLTRERRLEISKTINLTDRQVKIWFQNRRMKLKKMN

>Zebrafish|hoxc10a|Hox9-13(15)|ANTP

RKKRCPYTKHQTLELEKEFLFNMYLTRERRLEISKSINLTDRQVKIWFQNRRMKLKK

>Human|HOXC11|Hox9-13(15)|ANTP

TRKKRCPYSKFQIRELEREFFFNVYINKEKRLQLSRMLNLTDRQVKIWFQNRRMKEKKLS

>Chicken|Hoxc11|Hox9-13(15)|ANTP

TRKKRCPYSKFQIRELEREFFFNVYINKEKRLQLSRMLNLTDRQVKIWFQNRRMKEKK

>Zebrafish|hoxc11a|Hox9-13(15)|ANTP

TRKKRCPYSKFQIRELEREFFFNVYINKEKRLQLSRMLNLTDRQVKIWFQNRRMKEKKLS

>Zebrafish|Hoxc11b|Hox9-13(15)|ANTP

RKKRCPYTKFQIRELEREFFFNVYINKEKRLQLSRILNLTDRQVKIWFQNRRMKEKKLS

>Human|HOXC12|Hox9-13(15)|ANTP

SRKKRKPYSKLQLAELEGEFLVNEFITRQRRRELSDRLNLSDQQVKIWFQNRRMKKKRLL

>Zebrafish|hoxc12a|Hox9-13(15)|ANTP

TRKKRKPYSKLQLAELEGEFMLNEFITRQRRRELSDRLNLSDQQVKIWFQNRRMKKKRLL

>Zebrafish|hoxc12b|Hox9-13(15)|ANTP

TRKKRKPYSKLQLNELEGEFILNEFITRQRRRELSDRLNLTDQQVKIWFQNRRMKKKRLL

>Human|HOXC13|Hox9-13(15)|ANTP

GRKKRVPYTKVQLKELEKEYAASKFITKEKRRRISATTNLSERQVTIWFQNRRVKEKKVV

>Chicken|Hoxc13|Hox9-13(15)|ANTP

RKKRVPYTKIQLKELEKEYAASKFITKEKRRRISATTNLSERQVTIWFQNRRVKEKK

>Zebrafish|hoxc13a|Hox9-13(15)|ANTP

GRKKRVPYTKIQLKELEKEYAASKFITKDKRRRISATTNLSERQVTIWFQNRRVKEKKFV

>Zebrafish|hoxc13b|Hox9-13(15)|ANTP

GRKKRVPYTKIQLKELEKEYAASKFITKDRRRRISATTSLSERQVTIWFQNRRVKEKKFV

>Zebrafish|hoxc1a|Hox1|ANTP

SGGSRTNFTTKQLTELEKEFHFNKYLTRARRIEIANPLQLSETQVKIWFQNRRMKQKK

>Zebrafish|hoxc3a|Hox3|ANTP

SKRARVAFTSSQLLELEKEFHFSAYLCRNRRLEMAELLKLTDRQIKIWFQNRRMKYKKD

>Human|HOXC4|Hox4|ANTP

PKRSRTAYTRQQVLELEKEFHYNRYLTRRRRIEIAHSLCLSERQIKIWFQNRRMKWKKDH

>Chicken|Hoxc4|Hox4|ANTP

KRSRTAYTRQQVLELEKEFHYNRYLTRRRRVEIAHSLCLSERQIKIWFQNRRMKWKKD

>Zebrafish|hoxc4a|Hox4|ANTP

PKRSRTAYTRQQVLELEKEFHYNRYLTRRRRIEIAHSLVLSERQIKIWFQNRRMKWKKDH

>Human|HOXC5|Hox5|ANTP

GKRSRTSYTRYQTLELEKEFHFNRYLTRRRRIEIANNLCLNERQIKIWFQNRRMKWKKDS

>Zebrafish|hoxc5a|Hox5|ANTP

GKRSRTSYTRYQTLELEKEFHFNRYLTRRRRIEIANNLCLNERQIKIWFQNRRMKWKKDS

>Human|HOXC6|Hox6-8|ANTP

RRRGRQIYSRYQTLELEKEFHFNRYLTRRRRIEIANALCLTERQIKIWFQNRRMKWKKES

>Zebrafish|hoxc6a|Hox6-8|ANTP

RRRGRQIYSRYQTLELEKEFHFNRYLTRRRRIEIANALCLTERQIKIWFQNRRMKWKKET

>Zebrafish|hoxc6b|Hox6-8|ANTP

RRRGRQIYSRYQTLELEKEFHYNRYLTRRRRIEIANTLCLSERQIKIWFQNRRMKWKKES

>Human|HOXC8|Hox6-8|ANTP

RRSGRQTYSRYQTLELEKEFLFNPYLTRKRRIEVSHALGLTERQVKIWFQNRRMKWKKEN

>Zebrafish|hoxc8a|Hox6-8|ANTP

RRNGRQTYSRYQTLELEKEFLFNPYLTRKRRIEVSHALSLTERQVKIWFQNRRMKWKKEN

>Human|HOXC9|Hox9-13(15)|ANTP

TRKKRCPYTKYQTLELEKEFLFNMYLTRDRRYEVARVLNLTERQVKIWFQNRRMKMKKMN

>Chicken|Hoxc9|Hox9-13(15)|ANTP

TRKKRCPYTKYQTLELEKEFLFNMYLTRDRRYEVARVLNLTERQVKIWFQNRGMKMKKM

>Zebrafish|hoxc9a|Hox9-13(15)|ANTP

TRKKRCPYTKYQTLELEKEFLFNMYLTRDRRYEVARVLNLTERQVKIWFQNRRMKMKKMN

>Human|HOXD1|Hox1|ANTP

SSAIRTNFSTKQLTELEKEFHFNKYLTRARRIEIANCLHLNDTQVKIWFQNRRMKQKKRE

>Chicken|HOXD1|Hox1|ANTP

SLRTSFSTRQLTELEKEFHFSRYLSRARRLEVARSLRLRDAQVKVWFQNRRMKQKKRE

>Human|HOXD10|Hox9-13(15)|ANTP

GRKKRCPYTKHQTLELEKEFLFNMYLTRERRLEISKSVNLTDRQVKIWFQNRRMKLKKMS

>Zebrafish|hoxd10a|Hox9-13(15)|ANTP

GRKKRCPYTKHQTLELEKEFLFNMYLTRERRLEISKSVNLTDRQVKIWFQNRRMKLKKMS

>Human|HOXD11|Hox9-13(15)|ANTP

SRKKRCPYTKYQIRELEREFFFNVYINKEKRLQLSRMLNLTDRQVKIWFQNRRMKEKKLN

>Chicken|HOXD11|Hox9-13(15)|ANTP

SRKKRCPYTKYQIRELEREFFFNVYINKEKRLQLSRMLNLTDRQVKIWFQNRRMKEKKLN

>Zebrafish|hoxd11a|Hox9-13(15)|ANTP

SRKKRCPYSKYQIRELEREFFFNVYINKEKRLQLSRMLSLTDRQVKIWFQNRRMKEKKLN

>Human|HOXD12|Hox9-13(15)|ANTP

ARKKRKPYTKQQIAELENEFLVNEFINRQKRKELSNRLNLSDQQVKIWFQNRRMKKKRVV

>Chicken|HOXD12|Hox9-13(15)|ANTP

SRKKRKPYTKQQIAELENEFLLNEFINRQKRKELSNRLNLSDQQVKIWFQNRRMKKKRVV

>Zebrafish|hoxd12a|Hox9-13(15)|ANTP

SRKKRKPYTKPQLTELENEFMMNEFINRQKRKELSDRLELSDQQVKIWFQNRRMKKKRLM

>Human|HOXD13|Hox9-13(15)|ANTP

GRKKRVPYTKLQLKELENEYAINKFINKDKRRRISAATNLSERQVTIWFQNRRVKDKKIV

>Chicken|HOXD13|Hox9-13(15)|ANTP

GRKKRVPYTKLQLKELENEYAINKFINKDKRRRISAATNLSERQVTIWFQNRRVKDKKIV

>Zebrafish|hoxd13a|Hox9-13(15)|ANTP

GRKKRVPYTKFQLKELEREYNTTKFITKENRRRIASSTNLSERQVTIWFQNRRVKDKK

>Human|HOXD3|Hox3|ANTP

SKRVRTAYTSAQLVELEKEFHFNRYLCRPRRVEMANLLNLTERQIKIWFQNRRMKYKKDQ

>Chicken|HOXD3|Hox3|ANTP

SKRVRTAYTSAQLVELEKEFHFNRYLCRPRRVEMANLLNLTERQIKIWFQNRRMKYKKDQ

>Zebrafish|hoxd3a|Hox3|ANTP

SKRVRTAYTSAQLVELEKEFHFNRYLCRPRRVEMANLLNLTERQIKIWFQNRRMKYKKDQ

>Human|HOXD4|Hox4|ANTP

PKRSRTAYTRQQVLELEKEFHFNRYLTRRRRIEIAHTLCLSERQIKIWFQNRRMKWKKDH

>Chicken|HOXD4|Hox4|ANTP

PKRSRTAYTRQQVLELEKEFHFNRYLTRRRRIEIAHTLCLSERQIKIWFQNRRMKWKKDH

>Zebrafish|hoxd4a|Hox4|ANTP

PKRSRTAYTRQQVLELEKEFHFNRYLTRRRRIEIAHTLCLSERQIKIWFQNRRMKWKKDH

>Human|HOXD8|Hox6-8|ANTP

RRRGRQTYSRFQTLELEKEFLFNPYLTRKRRIEVSHALALTERQVKIWFQNRRMKWKKEN

>Chicken|HOXD8|Hox6-8|ANTP

TYSRFQTLELEKEFLFNPYLTRKRRIEVSHALGLTERQVKIWFQNRRMKWKKEN

>Human|HOXD9|Hox9-13(15)|ANTP

TRKKRCPYTKYQTLELEKEFLFNMYLTRDRRYEVARILNLTERQVKIWFQNRRMKMKKMS

>Zebrafish|hoxd9a|Hox9-13(15)|ANTP

TRKKRCPYTKYQTLELEKEFLYNMYLTRDRRYEVARILNLTERQVKIWFQNRRMKMKKMN

>Human|IRX1|Irx|TALE

DPGRPKNATRESTSTLKAWLNEHRKNPYPTKGEKIMLAIITKMTLTQVSTWFANARRRLKKEN

>Zebrafish|irx1a|Irx|TALE

DPARPKNATRESTSTLKAWLNEHRKNPYPTKGEKIMLAIITKMTLTQVSTWFANARRRLKKEN

>Zebrafish|irx1b|Irx|TALE

DPARAKSATRETTSTLKAWLQEHKKNPYPTKGEKIMLAIITKMTLTQVSTWFANARRRLKKEN

>Human|IRX2|Irx|TALE

DPAYRKNATRDATATLKAWLNEHRKNPYPTKGEKIMLAIITKMTLTQVSTWFANARRRLKKEN

>Chicken|IRX2|Irx|TALE

DPAYRKNATRDATATLKAWLQEHRKNPYPTKGEKIMLAIITKMTLTQVSTWFANARRRLKKEN

>Zebrafish|irx2a|Irx|TALE

DPAYRKNATRDATATLKAWLQEHRKNPYPTKGQKIMLAIITKMTLTQVSTWFANARRRLKKEN

>Human|IRX3|Irx|TALE

DPSRPKNATRESTSTLKAWLNEHRKNPYPTKGEKIMLAIITKMTLTQVSTWFANARRRLKKEN

>Zebrafish|irx3a|Irx|TALE

DPSRPKNATRESTSTLKAWLSEHRKNPYPTKGEKIMLAIITKMTLTQVSTWFANARRRLKKEN

>Zebrafish|irx3b|Irx|TALE

DPSRPKNATRESTSTLKAWLSEHRKNPYPTKGEKIMLAIITKMTLTQVSTWFANARRRLKKEN

>Human|IRX4|Irx|TALE

SGTRRKNATRETTSTLKAWLQEHRKNPYPTKGEKIMLAIITKMTLTQVSTWFANARRRLKKEN

>Zebrafish|irx4a|Irx|TALE

GTRRKNATRETTSTLKAWLQEHRKNPYPTKGEKIMLAIITKMTLTQVSTWFANARRRLKKEN

>Zebrafish|irx4b|Irx|TALE

GTRRKNATRETTSTLKAWLQEHKKNPYPTKGEKIMLAIITKMTLTQVSTWFANARRRLKKEN

>Human|IRX5|Irx|TALE

DPAYRKNATRDATATLKAWLNEHRKNPYPTKGEKIMLAIITKMTLTQVSTWFANARRRLKKEN

>Chicken|IRX5|Irx|TALE

SGTRRKNATRETTSTLKTWLYEHRKNPYPTKGEKIMLAIITKMTLTQVSTWFANARRRLKKEN

>Zebrafish|irx5a|Irx|TALE

DPAYRKNATRDATATLKAWLNEHRKNPYPTKGEKIMLAIITKMTLTQVSTWFANARRRLKKEN

>Zebrafish|irx5b|Irx|TALE

DPAYRKNATRDATATLKAWLNEHRKNPYPTKGEKIMLAIITKMTLTQVSTWFANARRRLKKEN

>Human|IRX6|Irx|TALE

GAGRRKNATRETTSTLKAWLNEHRKNPYPTKGEKIMLAIITKMTLTQVSTWFANARRRLKKEN

>Zebrafish|irx6a|Irx|TALE

TRRKNATRETTSTLKTWLYEHRKNPYPTKGEKIMLAIITKMTLTQVSTWFANARRRLKKEN

>Zebrafish|irx7|Irx|TALE

DPSRVTKVATRESTSALKAWLSEHLKNPYPTKGEKIMLAIVTKMSLTQVSTWFANARRRLKKEN

>Human|ISL1|Isl|LIM

TTRVRTVLNEKQLHTLRTCYAANPRPDALMKEQLVEMTGLSPRVIRVWFQNKRCKDKKRS

>Chicken|ISL1|Isl|LIM

TTRVRTVLNEKQLHTLRTCYAANPRPDALMKEQLVEMTGLSPRVIRVWFQNKRCKDKKRS

>Zebrafish|isl1|Isl|LIM

TTRVRTVLNEKQLHTLRTCYNANPRPDALMKEQLVEMTGLSPRVIRVWFQNKRCKDKKRS

>Zebrafish|isl1l|Isl|LIM

TRVRTVLSETQLCMLQTCYTANPRPDALMKEQLVEMTGLSPRVIRVWFQNKRCKDKKRS

>Human|ISL2|Isl|LIM

TTRVRTVLNEKQLHTLRTCYAANPRPDALMKEQLVEMTGLSPRVIRVWFQNKRCKDKKKS

>Zebrafish|isl2a|Isl|LIM

TTRVRTVLNEKQLHTLRTCYNANPRPDALMKEQLVEMTGLSPRVIRVWFQNKRCKDKKKS

>Zebrafish|isl2b|Isl|LIM

TTRVRTVLNEKQLHTLRTCYNANPRPDALMKEQLVEMTGLSPRVIRVWFQNKRCKDKKRS

>Human|ISX|Isx|PRD

KRRVRTTFTTEQLHELEKIFHFTHYPDVHIRSQLAARINLPEARVQIWFQNQRAKWRKQE

>Chicken|ISX|Isx|PRD

KRRIRTTFTAEQLQELEKIFQVTHYPDVHIRNQLAAKINLPEARVQIWFQNQRAKWRKHE

>Chicken|LASS2|Cers|CERS

VKDKLRPKAQPISILETFYTVNCKNPKEGELISLAKQCDLPVRKVERWFRRRRNTDRPSL

>Zebrafish|lass2|unassigned|Other

VKEKVRVRAAHNPMLEAYFRSTSKSPKQAEVESLVKKTGHTEREIHRWFRRRRNQERPN

>Chicken|LASS3|Cers|CERS

GIKNVRRVKPQPNPVLESYFRECSRHPSQSEIQGLAKKCNCTVHLVEKWFRRRRNLEIPT

>Chicken|LASS4|Cers|CERS

VRDKRRPKAQPSATLEGFYKLLGRTPKEGDLISVAKQSGLPVRTVQTWFRHRRAQDHPRL

>Zebrafish|lass5|Cers|CERS

IQTELSRRAQPNAVLEKVFTSITKSPDSRHSEGLSKQLDWDVRKVQRWFRHRRNQDKPST

>Chicken|LASS6|Cers|CERS

VQANGPQKAQPNAILEKVFTAITKHPDEKRLEGLSKQLDWDVRSIQRWFRQRRNQEKPST

>Chicken|Lbx-l|Lbx|ANTP

RRKSRTAFSNQQLFELERRFVRQKYLSPADRDQLAQRLALSSAQVITWFQNRRAKLKR

>Human|LBX1|Lbx|ANTP

RRKSRTAFTNHQIYELEKRFLYQKYLSPADRDQIAQQLGLTNAQVITWFQNRRAKLKREL

>Zebrafish|lbx1a|Lbx|ANTP

RRKSRTAFTNHQLYELEKRFLHQKYLSPADRDQIAHQLGLTNAQVITWFQNRRAKLKRDL

>Zebrafish|lbx1b|Lbx|ANTP

RRKSRTAFTNHQIYELEKRFLYQKYLSPADRDQIAQQLGLTNAQVITWFQNRRAKLKRDL

>Human|LBX2|Lbx|ANTP

RRKSRTAFTAQQVLELERRFVFQKYLAPSERDGLATRLGLANAQVVTWFQNRRAKLKRDV

>Zebrafish|lbx2|Lbx|ANTP

RRKSRTAFTNHQIYELEKRFLYQKYLSPADRDQIAQQLGLTNAQVITWFQNRRAKLKRDL

>Human|LEUTX|Leutx|PRD

YRRPRTRFLSKQLTALRELLEKTMHPSLATMGKLASKLQLDLSVVKIWFKNQRAKWKRQQ

>Human|LHX1|Lhx1/5|LIM

RRGPRTTIKAKQLETLKAAFAATPKPTRHIREQLAQETGLNMRVIQVWFQNRRSKERRMK

>Chicken|LHX1|Lhx1/5|LIM

RRGPRTTIKAKQLETLKAAFAATPKPTRHIREQLAQETGLNMRVIQVWFQNRRSKERRMK

>Zebrafish|lhx1a|Lhx1/5|LIM

RRGPRTTIKAKQLETLKAAFAATPKPTRHIREQLAQETGLNMRVIQVWFQNRRSKERRMK

>Zebrafish|lhx1b|Lhx1/5|LIM

RRGPRTTIKAKQLETLKAAFAATPKPTRHIREQLAQETGLNMRVIQVWFQNRRSKERRMK

>Human|LHX2|Lhx2/9|LIM

TKRMRTSFKHHQLRTMKSYFAINHNPDAKDLKQLAQKTGLTKRVLQVWFQNARAKFRRNL

>Chicken|LHX2|Lhx2/9|LIM

TKRMRTSFKHHQLRTMKSYFAINHNPDAKDLKQLAQKTGLTKRVLQVWFQNARAKFRRNL

>Zebrafish|lhx2a|Lhx2/9|LIM

SKRMRTSFKHHQLRTMQSFFTHNHNPDAKDLKELAQKTGLTKRVLQVWFQNARAKFRRN

>Zebrafish|lhx2b|Lhx2/9|LIM

TKRMRTSFKHHQLRTMKSYFAINHNPDAKDLKQLAQKTGLTKRVLQVWFQNARAKFRRNL

>Human|LHX3|Lhx3/4|LIM

AKRPRTTITAKQLETLKSAYNTSPKPARHVREQLSSETGLDMRVVQVWFQNRRAKEKRLK

>Chicken|LHX3|Lhx3/4|LIM

AKRPRTTITAKQLETLKNAYNNSPKPARHVREQLSSETGLDMRVVQVWFQNRRAKEKRLK

>Zebrafish|lhx3|Lhx3/4|LIM

AKRPRTTITAKQLETLKNAYNNSPKPARHVREQLSTETGLDMRVVQVWFQNRRAKEKRLK

>Zebrafish|Lhx3/4-l|Lhx3/4|LIM

AKRPRTTITAKQLETLKSAYKNSPKPARHVREQLSSETGLDMRVVQVWFQNRRAKEKRLK

>Human|LHX4|Lhx3/4|LIM

AKRPRTTITAKQLETLKNAYKNSPKPARHVREQLSSETGLDMRVVQVWFQNRRAKEKRLK

>Chicken|Lhx4-l|Lhx3/4|LIM

KRPRTTITAKQLETLKNAYKNSPKPARHVREQLSSETGLDMRVVQVWFQNRRAKEKR

>Human|LHX5|Lhx1/5|LIM

RRGPRTTIKAKQLETLKAAFAATPKPTRHIREQLAQETGLNMRVIQVWFQNRRSKERRMK

>Chicken|LHX5|Lhx1/5|LIM

RRGPRTTIKAKQLETLKAAFAATPKPTRHIREQLAQETGLNMRVIQVWFQNRRSKERRMK

>Zebrafish|lhx5|Lhx1/5|LIM

RRGPRTTIKAKQLETLKAAFVATPKPTRHIREQLAQETGLNMRVIQVWFQNRRSKERRMK

>Human|LHX6|Lhx6/8|LIM

AKRARTSFTAEQLQVMQAQFAQDNNPDAQTLQKLADMTGLSRRVIQVWFQNCRARHKKHT

>Chicken|LHX6|Lhx6/8|LIM

AKRARTSFTAEQLQVMQAQFAQDNNPDAQTLQKLADMTGLSRRVIQVWFQNCRARHKKHT

>Zebrafish|lhx6|Lhx6/8|LIM

AKRARTSFTAEQLQVMQAQFAQDNNPDAQTLQKLADMTGLSRRVIQVWFQNCRARHKKHT

>Zebrafish|lhx6-l|Lhx6/8|LIM

SKRPRTSFTSEQIQIMQTHFIRDKNPDAATLQRLADTTGLSRRVIQVWFQNCRARQKR

>Zebrafish|Lhx6/8-l|Lhx6/8|LIM

SKRARTSFTADQLQVMQAQFVQDNNPDAQMLQSLAEQTGLSRRVIQVWFQNCRARHKKHV

>Human|LHX8|Lhx6/8|LIM

AKRARTSFTADQLQVMQAQFAQDNNPDAQTLQKLAERTGLSRRVIQVWFQNCRARHKKHV

>Chicken|LHX8|Lhx6/8|LIM

AKRARTSFTADQLQVMXAQFAQDNNPDAQTLQKLAERTGLSRRVIQVWFQNCRARHKKHV

>Zebrafish|lhx8a|Lhx6/8|LIM

AKRARTSFTADQLQVMQAQFAQDNNPDAQTLQKLAERTGLSRRVIQVWFQNCRARHKKHV

>Human|LHX9|Lhx2/9|LIM

TKRMRTSFKHHQLRTMKSYFAINHNPDAKDLKQLAQKTGLTKRVLQVWFQNARAKFRRNL

>Chicken|LHX9|Lhx2/9|LIM

TKRMATSFKHHQLRTMKSYFAINHNPDAKDLKQLAQKTGLTKRVLQVWFQNARAKFRRNL

>Zebrafish|lhx9|Lhx2/9|LIM

TKRMRTSFKHHQLRTMKSYFAINHNPDAKDLKQLAQKTGLTKRVLQ

>Chicken|Lmx-l|Lmx|LIM

RETLAAETGLTVRVVQVWFQNQRAK

>Zebrafish|Lmx-l|Lmx|LIM

KRPRTILTTQQRRAFKASFEVSSKPCRKVRETLAAETGLTVRVVQVWFQNQRAKMKKIA

>Human|LMX1A|Lmx|LIM

PKRPRTILTTQQRRAFKASFEVSSKPCRKVRETLAAETGLSVRVVQVWFQNQRAKMKKLA

>Human|LMX1B|Lmx|LIM

PKRPRTILTTQQRRAFKASFEVSSKPCRKVRETLAAETGLSVRVVQVWFQNQRAKMKKLA

>Chicken|LMX1B|Lmx|LIM

PKRPRTILTTQQRRAFKASFEVSSKPCRKVRETLAAETGLSVRVVQVWFQNQRAKMKKLA

>Zebrafish|lmx1b.1|Lmx|LIM

PKRPRTILTTQQRRAFKASFEVSSKPCRKVRETLAAETGLSVRVVQVWFQNQRAKMKKLA

>Zebrafish|lmx1b.2|Lmx|LIM

PKRPRTILTTQQRRAFKASFEVSSKPCRKVRETLAAETGLSVRVVQVWFQNQRAKMKKLA

>Zebrafish|LOC100000073|Onecut|CUT

KKSRLVFTDLQRRTLLAIFKENKRPSKEMQMTISQQLGLELSTVSNFFMNARRR

>Zebrafish|LOC100001518|Pou6|POU

KRKRRTSFTPQALEILNSHFEKNTHPSGQEMTEIAEKLNYDREVVRVWFCNKRQALKNT

>Zebrafish|LOC100008066|Tlx|ANTP

QICELEKRFHRQKYLASAERAALAKSLKMTDAQVKTWFQNRRTKWR

>Zebrafish|LOC100330155|unassigned|Other

GVKDPVRLKVTPSPSLEAFYTKNSRQPTQIEISGLVKQCGLTHRQVETWFRNRRNLDRPS

>Zebrafish|LOC100330956|Mix|PRD

RRKRTNFTQQQIDVLEKVYLDTKYPDIYLREKLEALTGLPESRIQVWFQNRRAKSRRQ

>Zebrafish|LOC100332856|unassigned|Other

RRPRTAFSSEQISSLERVFKRNAYLGAQDKAELCRTLKLTDKQIRNWFQNRRMKLKRTV

>Zebrafish|LOC100333298|Nk5/Hmx|ANTP

KKKTRTVFSRAQVFQLESTFDLKRYLSSSERAGLAASLHLTETQVKIWFQNRRNKWKRQI

>Human|LOC100506764|HD1|Dux|PRD

GRRRRLVWTPSQSEVLRACFERNPYPGIATRERLAQAIGIPEPRVQIWFQNERSRQLRQH

>Human|LOC100506764|HD2|Dux|PRD

GRRKRTAVTGSQTALLLRAFEKDRFPGIAAREELARETGLPESRIQIWFQNRRARHPGQG

>Chicken|LOC415513|Cers|CERS

SEIQGLAKKCNCTVHLVEKWFRRRRNLEIPT

>Chicken|LOC419032|Nobox|PRD

KKTRTFYSAEQLEELEKVFQEDRYPDNEKRREIAAVIGVTPQRIMVWFQNRRAKWRKT

>Chicken|LOC428131|Tgif|TALE

SQICNWFINARRRL

>Chicken|LOC428735|Pou4|POU

KKRKRTSIAAPEKRSLEAYFALQPRPSSEKIAAIAEKLDLKKNVVRVWFCNQRQKQKRMK

>Chicken|LOC429994|Gsx|ANTP

GKRMRTAFTSTQLLELEREFSSNMYLSRLRRIEIATYLNLSEKQVKIWFQNRRVKHKKEG

>Zebrafish|LOC556453|HD1|Zfhx|ZF

KRPRTRITDDQLKILRAHFDINNSPNEEQIQEMADKSGLPQKVIKHWFRNTLFKERQ

>Zebrafish|LOC556453|HD2|Zfhx|ZF

RTRFTDYQLRVLQDFFDTNAYPKDDEIEQLSTVLNLPTRVIVVWFQNARQKARK

>Zebrafish|LOC556453|HD3|Zfhx|ZF

KRLRTTITPEQLEILYDKYLLDSNPTRKMLDHIAHEVGLKKRVVQVWFQNTRARERK

>Zebrafish|LOC556453|HD4|Zfhx|ZF

HKRFRTQMSNLQLKVLKACFSDYRTPTMQECEMLGNEIGLPKRVVQVWFQNARAKEKKFK

>Zebrafish|LOC556898|Meox|ANTP

PRKERTAFTKEQIRELEAEFAHHNYLTRLRRYEIAVNLDLTERQVKVWFQNRRMKWKRVK

>Zebrafish|LOC558522|Tshz|ZF

RKGRQSHWKPQHLLILQAQFTSCLRQTADGKYVISDLSSQERMVISHITGLSMTTISHWLANVKYQLRRTG

>Zebrafish|LOC566969|Meox|ANTP

PRKERTAFTKEQIRELESEFAHHNYLTRLRRYEIAVNLDLTERQVKVWFQNRRMKWKRVK

>Zebrafish|LOC571757|Satb|CUT

KSRQPNRISMEALGILQSFIQDVGLYPDEEAIHTLSAQLDLPKHTIIKFFQNQRFYINH

>Human|LOC647589|LOC647589|Other

KSRNFPREVREKLHNFAVGVNTNPSKAERENLALETSLTPEQVYNWFANYRRRQRALPQH

>Chicken|LOC769839|Mix|PRD

RRKRTSFSKAQLELLVRTFEKQPYPGIALREQLSGLTDIPESRIQV

>Chicken|LOC770725|HD1|Lmx|LIM

PKRPRTILTTQQRRAFKASFEVSSKPCRKQR

>Chicken|LOC770725|HD2|Lmx|LIM

PKRPRTILTTQQRRAFKASFEVSSKPCRKVRETLAAETGLSVRVVQVWFQNQRAKMKKLA

>Chicken|LOC771564|Otp|PRD

QKRHRTRFTPAQLNELERRFAKTHYPDIFMREELALRIGLTESRVQV

>Chicken|LOC771682|Hox9-13(15)|ANTP

GRKKRVPYSKGQLKELEKEYASSKFITRDKRRKISAATNLTERQITIWFQNRRVKEKKVV

>Zebrafish|LOC797948|HD1|Hdx|POU

RSVFTAEQQRILERYYENGMTNQSKSCFQLILQCAQETKLDFSVVRTWVGNKRRKL

>Zebrafish|LOC797948|HD2|Hdx|POU

NSRKRTLQDRTQFSDRDLYTLKRYWDNGMTSLGSLCKEKIAAAAIQLNVDTEIIKTWIGNRRRKYRLMG

>Human|MEIS1|Meis|TALE

RHKKRGIFPKVATNIMRAWLFQHLTHPYPSEEQKKQLAQDTGLTILQVNNWFINARRRIVQPM

>Chicken|MEIS1|Meis|TALE

RGIFPKVATNIMRAWLFQHLTHPYPSEEQKKQLAQDTGLTILQVNNWFINARRTLFKPM

>Zebrafish|meis1|Meis|TALE

RNKKRGIFPKVATNIMRAWLFQHLTHPYPSEEQKKQLAQDTGLTILQVNNWFINARRRIVQPM

>Human|MEIS2|Meis|TALE

RQKKRGIFPKVATNIMRAWLFQHLTHPYPSEEQKKQLAQDTGLTILQVNNWFINARRRIVQPM

>Chicken|MEIS2|Meis|TALE

RQKKRGIFPKVATNIMRAWLFQHLTHPYPSEEQKKQLAQDTGLTILQVNNWFINARRRIVQPM

>Zebrafish|meis2.1|Meis|TALE

RQKKRGIFPKVATNIMRAWLFQHLTHPYPSEEQKKQLAQDTGLTNLQVNNWFINARRRIVQPM

>Zebrafish|meis2.2|Meis|TALE

RQKKRGIFPKVATNIMRAWLFQHLTHPYPSEEQKKQLAQDTGLTILQVNNWFINARRRIVQPM

>Human|MEIS3|Meis|TALE

RNKKRGIFPKVATNIMRAWLFQHLSHPYPSEEQKKQLAQDTGLTILQVNNWFINARRRIVQPM

>Zebrafish|meis3|Meis|TALE

NKKRGIFPKVATNIMRAWLFQHLSHPYPSEEQKKQLSQDTGLTILQVNNWFINARRRIVQPM

>Zebrafish|meis4.1a|Meis|TALE

NKKRGIFPKVATNIMRAWLFQHLTHPYPSEEQKRQLSQDTGLTILQVNNWFINARRRIVQPM

>Human|MEOX1|Meox|ANTP

ARKERTAFTKEQLRELEAEFAHHNYLTRLRRYEIAVNLDLSERQVKVWFQNRRMKWKRVK

>Chicken|MEOX1|Meox|ANTP

RKERTAFTKEQLRELEAEFAHHNYLTRLRRYEIAVNLDLTERQVKVWFQNRRMKWKRVK

>Zebrafish|meox1|Meox|ANTP

ARKERTAFTKEQLRELEAEFTHHNYLTRLRRYEIAVNLDLTERQVKVWFQNRRMKWKRVK

>Human|MEOX2|Meox|ANTP

PRKERTAFTKEQIRELEAEFAHHNYLTRLRRYEIAVNLDLTERQVKVWFQNRRMKWKRVK

>Chicken|MEOX2|Meox|ANTP

PRKERTAFTKEQIRELEAEFAHHNYLTRLRRYEIAVNLDLTERQVKVWFQNRRMKWKRVK

>Human|MIXL1|Mix|PRD

QRRKRTSFSAEQLQLLELVFRRTRYPDIHLRERLAALTLLPESRIQVWFQNRRAKSRRQS

>Chicken|MIXL1|Mix|PRD

RRKRTSFTAAQLETLELVFQDTMYPDIYLRERLADATQIPESRIQVWFQNRRAK

>Human|MKX|Mkx|TALE

KVRHKRQALQDMARPLKQWLYKHRDNPYPTKTEKILLALGSQMTLVQVSNWFANARRRLKNTV

>Chicken|MKX|Mkx|TALE

KVRHKRQALQDMARPLKQWLYKHRDNPYPTKTEKILLALGSQMTLVQVSNWFANARRRLKNTV

>Zebrafish|mkxa|Mkx|TALE

KVRHKRQALQDMARPLKQWLYKHRDNPYPTKTEKILLALGSQMTLVQVSNWFANARRRLKNTV

>Chicken|MNR2|Mnx|ANTP

RRPRTAFTSQQLLELENQFKLNKYLSRPKRFEVATSLMLTETQVKIWFQNRRMKWKRSR

>Human|MNX1|Mnx|ANTP

CRRPRTAFTSQQLLELEHQFKFNKYLSRPKRFEVATSLMLTETQVKIWFQNRRMKWKRSK

>Chicken|MNX1|Mnx|ANTP

CRRPRTAFTSQQLLELEHQFKLNKYLSRPKRFEVATSLMLTETQVKIWFQNRRMKWKR

>Zebrafish|mnx1|Mnx|ANTP

CRRPRTAFTSQQLLELEHQFKLNKYLSRPKRFEVATSLMLTETQVKIWFQNRRMKWKRSK

>Human|MSX1|Msx|ANTP

NRKPRTPFTTAQLLALERKFRQKQYLSIAERAEFSSSLSLTETQVKIWFQNRRAKAKRLQ

>Chicken|MSX1|Msx|ANTP

NRKPRTPFTTAQLLALERKFRQKQYLSIAERAEFSSSLSLTETQVKIWFQNRRAKAKRLQ

>Human|MSX2|Msx|ANTP

NRKPRTPFTTSQLLALERKFRQKQYLSIAERAEFSSSLNLTETQVKIWFQNRRAKAKRLQ

>Chicken|MSX2|Msx|ANTP

NRKPRTPFTTSQLLALERKFRQKQYLSIAERAEFSSSLNLTETQVKIWFQNRRAKAKRLQ

>Zebrafish|msxa|Msx|ANTP

NRKPRTPFTTAQLLALERKFRQKQYLSIAERAEFSSSLSLTETQVKIWFQNRRAKAKRLQ

>Zebrafish|msxb|Msx|ANTP

NRKPRTPFSTSQLLSLERKFRQKQYLSIAERAEFSNSLNLTETQVKIWFQNRRAKAKRLQ

>Zebrafish|msxc|Msx|ANTP

NRKPRTPFTTSQLLALERKFRQKQYLSIAERAEFSNSLNLTETQVKIWFQNRR

>Zebrafish|msxd|Msx|ANTP

NRKPRTPFTTSQLLALERKFRQKQYLSIAERAEFSSSLTLTETQVKIWFQNRRAKAKRLQ

>Zebrafish|msxe|Msx|ANTP

NRKPRTPFSTAQLLALERKFRQKQYLSIAERAEFSSSLSLTETQVKIWFQNRRAKAKRLQ

>Zebrafish|mxtx1|unassigned|Other

RRKRTSFSKEHVELLRATFETDPYPGISLRESLSQTTGLPESRIQVWFQNRRAR

>Zebrafish|mxtx2|unassigned|Other

RRKRTSFTKEHLELLKMAFNVDPYPGISVRESLSQATGLPESRIQVWFQNKRAR

>Human|NANOG|Nanog|ANTP

KQKTRTVFSSTQLCVLNDRFQRQKYLSLQQMQELSNILNLSYKQVKTWFQNQRMKSKRWQ

>Human|NANOGNB|NANOGNB|Other

QYPEKRLVSKSLMHTLWAKFKLNRCPTIQESLSLSFEFDMTHKQISQWFCKTRKKYNKEM

>Human|NKX1-1|Nk1|ANTP

PRRARTAFTYEQLVALENKFKATRYLSVCERLNLALSLSLTETQVKIWFQNRRTKWKKQN

>Chicken|NKX1-1|Nk1|ANTP

PRRARTAFTYEQLVALENKFKSTRYLSVCERLNLALSLSLTETQVKIWFQNRRTKWKKQN

>Human|NKX1-2|Nk1|ANTP

PRRARTAFTYEQLVALENKFRATRYLSVCERLNLALSLSLTETQVKIWFQNRRTKWKKQN

>Chicken|NKX1-2|Nk1|ANTP

PRRARTAFTYEQLVALENKFRATRYLSVCERLNLALSLSLTETQVKIWFQNRRTKWKKQH

>Zebrafish|nkx1.2la|Nk1|ANTP

PRRARTAFTYEQLVALENKLRATRYLSVCERLNLALSLSLTETQVKIWFQNRRTKWKKQN

>Zebrafish|nkx1.2lb|Nk1|ANTP

PRRARTAFTYEQLVALENKFKSTRYLSVCERLNLALSLSLTETQVKIWFQNRRTKWKKQN

>Human|NKX2-1|Nk2.1|ANTP

RRKRRVLFSQAQVYELERRFKQQKYLSAPEREHLASMIHLTPTQVKIWFQNHRYKMKRQA

>Chicken|NKX2-1|Nk2.1|ANTP

RRKRRVLFSQAQVYELERRFKQQKYLSAPEREHLASMIHLTPTQVKIWFQNHRYKMKRQA

>Human|NKX2-2|Nk2.2|ANTP

KRKRRVLFSKAQTYELERRFRQQRYLSAPEREHLASLIRLTPTQVKIWFQNHRYKMKRAR

>Human|NKX2-3|Nk4|ANTP

RRKPRVLFSQAQVFELERRFKQQRYLSAPEREHLASSLKLTSTQVKIWFQNRRYKCKRQR

>Human|NKX2-4|Nk2.1|ANTP

RRKRRVLFSQAQVYELERRFKQQKYLSAPEREHLASMIHLTPTQVKIWFQNHRYKMKRQA

>Human|NKX2-5|Nk4|ANTP

RRKPRVLFSQAQVYELERRFKQQRYLSAPERDQLASVLKLTSTQVKIWFQNRRYKCKRQR

>Chicken|NKX2-5|Nk4|ANTP

RRKPRVLFSQAQVYELERRFKQQKYLSAPERDHLANVLKLTSTQVKIWFQNRRYKCKRQR

>Human|NKX2-6|Nk4|ANTP

RRKPRVLFSQAQVLALERRFKQQRYLSAPEREHLASALQLTSTQVKIWFQNRRYKCKRQR

>Chicken|NKX2-6|Nk4|ANTP

RRKPRVLFSQTQVLELERRFKQQKYLSALEREHLANVLQLTSTQVKIWFQNRRYKCKRQR

>Human|NKX2-8|Nk2.2|ANTP

RKKRRVLFSKAQTLELERRFRQQRYLSAPEREQLASLLRLTPTQVKIWFQNHRYKLKRAR

>Chicken|Nkx2-9|Nk2.2|ANTP

RKRRVLFSKAQTLELERRFRQQRYLSAPEREQLARLLRLTPTQVKIWFQNHRYKLKRGR

>Zebrafish|nkx2.1a|Nk2.1|ANTP

RRKRRVLFSQAQVYELERRFKQQKYLSAPEREHLASMIHLTPTQVKIWFQNHRYKMKRQA

>Zebrafish|nkx2.1b|Nk2.1|ANTP

RRKRRVLFSQAQVYELERRFKQQKYLSAPEREHLASMIHLTPTQVKIWFQNHRYKMKRQA

>Zebrafish|nkx2.1c|Nk2.1|ANTP

RRKRRVLFSQAQVCELERRFKQQKYLSAPEREHLASMIHLTPTQVKIWFQNHRYKMKRQA

>Zebrafish|nkx2.2a|Nk2.2|ANTP

KRKRRVLFSKAQTYELERRFRQQRYLSAPEREHLASLIRLTPTQVKIWFQNHRYKMKRAR

>Zebrafish|nkx2.2b|Nk2.2|ANTP

KRKRRILFSKTQTFELERRFRQQRYLSAPEREHLAKLLHLTPTQVKIWFQNHRYKVKRAR

>Zebrafish|nkx2.3|Nk4|ANTP

RRKPRVLFSQAQVFELERRFKQQRYLSAPEREHLASTLKLTSTQVKIWFQNRRYKCKRQR

>Zebrafish|nkx2.5|Nk4|ANTP

RRKPRVLFSQAQVYELERRFKQQKYLSAPERDHLANVLKLTSTQVKIWFQNRRYKCKRQR

>Zebrafish|nkx2.7|Nk4|ANTP

RRKPRVLFSQTQVFELERRFKQQRYLSAPERDHLALALKLTSTQVKIWFQNRRYKCKRQR

>Zebrafish|nkx2.9|Nk2.2|ANTP

RKKRRVLFSKAQTYELERRFRQQRYLSAPEREQLAHLLRLTPTQVKIWFQNHRYKMKRAR

>Human|NKX3-1|Nk3|ANTP

QKRSRAAFSHTQVIELERKFSHQKYLSAPERAHLAKNLKLTETQVKIWFQNRRYKTKRKQ

>Zebrafish|nkx3-1|Nk3|ANTP

KKRSRAAFTHLQVLELEKKFSRQRYLSAPERTHLASALHLTETQVKIWFQNRRYKTKRRQ

>Human|NKX3-2|Nk3|ANTP

KKRSRAAFSHAQVFELERRFNHQRYLSGPERADLAASLKLTETQVKIWFQNRRYKTKRRQ

>Zebrafish|nkx3.2|Nk3|ANTP

KKRSRAAFSHAQVFELERRFNHQRYLSGPERADLAASLKLTETQVKIWFQNRRYKTKRRQ

>Zebrafish|nkx3.3|Nk3|ANTP

KKRSRAAFSHAQVYELARRFNLQRYLSGPERADLAGALKLTETQVKIWFQNRRYKTKRRQ

>Human|NKX6-1|Nk6|ANTP

RKHTRPTFSGQQIFALEKTFEQTKYLAGPERARLAYSLGMTESQVKVWFQNRRTKWRKKH

>Human|NKX6-2|Nk6|ANTP

KKHSRPTFSGQQIFALEKTFEQTKYLAGPERARLAYSLGMTESQVKVWFQNRRTKWRKRH

>Chicken|NKX6-2|Nk6|ANTP

KKHSRPTFSGQQIFALEKTFEQTKYLAGPERARLAYSLGMTESQVKVWFQNRRTKWRKRH

>Human|NKX6-3|Nk6|ANTP

KKHTRPTFTGHQIFALEKTFEQTKYLAGPERARLAYSLGMTESQVKVWFQNRRTKWRKKS

>Chicken|NKX6-3|Nk6|ANTP

KKHTRPTFTGHQIFALEKTFEQTKYLAGPERARLAYSLGMTESQVKVWFQNRRTKWRKKS

>Zebrafish|nkx6.1|Nk6|ANTP

RKHTRPTFSGQQIFALEKTFEQTKYLAGPERARLAYSLGMTESQVKVWFQNRRTKWRKRH

>Zebrafish|nkx6.2|Nk6|ANTP

KKHSRPTFSGQQIFALEKTFEQTKYLAGPERARLAYSLGMTESQVKVWFQNRRTKWRKRH

>Zebrafish|nkx6.3|Nk6|ANTP

KKHTRPTFSGHQIFALEKTFEQTKYLAGPERARLAYSLGMTESQVKVWFQNRRTKWRKKS

>Human|NOBOX|Nobox|PRD

RKKTRTLYRSDQLEELEKIFQEDHYPDSDKRREIAQTVGVTPQRIMVWFQNRRAKWRKME

>Human|NOTO|Noto|ANTP

QKRVRTMFNLEQLEELEKVFAKQHNLVGKKRAQLAARLKLTENQVRVWFQNRRVKYQKQQ

>Zebrafish|og9x|Mix|PRD

RKRKRTIFSRAQLSELERAFMITPYPDITLRERLAALTLLPESKIQVWFQNRRARSMK

>Zebrafish|Onecut-l|Onecut|CUT

PKKQRLVFTDLQRRTLIAIFKENKRPSKEMQITISQQLGLELSTVSNFFMNARRRCVDRW

>Human|ONECUT1|Onecut|CUT

PKKPRLVFTDVQRRTLHAIFKENKRPSKELQITISQQLGLELSTVSNFFMNARRRSLDKW

>Zebrafish|onecut1|Onecut|CUT

KKPRLVFTDVQRRTLHAIFKENKRPSKELQITISQQLGLELATVSNFFMNARRR

>Human|ONECUT2|Onecut|CUT

QKKSRLVFTDLQRRTLFAIFKENKRPSKEMQITISQQLGLELTTVSNFFMNARRRSLEKW

>Human|ONECUT3|Onecut|CUT

PKKQRLVFTDLQRRTLIAIFKENKRPSKEMQVTISQQLGLELNTVSNFFMNARRRCMNRW

>Zebrafish|onecut3|Onecut|CUT

PKKQRLVFTDLQRRTLIAIFKENKRPSKEMQLTISQQLGLELSTVSNFFMNARRRCVDRW

>Zebrafish|onecutl|Onecut|CUT

PKRTRLVFTDLQRRTLMAIFRENHRPTKDLQITISQQLGLELSTVSNFFMNARRRNLNRW

>Human|OTP|Otp|PRD

QKRHRTRFTPAQLNELERSFAKTHYPDIFMREELALRIGLTESRVQVWFQNRRAKWKKRK

>Zebrafish|otpa|Otp|PRD

QKRHRTRFTPAQLNELERSFAKTHYPDIFMREELALRIGLTESRVQVWFQNRRAKWKKRK

>Zebrafish|otpb|Otp|PRD

QKRHRTRFTPAQLNELERSFAKTHYPDIFMREELALRIGLTESRVQVWFQNRRAKWKKRK

>Human|OTX1|Otx|PRD

QRRERTTFTRSQLDVLEALFAKTRYPDIFMREEVALKINLPESRVQVWFKNRRAKCRQQQ

>Zebrafish|otx1a|Otx|PRD

QRRERTTFTRTQLDILESLFAKTRYPDIFMREEVALKINLPESRVQVVWFKNRRAKCRQQQ

>Zebrafish|otx1b|Otx|PRD

QRRERTTFTRSQLDILEALFAKTRYPDIFMREEVALKINLPESRVQVWFKNRRAKCRQQQ

>Human|OTX2|Otx|PRD

QRRERTTFTRAQLDVLEALFAKTRYPDIFMREEVALKINLPESRVQVWFKNRRAKCRQQQ

>Zebrafish|otx2|Otx|PRD

QRRERTTFTRAQLDVLEALFAKTRYPDIFMREEVALKINLPESRVQVWFKNRRAKCRQQQ

>Zebrafish|otx5|Otx|PRD

QRRERTTFTRAQLDVLEALFSKTRYPDIFMREEVALKINLPESRVQVWFKNRRAKCRQQQ

>Human|PAX2|Pax2/5/8|PRD

KHLRADTFTQQQLEALDRVFERPSYPDVFQASE

>Zebrafish|pax2b|Pax2/5/8|PRD

KHLRADAFTQQQLEALDRVFERPAFPDVF

>Human|PAX3|Pax3/7|PRD

QRRSRTTFTAEQLEELERAFERTHYPDIYTREELAQRAKLTEARVQVWFSNRRARWRKQA

>Chicken|PAX3|Pax3/7|PRD

QRRSRTTFTAEQLEELERAFERTHYPDIYTREELAQRAKLTEARVQVWFSNRRARWRKQA

>Zebrafish|pax3a|Pax3/7|PRD

QRRSRTTFTAEQLEELERAFERTHYPDIYTREELAQRAKLTEARVQVWFSNRRARWRKQA

>Zebrafish|pax3b|Pax3/7|PRD

QRRSRTTFTADQLEELERAFERTHYPDIYTREELAQRAKLTEARVQVWFSNRRARWRKQA

>Human|PAX4|Pax4/6|PRD

GHRNRTIFSPSQAEALEKEFQRGQYPDSVARGKLATATSLPEDTVRVWFSNRRAKWRRQE

>Zebrafish|pax4|Pax4/6|PRD

HRSRTAFTADQSGRLEKEFTCGLYPDLLTREKLAEETNLSQDTIKVWFSNRRARMRRE

>Human|PAX5|Pax2/5/8|PRD

KQMRGDLFTQQQLEVLDRVFERQHYSDIFTTTE

>Human|PAX6|Pax4/6|PRD

LQRNRTSFTQEQIEALEKEFERTHYPDVFARERLAAKIDLPEARIQVWFSNRRAKWRREE

>Chicken|PAX6|Pax4/6|PRD

LQRNRTSFTQEQIEALEKEFERTHYPDVFARERLAAKIDLPEARIQVWFSNRRAKWRREE

>Zebrafish|Pax6-l|Pax4/6|PRD

LQRNRTSFTQEQIDALEKEFERTHYPDVFARERLAAKIDLPEARIQVWFSNRRAKWRREE

>Zebrafish|pax6a|Pax4/6|PRD

LQRNRTSFTQEQIEALEKEFERTHYPDVFARERLAAKIDLPEARIQVWFSNRRAKWRREE

>Zebrafish|pax6b|Pax4/6|PRD

LQRNRTSFTQEQIEALEKEFERTHYPDVFARERLAAKIDLPEARIQVWFSNRRAKWRREE

>Human|PAX7|Pax3/7|PRD

QRRSRTTFTAEQLEELEKAFERTHYPDIYTREELAQRTKLTEARVQVWFSNRRARWRKQA

>Chicken|PAX7|Pax3/7|PRD

QRRSRTTFTAEQLEELEKAFERTHYPDIYTREELAQRTKLTEARVQVWFSNRRARWRKQA

>Zebrafish|pax7a|Pax3/7|PRD

QRRSRTTFTAEQLEELEKAFERTHYPDIYTREELAQRTKLTEARVQVWFSNRRARWRKQ

>Zebrafish|pax7b|Pax3/7|PRD

QRRSRTTFTAEQLEELEKAFERTHYPDIYTREELAQRTKLTEARVQVWFSNRRARWRKQA

>Human|PAX8|Pax2/5/8|PRD

KHLRTDAFSQHHLEPLECPFERQHYPEAYASPS

>Chicken|Pbx-l|Pbx|TALE

RRKRRNFNKQATEILNEYFYSHLSNPYPSEEAKEELAKKCGITVSQV

>Zebrafish|Pbx-l|Pbx|TALE

ARRKRRNFNKQATEILNEYFYSHLSNPYPSEEAKEELAKKCSITVSQVSNWFGNKRIRYKKNI

>Human|PBX1|Pbx|TALE

ARRKRRNFNKQATEILNEYFYSHLSNPYPSEEAKEELAKKCGITVSQVSNWFGNKRIRYKKNI

>Chicken|PBX1|Pbx|TALE

ARRKRRNFNKQATEILNEYFYSHLSNPYPSEEAKEELAKKCGITVSQVSNWFGNKRIRYKKNI

>Zebrafish|pbx1a|Pbx|TALE

ARRKRRNFNKQATEILNEYFYSHLSNPYPSEEAKEELAKKCSITVSQVSNWFGNKRIRYKKNI

>Human|PBX2|Pbx|TALE

ARRKRRNFSKQATEVLNEYFYSHLSNPYPSEEAKEELAKKCGITVSQVSNWFGNKRIRYKKNI

>Zebrafish|pbx2|Pbx|TALE

ARRKRRNFSKQATEVLNEYFYSHLSNPYPSEEAKEELAKQCSITVSQVSNWFGNKRIRYKKNI

>Chicken|Pbx2-l|Pbx|TALE

RRKRRNFSKQATEVLNEYFYSHLSNPYPSEEAKEELAKKGGITVSQV

>Human|PBX3|Pbx|TALE

ARRKRRNFSKQATEILNEYFYSHLSNPYPSEEAKEELAKKCSITVSQVSNWFGNKRIRYKKNI

>Chicken|PBX3|Pbx|TALE

ARRKRRNFSKQATEILNEYFYSHLSNPYPSEEAKEELAKKCSITVSQVSNWFGNKRIRYKKNI

>Zebrafish|pbx3a|Pbx|TALE

ARRKRRNFSKQATEILNEYFYSHLSNPYPSEEAKEELAKKCAITVSQVSNWFGNKRIRYKKNI

>Zebrafish|pbx3b|Pbx|TALE

ARRKRRNFSKQATEILNEYFYSHLSNPYPSEEAKEELAKKCSITVSQGVGTTITVAQVSNWFGNKRIRYKKNI

>Human|PBX4|Pbx|TALE

ARRKRRNFSKQATEVLNEYFYSHLNNPYPSEEAKEELARKGGLTISQVSNWFGNKRIRYKKNM

>Zebrafish|pbx4|Pbx|TALE

ARRKRRNFNKQATEVLNEYFYSHLSNPYPSEEAKEELAKKCGITVSQVSNWFGNKRIRYKKNI

>Human|PDX1|Pdx|ANTP

NKRTRTAYTRAQLLELEKEFLFNKYISRPRRVELAVMLNLTERHIKIWFQNRRMKWKKEE

>Chicken|PDX1|Pdx|ANTP

NKRTRTAYTRAQLLELEKEFLFNKYISRPRRVELAVMLNLTERHIKIWFQNRRMKWKKEE

>Zebrafish|pdx1|Pdx|ANTP

NKRTRTAYTRAQLLELEKEFLFNKYISRPRRVELALTLSLTERHIKIWFQNRRMKWKKEE

>Zebrafish|Phox-l|Phox|PRD

QRRVRTIFTSAQLKALERAFAHTQYPDIYTREELVQEIQLTEARVQVWFQNRRAKFRKQE

>Human|PHOX2A|Phox|PRD

QRRIRTTFTSAQLKELERVFAETHYPDIYTREELALKIDLTEARVQVWFQNRRAKFRKQE

>Zebrafish|phox2a|Phox|PRD

QRRIRTTFTSSQLKELERVFAETHYPDIYTREELALKIDLTEARVQVWFQNRRAKFRKQE

>Human|PHOX2B|Phox|PRD

QRRIRTTFTSAQLKELERVFAETHYPDIYTREELALKIDLTEARVQVWFQNRRAKFRKQE

>Chicken|PHOX2B|Phox|PRD

QRRIRTTFTSAQLKELERVFAETHYPDIYTREELALKIDLTEARVQVWFQN

>Zebrafish|phox2b|Phox|PRD

QRRIRTTFTSAQLKELERVFAETHYPDIYTREELALKIDLTEARVQVWFQNRRAKFRKQE

>Zebrafish|phox2b-l|Phox|PRD

QRRIRTTFTSAQLKELERVFAETHYPDIYTREELALKIDLTEARVQVWFQNRRAKFRKQE

>Human|PITX1|Pitx|PRD

QRRQRTHFTSQQLQELEATFQRNRYPDMSMREEIAVWTNLTEPRVRVWFKNRRAKWRKRE

>Chicken|PITX1|Pitx|PRD

QRRQRTHFTSQQLQELEATFQRNRYPDMSMREEIAVWTNLTEPRVRVWFKNRRAKWRKRE

>Zebrafish|pitx1|Pitx|PRD

QRRQRTHFTSQQLQELEATFQRNRYPDMSTREEIAVWTNLTEARVRVWFKNRRAKWRKRE

>Human|PITX2|Pitx|PRD

QRRQRTHFTSQQLQELEATFQRNRYPDMSTREEIAVWTNLTEARVRVWFKNRRAKWRKRE

>Chicken|PITX2|Pitx|PRD

QRRQRTHFTSQQLQELEATFQRNRYPDMSTREEIAVWTNLTEARVRVWFKNRRAKWRKRE

>Zebrafish|pitx2|Pitx|PRD

QRRQRTHFTSQQLQELEATFQRNRYPDMSTREEIAVWTNLTEARVRVWFKNRRAKWRKRE

>Human|PITX3|Pitx|PRD

QRRQRTHFTSQQLQELEATFQRNRYPDMSTREEIAVWTNLTEARVRVWFKNRRAKWRKRE

>Chicken|PITX3|Pitx|PRD

QRRQRTHFTSQQLQELEATFQRNRYPDMSTREEIAVWTNLTEARVRVWFKNRRAKWRKRE

>Zebrafish|pitx3|Pitx|PRD

QRRQRTHFTSQQLQELEATFQRNRYPDMSTREEIAVWTNLTEARVRVWFKNRRAKWRKRE

>Human|PKNOX1|Pknox|TALE

SKNKRGVLPKHATNVMRSWLFQHIGHPYPTEDEKKQIAAQTNLTLLQVNNWFINARRRILQPM

>Chicken|PKNOX1|Pknox|TALE

SKNKRGVLPKHATNVMRSWLFQHIGHPYPTEDEKKQIAAQTNLTLLQVNNWFINARRRILQPM

>Zebrafish|pknox1.1|Pknox|TALE

KNKRGVLPKQATNVMRSWLFQHIAHPYPTEEEKKQIATQTNLTLLQVNNWFINARRRILQPM

>Zebrafish|pknox1.2|Pknox|TALE

SKAKRGILPKHATNVMRSWLFQHIGHPYPTEDEKKQIALQTNLTLLQVNNWFINARRRILQPM

>Zebrafish|pknox1.3|Pknox|TALE

KNKRGVLPKQATNVMRSWLFQHIAHPYPTEEEKKQIATQTNLTLLQVNNWFINARRRILQPM

>Human|PKNOX2|Pknox|TALE

SKNKRGVLPKHATNIMRSWLFQHLMHPYPTEDEKRQIAAQTNLTLLQVNNWFINARRRILQPM

>Chicken|PKNOX2|Pknox|TALE

SKNKRGVLPKHATNIMRSWLFQHLMHPYPTEDEKRQIAAQTNLTLLQVNNWFINARRRILQPM

>Zebrafish|pknox2|Pknox|TALE

SKNKRGVLPKHATNIMRSWLFQHLMHPYPTEDEKRQIAAQTNLTLLQVNNWFINARRRILQPM

>Zebrafish|pnx|unassigned|Other

KRIRTAFTLDQLRILERSFQSSHYLSVFERHCIASALGLSETQVKIWFQNRRTKWKKE

>Human|POU1F1|Pou1|POU

KRKRRTTISIAAKDALERHFGEQNKPSSQEIMRMAEELNLEKEVVRVWFCNRRQREKRVK

>Chicken|POU1F1|Pou1|POU

KRKRRTTISISAKEALERHFGEQSKPSSQEIMRMAEGLNLEKEVVRVWFCNRRQREKRVK

>Zebrafish|pou1f1|Pou1|POU

KRKRRTTISLGAKEALERSFVEKSKPSSQEIVRMAEGLHLEKEVVRVWFCNRRQREKRVK

>Zebrafish|Pou2-l|Pou2|POU

KRKKRTSIETNIKLTLEKRFLDNPKPNSEEITLISEQLAMEKEVVRVWFCNRRQKEKRI

>Human|POU2F1|Pou2|POU

RRKKRTSIETNIRVALEKSFLENQKPTSEEITMIADQLNMEKEVIRVWFCNRRQKEKRIN

>Chicken|POU2F1|Pou2|POU

RRKKRTSIETNIRVALEKSFLENQKPTSEEITMIADQLNMEKEVIRVWFCNRRQKEKRIN

>Zebrafish|pou2f1a|Pou2|POU

RRKKRTSIETSIRIALERSFLEQSQKPSSEEITLIADQLSMEKEVVRVWFCNRRQKEKRIN

>Zebrafish|pou2f1b|Pou2|POU

RRKKRTSIETNIRVALEKSFLEQNQKPTSEEITMIADQLNMEKEVIRVWFCNRRQKEKRIN

>Human|POU2F2|Pou2|POU

RRKKRTSIETNVRFALEKSFLANQKPTSEEILLIAEQLHMEKEVIRVWFCNRRQKEKRIN

>Zebrafish|pou2f2|Pou2|POU

RRKKRTSIETNVRVALEHSFLANQKPTSEEILLISEKLNMEKEVIRVWFCNRRQKEKRIN

>Chicken|Pou2f2-l|Pou2|POU

QNQKPTSEEILLIAEQLHMEKEVIRVWFCNRRQKEKR

>Human|POU2F3|Pou2|POU

KRKKRTSIETNIRLTLEKRFQDNPKPSSEEISMIAEQLSMEKEVVRVWFCNRRQKEKRIN

>Chicken|POU2F3|Pou2|POU

KRKKRTSIETNIRSTLEKRFQDNPKPSSEEISLIAEQLSMEKEVVRVWFCNRRQKEKRIS

>Human|POU3F1|Pou3|POU

KRKKRTSIEVGVKGALESHFLKCPKPSAHEITGLADSLQLEKEVVRVWFCNRRQKEKRMT

>Zebrafish|pou3f1|Pou3|POU

KRKKRTSIEVGVKGALENHFLKCPKPSAHEITTLAGTLQLEKEVVRVWFCNRRQKEKRMT

>Human|POU3F2|Pou3|POU

KRKKRTSIEVSVKGALESHFLKCPKPSAQEITSLADSLQLEKEVVRVWFCNRRQKEKRMT

>Zebrafish|pou3f2|Pou3|POU

KRKKRTSIEVSVKGALESHFLKCPKPAASEITSLADSLQLEKEVVRVWFCNRRQKEKRMT

>Human|POU3F3|Pou3|POU

KRKKRTSIEVSVKGALESHFLKCPKPSAQEITNLADSLQLEKEVVRVWFCNRRQKEKRMT

>Zebrafish|pou3f3a|Pou3|POU

KRKKRTSIEVSVKGALESHFLKCPKPSAQEITSLADNLQLEKEVVRVWFCNRRQKEKRMT

>Zebrafish|pou3f3b|Pou3|POU

KRKKRTSIEVSVKGALESHFLKCPKPSAQEITSLADNLQLEKEVVRVWFCNRRQKEKRMT

>Human|POU3F4|Pou3|POU

KRKKRTSIEVSVKGVLETHFLKCPKPAAQEISSLADSLQLEKEVVRVWFCNRRQKEKRMT

>Human|POU4F1|Pou4|POU

KKRKRTSIAAPEKRSLEAYFAVQPRPSSEKIAAIAEKLDLKKNVVRVWFCNQRQKQKRMK

>Zebrafish|pou4f1|Pou4|POU

KKRKRTSIAAPEKRSLEAYFAVQPRPSSEKIAAIAEKLDLKKNVVRVWFCNQRQKQKRLK

>Human|POU4F2|Pou4|POU

KKRKRTSIAAPEKRSLEAYFAIQPRPSSEKIAAIAEKLDLKKNVVRVWFCNQRQKQKRMK

>Zebrafish|pou4f2|Pou4|POU

KKRKRTSIAAPEKRSLEAYFAIQPRPSSEKIAAIAEKLDLKKNVVRVWFCNQRQKQKRMK

>Human|POU4F3|Pou4|POU

RKRKRTSIAAPEKRSLEAYFAIQPRPSSEKIAAIAEKLDLKKNVVRVWFCNQRQKQKRMK

>Zebrafish|pou4f3|Pou4|POU

RKRKRTSIAAPEKRSLEAYFAIQPRPSSEKIAAIAEKLDLKKNVVRVWFCNQRQKQKRMK

>Human|POU5F1|Pou5|POU

RKRKRTSIENRVRGNLENLFLQCPKPTLQQISHIAQQLGLEKDVVRVWFCNRRQKGKRSS

>Zebrafish|pou5f1|Pou3|POU

KRKRRTSLEGTVRSALESYFVKCPKPNTLEITHISDDLGLERDVVRVWFCNRRQKGKRL

>Human|POU5F2|Pou5|POU

GKWRRASRERRIGNSLEKFFQRCPKPTPQQISHIAGCLQLQKDVVRVWFYNRSKMGSRPT

>Human|POU6F1|Pou6|POU

KRKRRTSFTPQAIEALNAYFEKNPLPTGQEITEIAKELNYDREVVRVWFCNRRQTLKNTS

>Zebrafish|pou6f1|Pou6|POU

KRKRRTSFTPQAIEVLNTYFEKNSLPTGQEITEIAKELNYDREVVRVWFCNRRQTLKNTS

>Human|POU6F2|Pou6|POU

KRKRRTSFTPQALEILNAHFEKNTHPSGQEMTEIAEKLNYDREVVRVWFCNKRQALKNTI

>Chicken|POU6F2|Pou6|POU

KRKRRTSFTPQALEILNAHFEKNTHPSGQEMTEIAEKLNYDREVVRVWFCNKRQALKNTI

>Chicken|POUV|Pou3|POU

KRKRRTSIETNVKGTLESFFRKCVKPSPQEISQIAEDLNLDKDVVRVWFCNRRQKGKRL

>Human|PROP1|Prop|PRD

RRRHRTTFSPVQLEQLESAFGRNQYPDIWARESLARDTGLSEARIQVWFQNRRAKQRKQE

>Zebrafish|prop1|Prop|PRD

TTFSNEQLEHLELAFRQNHYPDIYYREELARVTKLNEARIQVWFQNRRAKQRKQD

>Human|PROX1|Prox|PROS

GSAMQEGLSPNHLKKAKLMFFYTRYPSSNMLKTYFSDVKFNRCITSQLIKWFSNFREFYYIQM

>Chicken|PROX1|Prox|PROS

GSAMQEGLSPNHLKKAKLMFFYTRYPSSNMLKTYFSDVKFNRCITSQLIKWFSNFREFYYIQM

>Zebrafish|prox1|Prox|PROS

GSTIQEGLSPNHLKKAKLMFFYTRYPSSNMLKMFFSDVKFNRCITSQLIKWFSNFREFYYIQM

>Zebrafish|prox1b|Prox|PROS

QEGLSPCHLKKAKLMFFYTRYPSSSTLKTYFPDVKFNRCVTSQLIKWFSNFREFFYIQM

>Human|PROX2|Prox|PROS

LVHIQEGLNPGHLKKAKLMFFFTRYPSSNLLKVYFPDVQFNRCITSQMIKWFSNFREFYYIQM

>Chicken|Prox2|Prox|PROS

HIQEGLSPGHLKKAKLMFFFTRYPSSSLLKAYFPDVQFNRCITSQMIKWFSNFREFYYIQM

>Zebrafish|prox2|Prox|PROS

IQEGLTPNHLKKAKLMFFYTRYPSSNLLKNFFPDVKFNRCITSQLIKWFSNFREFYYIQM

>Human|PRRX1|Prrx|PRD

QRRNRTTFNSSQLQALERVFERTHYPDAFVREDLARRVNLTEARVQVWFQNRRAKFRRNE

>Chicken|PRRX1|Prrx|PRD

QRRNRTTFNSSQLQALERVFERTHYPDAFVREDLARRVNLTEARVQVWFQNRRAKFRRNE

>Zebrafish|prrx1a|Prrx|PRD

QRRNRTTFNSSQLQALERVFERTHYPDAFVREDLARRVNLTEARVQVWFQNRRAKFRRNE

>Zebrafish|prrx1b|Prrx|PRD

QRRNRTTFNSSQLQALERVFERTHYPDAFVREDLARRVNLTEARVQVWFQNRRAKFRRNE

>Human|PRRX2|Prrx|PRD

QRRNRTTFNSSQLQALERVFERTHYPDAFVREELARRVNLSEARVQVWFQNRRAKFRRNE

>Chicken|PRRX2|Prrx|PRD

QRRNRTTFNSSQLQALERVFERTHYPDAFVREELARRVNLSEARVQVWFQNRRAKFRRNE

>Human|RAX|Rax|PRD

HRRNRTTFTTYQLHELERAFEKSHYPDVYSREELAGKVNLPEVRVQVWFQNRRAKWRRQE

>Human|RAX2|Rax|PRD

HRRNRTTFTTYQLHQLERAFEASHYPDVYSREELAAKVHLPEVRVQVWFQNRRAKWRRQE

>Human|RHOXF1|Rhox|PRD

PRTRRTKFTLLQVEELESVFRHTQYPDVPTRRELAENLGVTEDKVRVWFKNKRARCRRHQ

>Human|RHOXF2|Rhox|PRD

QQPNVHAFTPLQLQELERIFQREQFPSEFLRRRLARSMNVTELAVQIWFENRRAKWRRHQ

>Human|RHOXF2B|Rhox|PRD

QQPNVHAFTPLQLQELECIFQREQFPSEFLRRRLARSMNVTELAVQIWFENRRAKWRRHQ

>Zebrafish|rx1|Rax|PRD

HRRNRTTFTTYQLHELERAFEKSHYPDVYSREELAMKVNLPEVRVQVWFQNRRAKWRRQE

>Chicken|RX2|Rax|PRD

HRRNRTTFTTYQLHELERAFEKSHYPDVYSREELAMKVNLPEVRVQVWFQNRRAKWRRQE

>Zebrafish|rx2|Rax|PRD

HRRNRTTFTTYQLHELERAFEKSHYPDVYSREELAMKVNLPEVRVQVWFQNRRAKWRRQE

>Zebrafish|rx3|Rax|PRD

HRRNRTTFTTFQLHELERAFEKSHYPDVYSREELALKVNLPEVRVQVWFQNRRAKWRRQE

>Human|SATB1|Satb|CUT

KTRPRTKISVEALGILQSFIQDVGLYPDEEAIQTLSAQLDLPKYTIIKFFQNQRYYLKHHG

>Chicken|SATB1|Satb|CUT

KPRPRTKISVEALGILQSFIQDVGLYPDEEAIQTLSAQLDLPKYTIIKFFQNQRYYLKHHG

>Human|SATB2|Satb|CUT

KPRSRTKISLEALGILQSFIHDVGLYPDQEAIHTLSAQLDLPKHTIIKFFQNQRYHVKHHG

>Chicken|SATB2|Satb|CUT

KPRSRTKISLEALGILQSFIHDVGLYPDQEAIHTLSAQLDLPKHTIIKFFQNQRYHVKHHG

>Zebrafish|satb2|Satb|CUT

KPRSRTKISLEALGILQSFIQDVGLYPDQEAIHTLSAQLDLPKHTIVKFFQNQRYHVKHHG

>Zebrafish|sb:cb1008|unassigned|Other

KRPRVVLSVQEKQTLFSAYELEPYPSQNTIDRLAAQLGLQTSTVSNWFYNYRSRIRRDGF

>Human|SEBOX|Sebox|PRD

HRRKRTTFSKGQLLELERAFAAWPYPNISTHEHLAWVTCLPEAKVQVWFQKRWAKIIKNR

>Chicken|SEBOX|Sebox|PRD

KRRRTTFSRGQLSELERAFAAVPYPDIATRERLAELTQLPEAKIQVWFQNRRARRIR

>Human|SHOX|Shox|PRD

QRRSRTNFTLEQLNELERLFDETHYPDAFMREELSQRLGLSEARVQVWFQNRRAKCRKQE

>Zebrafish|shox|Shox|PRD

QRRSRTNFTLEQLNELERLFDETHYPDAFMREELSQRLGLSEARVQVWFQNRRAKCRKQE

>Human|SHOX2|Shox|PRD

QRRSRTNFTLEQLNELERLFDETHYPDAFMREELSQRLGLSEARVQVWFQNRRAKCRKQE

>Chicken|SHOX2|Shox|PRD

QRRSRTNFTLEQLNELERLFDETHYPDAFMREELSQRLGLSEARVQVWFQNRKAKCRKQE

>Zebrafish|shox2|Shox|PRD

QRRSRTNFTLEQLNELERLFDETHYPDAFMREELSQRLGLSEARVQVWFQNRRAKCRKQE

>Zebrafish|si:ch211-159p3.1|unassigned|Other

ERLLPLLKAYSQNPEPTEEQLAQVAKTVKLPLAAVTKWYEKMRSKRILLQ

>Zebrafish|si:ch211-251d10.5|Cux|CUT

KKPRVVLAPEEKEALKRAYQQKPYPSPKTIEELASQLNLKTSTVINWFHNYRYQ

>Zebrafish|si:ch211-260g14.3|Lmx|LIM

PKRPRTILTTQQRRAFKASFEVSSKPCRKVRETLAAETGLSVRVVQVWFQNQRAKMKKL

>Zebrafish|si:ch73-386h18.1|HD1|Zfhx|ZF

SKRPRTRITDDQLRVLRQYFDINNSPNEDQIHEMANKSGLPHKVIKHWFRNTLFKERQ

>Zebrafish|si:ch73-386h18.1|HD2|Zfhx|ZF

KRSSRTRFNDYQLRVLQDFFDANAYPKDDEFEQLSNLLNLSTRVIVVWFQNARQKARKNY

>Zebrafish|si:ch73-386h18.1|HD3|Zfhx|ZF

DKRMRTTITPEQLEVLYQKYLLDSNPTRKMLDHISNEVGLKKRVVQVWFQNTRARERKGQ

>Zebrafish|si:ch73-386h18.1|HD4|Zfhx|ZF

KRYRTQMSNLQVKVLKACFSDYKTPTMLECEALGNDIGLAKRVVQVWFQNARAKEKKAK

>Zebrafish|si:dkey-3j24.1|Pou2|POU

RRKKRTSIETNVRIALERNFISNQKPTSEEILLMAEQLNMEKEVIRVWFCNRRQKEKRIN

>Human|SIX1|Six1/2|SINE

GEETSYCFKEKSRGVLREWYAHNPYPSPREKRELAEATGLTTTQVSNWFKNRRQRDRAAE

>Chicken|SIX1|Six1/2|SINE

GEETSYCFKEKSRGVLREWYAHNPYPSPREKRELAEATGLTTTQVSNWFKNRRQRDRAAE

>Zebrafish|six1a|Six1/2|SINE

GEETSYCFKEKSRSVLREWYTHNPYPSPREKRELAEATGLTTTQVSNWFKNRRQRDRAAE

>Zebrafish|six1b|Six1/2|SINE

GEETSYCFKEKSRGVLREWYTHNPYPSPREKRELAEATGLTTTQVSNWFKNRRQRDRAAE

>Human|SIX2|Six1/2|SINE

GEETSYCFKEKSRSVLREWYAHNPYPSPREKRELTEATGLTTTQVSNWFKNRRQRDRAAE

>Zebrafish|six2a|Six1/2|SINE

GEETSYCFKEKSRSVLREWYTHNPYPSPREKRELAEATGLTTTQVSNWFKN

>Zebrafish|six2b|Six1/2|SINE

GEETSYCFKEKSRCVLKEWYTHNPYPSPREKRELAEATGLTTTQVSNWFKNRRQRDRAAE

>Human|SIX3|Six3/6|SINE

GEQKTHCFKERTRSLLREWYLQDPYPNPSKKRELAQATGLTPTQVGNWFKNRRQRDRAAA

>Zebrafish|six3a|Six3/6|SINE

GEQKTHCFKERTRSLLREWYLQDPYPNPSKKRELAQATGLTPTQVGNWFKNRRQRDRAAA

>Zebrafish|six3b|Six3/6|SINE

GEQKTHCFKERTRGLLREWYLQDPYPNPSKKRELAQATGLTPTQVGNWFKNRRQRDRAAA

>Human|SIX4|Six4/5|SINE

GEETVYCFKEKSRNALKELYKQNRYPSPAEKRHLAKITGLSLTQVSNWFKNRRQRDRNPS

>Zebrafish|six4.3|Six4/5|SINE

GEETVYCFKEKSRNALKECYKINRYPTPAEKKNLAKVTGLSLTQVSNWFKNRRQRDRTPS

>Zebrafish|six4a|Six4/5|SINE

GEETVYCFKERSRNALKDLYKQNRYPSPAEKRNLAKITGLSLTQVSNWFKNRRQRDRNPS

>Zebrafish|six4b|Six4/5|SINE

GEETVYCFKERSRNALKDMYKRNRYPSPAEKRNLAKMTGLSLTQVSNWFKNRRQRDRNPS

>Human|SIX5|Six4/5|SINE

GEETVYCFKERSRAALKACYRGNRYPTPDEKRRLATLTGLSLTQVSNWFKNRRQRDRTGA

>Human|SIX6|Six3/6|SINE

GEQKTHCFKERTRNLLREWYLQDPYPNPSKKRELAQATGLTPTQVGNWFKNRRQRDRAAA

>Zebrafish|six6a|Six3/6|SINE

EQKTHCFKERTRHLLREWYLQDPYPNPSKKRELAQATGLTPTQVGNWFKNRRQRDRAAA

>Zebrafish|six6b|Six3/6|SINE

EQKTHCFKERTRHLLREWYLQDPYPNPSKKRELAQATGLTPTQVGNWFKNRRQRDRAAA

>Zebrafish|six7|Six3/6|SINE

GEQKTHCFKERTRSLLREWYLQDPYPNPSRKRHLAQATGLTPTQVGNWFKNRRQRDRAAS

>Zebrafish|six9|Six1/2|SINE

GEETSYCFKEKSRSVLREWYCRKPYPSPREKRDLAAATGLTATQVSNWFKNRRQRDRAA

>Chicken|SOHO-1|Nk5/Hmx|ANTP

KKKTRTIFSKSQVFQLESTFDVKRYLSSAERAGLAAALHLTETQVKIWFQNRRNKLKRQL

>Human|TGIF1|Tgif|TALE

KRRRRGNLPKESVQILRDWLYEHRYNAYPSEQEKALLSQQTHLSTLQVCNWFINARRRLLPDM

>Chicken|TGIF1|Tgif|TALE

NLPKESVQILRDWLYEHRYNAYPSEQEKVLLSRQTHLSTLQVCNWFINARRRLLPDM

>Zebrafish|tgif1|Tgif|TALE

KRKRRGNLPKESVQILRDWLYQHRYNAYPSEQEKALLSKQTHLSTLQVCNWFINARRRLLPEM

>Human|TGIF2|Tgif|TALE

KRKRRGNLPKESVKILRDWLYLHRYNAYPSEQEKLSLSGQTNLSVLQICNWFINARRRLLPDM

>Zebrafish|Tgif2|Tgif|TALE

KRRRRGNLPKESVQVLRDWLYEHRFNAYPSEQEKLSLSGQTHLSVSQICNWFINARRRLLPDL

>Human|TGIF2LX|Tgif|TALE

KKKRKGNLPAESVKILRDWMYKHRFKAYPSEEEKQMLSEKTNLSLLQISNWFINARRRILPDM

>Human|TGIF2LY|Tgif|TALE

KKKRKGNLPAESVKILRDWMYKHRFKAYPSEEEKQMLSEKTNLSLLRISNWFINARRRILPDM

>Human|TLX1|Tlx|ANTP

KKKPRTSFTRLQICELEKRFHRQKYLASAERAALAKALKMTDAQVKTWFQNRRTKWRRQT

>Chicken|TLX1|Tlx|ANTP

KKKPRTSFTRLQICELEKRFHRQKYLASAERAALAKALKMTDAQVKTWFQNRRTKWRRQT

>Zebrafish|tlx1|Tlx|ANTP

KKKPRTSFTRLQICELEKRFHRQKYLASAERAALAKALKMTDAQVKTWFQNRRTKWRRQT

>Human|TLX2|Tlx|ANTP

RKKPRTSFSRSQVLELERRFLRQKYLASAERAALAKALRMTDAQVKTWFQNRRTKWRRQT

>Zebrafish|tlx2|Tlx|ANTP

RKKPRTSFSRVQICELEKRFHRQKYLASAERATLAKALKMTDAQVKTWFQNRRTKWRRQT

>Human|TLX3|Tlx|ANTP

RKKPRTSFSRVQICELEKRFHRQKYLASAERAALAKSLKMTDAQVKTWFQNRRTKWRRQT

>Chicken|TLX3|Tlx|ANTP

RKKPRTSFSRVQICELEKRFHRQKYLASAERAALAKSLKMTDAQVKTWFQNRRTKWRRQT

>Zebrafish|tlx3b|Tlx|ANTP

RKKPRTSFSRVQICELEKRFHRQKYLASAERAALAKTLKMTDAQVKTWFQNRRTKWRRQT

>Human|TPRX1|Tprx|PRD

QRQERTVYTESQQKVLEFYFQKDQYPNYDQRLNLAEMLSLREQQLQVWFKNRRAKLARER

>Human|TPRXL|Tprx|PRD

QRQDRTIYNWKQQEVLENHFKEEQYPDYDTRQELAEMLNLREYQVQVWFKNRRAKRSRER

>Zebrafish|trh1|unassigned|Other

VRDRVRLHITPVPKLEAFYLQKKKTEPSQEDLQALVKLCGLSERQIQSWLRRRRIQDRPA

>Human|TSHZ1|Tshz|ZF

RKGRQSNWNPQHLLILQAQFASSLRETTEGKYIMSDLGPQERVHISKFTGLSMTTISHWLANVKYQLRRTG

>Chicken|TSHZ1|Tshz|ZF

RKGRQSNWNPQHLLILQAQFASSLRETSEGKYIMSDLGPQERVHISKFTGLSMTTISHWLANVKYQLRRTG

>Zebrafish|tshz1|Tshz|ZF

RKGRQSNWNPQHLLILQAQFASSLRETPDGKYIITDLGPQERVHICKFTGLSMTTISHWLANVKYQLRRTG

>Human|TSHZ2|Tshz|ZF

RKGRQSNWNPQHLLILQAQFASSLFQTSEGKYLLSDLGPQERMQISKFTGLSMTTISHWLANVKYQLRKTG

>Chicken|TSHZ2|Tshz|ZF

RKGRQSNWNPQHLLILQAQFASSLFQTSEGKYLLSDLGPQERMQISKFTGLSMTTISHWLANVKYQLRKTG

>Zebrafish|tshz2|Tshz|ZF

RKGRQSNWNPQHLLILQAQFASSLFQTSEGKYLLSDLGPQERMHISKFTGLSMTTISHWLANVKYQLRKTG

>Human|TSHZ3|Tshz|ZF

RKGRQSNWNPQHLLILQAQFAASLRQTSEGKYIMSDLSPQERMHISRFTGLSMTTISHWLANVKYQLRRTG

>Chicken|TSHZ3|Tshz|ZF

RKGRQSNWNPQHLLILQAQFAASLRQTSEGKYIMSDLSPQERMHISRFTGLSMTTISHWLANVKYQLRRTG

>Zebrafish|tshz3b|Tshz|ZF

RKGRQSNWNPQHLLILQAQFASSLRQTGDGKYIMSDLSPQERMHISRFTGLSMTTISHWLANVKYQLRRTG

>Human|UNCX|Uncx|PRD

RRRTRTNFTGWQLEELEKAFNESHYPDVFMREALALRLDLVESRVQVWFQNRRAKWRKKE

>Chicken|UNCX|Uncx|PRD

RRRTRTNFTGWQLEELEKAFNESHYPDVFMREALALRLDLVESRVQV

>Zebrafish|uncx|Uncx|PRD

RRRTRTNFTGWQLEELEKAFNESHYPDVFMREALALRLDLIESRVQVWFQNRRAKWRKKE

>Zebrafish|uncx4.1|Uncx|PRD

RRRTRTNFTGWQLEELEKASNESHYPDVFMREALALRLDLVESRVQVWFQNRRAKWRKKE

>Zebrafish|Uncxl-2|Uncx|PRD

QRRARANYSSWQLEELEKTFQSTHYPDIFMREALALRLDLIEARVQVWFQNRRAKMRRQL

>Human|VAX1|Vax|ANTP

PKRTRTSFTAEQLYRLEMEFQRCQYVVGRERTELARQLNLSETQVKVWFQNRRTKQKKDQ

>Chicken|VAX1|Vax|ANTP

PKRTRTSFTAEQLYRLEMEFQRCQYVVGRERTELARQLNLSETQVKVWFQNRRTKQKKDQ

>Zebrafish|vax1|Vax|ANTP

PKRTRTSFTAEQLYRLEMEFQRCQYVVGRERTELARQLNLSETQVKVWFQNRRTKQKKDQ

>Human|VAX2|Vax|ANTP

PKRTRTSFTAEQLYRLEMEFQRCQYVVGRERTELARQLNLSETQVKVWFQNRRTKQKKDQ

>Zebrafish|vax2|Vax|ANTP

PKRTRTSFTAEQLYRLELEFQRCQYVVGRERTELARQLNLSETQVKVWFQNRRTKQKKDQ

>Zebrafish|ved|unassigned|Other

RRPRTAFSSEQISSLERVFKRNAYLGAQDKAELCRTLKLTDKQIRNWFQNRRMKLKR

>Human|VENTX|Ventx|ANTP

APRVRTAFTMEQVRTLEGVFQHHQYLSPLERKRLAREMQLSEVQIKTWFQNRRMKHKRQM

>Chicken|VENTX|Ventx|ANTP

RLRTAFSAEQISTLESSFQRHRYLGAAERRKLAGRMRLSEVQIKTWFQNRRMKLKRQL

>Zebrafish|vox|unassigned|Other

RIRTKFTPEQIDKLEKIFNKHKYLDAGERVKTALKLGLSETQIRTWFQNRRMKLKREV

>Human|VSX1|Vsx|PRD

KRRHRTVFTAHQLEELEKAFSEAHYPDVYAREMLAVKTELPEDRIQVWFQNRRAKWRKRE

>Chicken|VSX1|Vsx|PRD

KRRHRTVFTAHQLEELEKAFNEAHYPDVYAREMLAVKTELPEDRIQVWFQNRRAKWRKRE

>Zebrafish|vsx1|Vsx|PRD

KRRHRTVFTSHQLEELEKAFNEAHYPDVYAREMLAMKTELPEDRIQVWFQNRRAKWRKRE

>Human|VSX2|Vsx|PRD

KRRHRTIFTSYQLEELEKAFNEAHYPDVYAREMLAMKTELPEDRIQVWFQNRRAKWRKRE

>Chicken|VSX2|Vsx|PRD

KRRHRTIFTSYQLEELEKAFNEAHYPDVYAREMLAMKTELPEDRIQVWFQNRRAKWRKRE

>Zebrafish|vsx2|Vsx|PRD

KRRHRTIFTSYQLEELEKAFNEAHYPDVYAREMLAMKTELPEDRIQVWFQNRRAKWRKRE

>Zebrafish|wu:fa03a11|HD1|Zfhx|ZF

TSSPQREDCEALGREVGLSGEEVRKWF

>Zebrafish|wu:fa03a11|HD2|Zfhx|ZF

RRSRTIIHADQLDILYGCYFKDPNPGKHEFEQISEWVNLPKKVVQIWFQNMRARERKG

>Zebrafish|wu:fa03a11|HD3|Zfhx|ZF

RRPRTHLTSLQLSILQSCYETCAHPNALECEAVGTELGLPLKVVQIWFQNTRAKEKR

>Zebrafish|wu:fi69e09|Nobox|PRD

KKKTRTFYSTDQLEELERVFQDDHYPDGDKRKEIAAAIGVTPQRIMVWFQNRRAKWRK

>Human|ZEB1|Zeb|ZF

GNLSPSQPPLKNLLSLLKAYYALNAQPSAEELSKIADSVNLPLDVVKKWFEKMQAGQISVQ

>Chicken|ZEB1|Zeb|ZF

NLSPGQPPLKNLLSLLKAYYALNAQPSAEELSKIADSVNLPLDVVKKWFEKMQAGQISVQ

>Zebrafish|zeb1|Zeb|ZF

LCPGQPPLKNLLSLLKAYFALNNEPTKEELAKISESVSLPAEVVKKWFEKMQLGQISM

>Human|ZEB2|Zeb|ZF

GMTSPINPYKDHMSVLKAYYAMNMEPNSDELLKISIAVGLPQEFVKEWFEQRKVYQYSNS

>Chicken|ZEB2|Zeb|ZF

GMTSPINPYKDHMSVLKAYYAMNMEPNSDELLKISIAVGLPQEFVKEWFEQRKVYQY

>Zebrafish|zeb2b|Zeb|ZF

LNGPISPYKDHMSVLKAYFAMNMEPNSEELLKISIAVGLPQEFVKEWFEQRKVFQYTTS

>Human|ZFHX2|HD1|Zfhx|ZF

RRFSRTKFTEFQTQALQSFFETSAYPKDGEVERLASLLGLASRVVVVWFQNARQKARKNA

>Human|ZFHX2|HD2|Zfhx|ZF

DKRLRTTILPEQLEILYRWYMQDSNPTRKMLDCISEEVGLKKRVVQVWFQNTRARERKGQ

>Human|ZFHX2|HD3|Zfhx|ZF

QRRYRTQMSSLQLKIMKACYEAYRTPTMQECEVLGEEIGLPKRVIQVWFQNARAKEKKAK

>Human|ZFHX3|HD1|Zfhx|ZF

NKRPRTRITDDQLRVLRQYFDINNSPSEEQIKEMADKSGLPQKVIKHWFRNTLFKERQRN

>Human|ZFHX3|HD2|Zfhx|ZF

KRSSRTRFTDYQLRVLQDFFDANAYPKDDEFEQLSNLLNLPTRVIVVWFQNARQKARKNY

>Human|ZFHX3|HD3|Zfhx|ZF

DKRLRTTITPEQLEILYQKYLLDSNPTRKMLDHIAHEVGLKKRVVQVWFQNTRARERKGQ

>Human|ZFHX3|HD4|Zfhx|ZF

QKRFRTQMTNLQLKVLKSCFNDYRTPTMLECEVLGNDIGLPKRVVQVWFQNARAKEKKSK

>Chicken|ZFHX3|HD1|Zfhx|ZF

NKRPRTRITDDQLRVLRQYFDINNSPSEEQIKEMADKSGLPQKVIKHWFRNTLFKERQRN

>Chicken|ZFHX3|HD2|Zfhx|ZF

KRSSRTRFTDYQLRVLQDFFDANAYPKDDEFEQLSNLLNLPTRVIVVWFQNARQKARKNY

>Chicken|ZFHX3|HD3|Zfhx|ZF

DKRLRTTITPEQLEILYQKYLLDSNPTRKMLDHIAHEVGLKKRVVQVWFQNTRARERKGQ

>Chicken|ZFHX3|HD4|Zfhx|ZF

KRFRTQMTNLQLKVLKSCFNDYRTPTMLECEVLGNDIGLPKRVVQVWFQNARAKEKKSK

>Zebrafish|Zfhx3-l|HD1|Zfhx|ZF

RTRITDDQLRVLRQYFDINNSPNEEQIKEMADKSGLPQKVIKHWFRNTLFKERQ

>Zebrafish|Zfhx3-l|HD2|Zfhx|ZF

KRSSRTRFTDYQLRVLQDFFDANAYPKDDEFEQLSNLLNLPTRVIVVWFQNARQKARKNY

>Zebrafish|Zfhx3-l|HD3|Zfhx|ZF

RTTITPEQLEILYQKYLLDSNPTRKMLDHIAHEVGLKKRVVQVWFQNTRARERK

>Zebrafish|Zfhx3-l|HD4|Zfhx|ZF

RTQMTNLQLKVLKSCFSDYRTPTMLECEVLGNDIGLPKRVVQVWFQNARAKEKK

>Human|ZFHX4|HD1|Zfhx|ZF

FKRPRTRITDDQLKILRAYFDINNSPSEEQIQEMAEKSGLSQKVIKHWFRNTLFKERQRN

>Human|ZFHX4|HD2|Zfhx|ZF

KRSSRTRFTDYQLRVLQDFFDTNAYPKDDEIEQLSTVLNLPTRVIVVWFQNARQKARKSY

>Human|ZFHX4|HD3|Zfhx|ZF

DKRLRTTITPEQLEILYEKYLLDSNPTRKMLDHIAREVGLKKRVVQVWFQNTRARERKGQ

>Human|ZFHX4|HD4|Zfhx|ZF

HKRFRTQMSNLQLKVLKACFSDYRTPTMQECEMLGNEIGLPKRVVQVWFQNARAKEKKFK

>Chicken|ZFHX4|HD1|Zfhx|ZF

FKRPRTRITDDQLKILRAYFDINNSPSEEQIQEMAEKSGLSQKVIKHWFRNTLFKERQRN

>Chicken|ZFHX4|HD2|Zfhx|ZF

SSRTRFTDYQLRVLQDFFDTNAYPKDDEIEQLSTVLNLPTRVIVVWFQNARQKAR

>Chicken|ZFHX4|HD3|Zfhx|ZF

DKRLRTTITPEQLEILYEKYLLDSNPTRKMLDHIAREVGLKKRVVQVWFQNTRARERKGQ

>Chicken|ZFHX4|HD4|Zfhx|ZF

HKRFRTQMSNLQLKVLKACFSDYRTPTMQECEMLGNEIGLPKRVVQVWFQNARAKEKKFK

>Zebrafish|zgc:158291|Pou4|POU

KKRKRTSIAAPEKRSLEAYFAVQPRPSSEKIAAIAEKLDLKKNVVRVWFCNQRQKQKRMK

>Zebrafish|zgc:171494|unassigned|Other

VKDKIRKRASHNPLLETYYRTHSKHPTQADVKGVCKKLGWSERQVERWFRQRRNQDRPGV

>Zebrafish|zgc:193933|Ventx|ANTP

RKTRAAFSEEQMNALVNRFNVQRYLTPAEMKTLAGATGLTYKQVKTWFQNRRMKLKRHQ

>Zebrafish|zgc:91944|Hmbox|HNF

RRGSRFTWRKECQSIMESFFIENQYPDEAKREEIANACNAVIQKPGCKLSEFERVTALKVYNWFANRRKEMKRRA

>Zebrafish|Zhl-l|Zhx/Homez|ZF

SSIPTYDPAMDLSSFLKTSFGKFPYPTKAELCYLTVVSGFPEEQIKLWFTAQRLKQGISW

>Zebrafish|Zhx-l|HD1|Zhx/Homez|ZF

LVEAFNSFPYPTSQEASALARKCALPPDQVKAWF

>Zebrafish|Zhx-l|HD2|Zhx/Homez|ZF

KKSKAQLMALRRSFVNKSWPSDGEVQRLQKMTGLSRREIRKWFADSRYQLRKNGR

>Zebrafish|Zhx-l|HD3|Zhx/Homez|ZF

KKTREQLEMLRQSFLCCQWPTSDDYEILQKKTGLTRTEIIQWYGDTRYHVKHNQ

>Human|ZHX1|HD1|Zhx/Homez|ZF

NSIPTYNAALDNNPLLLNTYNKFPYPTMSEITVLSAQAKYTEEQIKIWFSAQRLKHGVSW

>Human|ZHX1|HD2|Zhx/Homez|ZF

SFGIRAKKTKEQLAELKVSYLKNQFPHDSEIIRLMKITGLTKGEIKKWFSDTRYNQRNSK

>Human|ZHX1|HD3|Zhx/Homez|ZF

TPQKFKEKTAEQLRVLQASFLNSSVLTDEELNRLRAQTKLTRREIDAWFTEKKKSKALKE

>Human|ZHX1|HD4|Zhx/Homez|ZF

STGKICKKTPEQLHMLKSAFVRTQWPSPEEYDKLAKESGLARTDIVSWFGDTRYAWKNGN

>Human|ZHX1|HD5|Zhx/Homez|ZF

DRGPSLIKFKTGTAILKDYYLKHKFLNEQDLDELVNKSHMGYEQVREWFAERQRRSELGI

>Chicken|ZHX1|HD1|Zhx/Homez|ZF

NSIPTYNTALDNNPLLLNTYNKFPYPTMSEITVLSTQAKYTEEQIKIWFSAQRLKHGVSW

>Chicken|ZHX1|HD2|Zhx/Homez|ZF

TFGMRAKKTKEQLAELKVSYLKNQFPQDSEISRLMKITGLTKGEIKKWFSDTRYNQRNSK

>Chicken|ZHX1|HD3|Zhx/Homez|ZF

TPQKFKEKTAEQLQVLQASFLNNPILTDEEMNRLRAQTKLTRREIDAWFTERRKSNVLKE

>Chicken|ZHX1|HD4|Zhx/Homez|ZF

PEQLHMLKSSFVRTQWPSPQEYNKLAEETGLPRSEIVSWFGDTRYAWKNG

>Chicken|ZHX1|HD5|Zhx/Homez|ZF

DRGVSVIKFKTGTAILKDYYMKHKFLNEQDLDELVAKSHMGYEQVREWFAERQRR

>Human|ZHX2|HD1|Zhx/Homez|ZF

LNTTKYNSALDTNATMINSFNKFPYPTQAELSWLTAASKHPEEHIRIWFATQRLKHGISW

>Human|ZHX2|HD2|Zhx/Homez|ZF

TPASDRKKTKEQIAHLKASFLQSQFPDDAEVYRLIEVTGLARSEIKKWFSDHRYRCQRGI

>Human|ZHX2|HD3|Zhx/Homez|ZF

APQKFKEKTQGQVKILEDSFLKSSFPTQAELDRLRVETKLSRREIDSWFSERRKLRDSME

>Human|ZHX2|HD4|Zhx/Homez|ZF

SPSPAIAKSQEQVHLLRSTFARTQWPTPQEYDQLAAKTGLVRTEIVRWFKENRCLLKTGT

>Chicken|ZHX2|HD1|Zhx/Homez|ZF

LNSTKYNSALDTNATMINSFNKFPYPTQAELSWLTAASKHPEEQIRIWFATQRLKHGISW

>Chicken|ZHX2|HD2|Zhx/Homez|ZF

TPASERKKTKEQIAELRASFIASQFPDDTEVYRLIEATGLSRSEIKKWFSDHRYRSQRGI

>Chicken|ZHX2|HD3|Zhx/Homez|ZF

APQRFKEKSQEQLRALEESFLRCSFPTQGELDRLRVETKLSRREIDSWFSERRKIRDSME

>Chicken|ZHX2|HD4|Zhx/Homez|ZF

SATLKKTQEQIHLLKSTFARTQWPTPQEYDQLASQTGLTRTEIVRWFKENRSSLRSG

>Zebrafish|zhx2|HD1|Zhx/Homez|ZF

LNTSKYNPSLDDNLTLITSFNKFPYPTQAELSWLTAASKHPEEQIKVWFTTQRLKQGISW

>Zebrafish|zhx2|HD2|Zhx/Homez|ZF

EKTPDQIRELTASYAQCQFPDDEEVYRLIETTGLSWGEIKKWFSDQR

>Zebrafish|zhx2|HD3|Zhx/Homez|ZF

SSEQMKKLEESFQRTSFPTQAEMEHLVADTRLSKNEIDCWFTERRA

>Zebrafish|zhx2|HD4|Zhx/Homez|ZF

LCLLKDMFAQTQWPSPEEYNQLEVQTGLARTEIVRWFKDNRSALKN

>Zebrafish|zhx2|HD5|Zhx/Homez|ZF

LCLLKDMFAQTQWPSPEEYNQLEVQTGLARTEIVRWFKDNRSALKN

>Human|ZHX3|HD1|Zhx/Homez|ZF

SSIPTYNAAMDSNSFLKNSFHKFPYPTKAELCYLTVVTKYPEEQLKIWFTAQRLKQGISW

>Human|ZHX3|HD2|Zhx/Homez|ZF

ASIYKNKKSHEQLSALKGSFCRNQFPGQSEVEHLTKVTGLSTREVRKWFSDRRYHCRNLK

>Human|ZHX3|HD3|Zhx/Homez|ZF

TPTKYKERAPEQLRALESSFAQNPLPLDEELDRLRSETKMTRREIDSWFSERRKKVNAEE

>Human|ZHX3|HD4|Zhx/Homez|ZF

PGKVSCKKTAQQRHLLRQLFVQTQWPSNQDYDSIMAQTGLPRPEVVRWFGDSRYALKNGQ

>Human|ZHX3|HD5|Zhx/Homez|ZF

FPPGLLVIAPGNRELLQDYYMTHKMLYEEDLQNLCDKTQMSSQQVKQWFAEKMGEETRAV

>Chicken|ZHX3|HD1|Zhx/Homez|ZF

LKNSFHKFPYPTKAELCYLTVVTKYPEEQLKIWFTAQRLKQGI

>Chicken|ZHX3|HD2|Zhx/Homez|ZF

SSIPTYNAAMDSNSFLKNSFHKFPYPTKAELCYLTVVTKYPEEQLKIWFTAQRLKQGISW

>Chicken|ZHX3|HD3|Zhx/Homez|ZF

NKKSHEQLSALKGSFCRNQFPGQAEVERLTKITGLSTKEIRKWFSDRRYHYRNVR

>Chicken|ZHX3|HD4|Zhx/Homez|ZF

TKYKERAPEQLKALENSFAQNPQPPEEEVNRLRGETKMTRREIDSWFT

>Chicken|ZHX3|HD5|Zhx/Homez|ZF

KLNFKKTAQQRHLLKQMFVQTQRPTNQEYDAIVSQTGLPRAEVIRWFGDSRYGYKNGQ

>Zebrafish|zhx3|HD1|Zhx/Homez|ZF

LKTSFSKFPYPTKAELCYLTVVTKYPEEQIKIWFTAQRLKQGI

>Zebrafish|zhx3|HD2|Zhx/Homez|ZF

NKTKKSQEQLSALKESFLISQFPNQEEVDRLINLTGLSVREVRKWFSDRRYHF

>Zebrafish|zhx3|HD3|Zhx/Homez|ZF

PHQVRALEASFTQEPDPTSEEVDRLRAETKMTRREIHGWFAERRRRV

>Zebrafish|zhx3|HD4|Zhx/Homez|ZF

QYRGKKTPEQLHLLKQVFARTHWPSSPQYNELITKTGLPRPEVVRWFGDCRYVLKN

>Zebrafish|zgc:193933|Vent|ANTP

RRVRTKFTCDQISGLEKSFSKHRYLGATQRRKIAEKLHLSETQVKTWFQNRRMKLKREVQ

>chicken|nanog_a|antp

KAKSRTAFSQEQLQTLHQRFQSQKYLSPHQIRELAAALGLTYKQVKTWFQNQRMKFKRCQ

>chicken|nanog_b|antp

KAKSCTAFSQEQQQILHQLLQSQKYLSPHQIQELAAAQGLTYQQVKTWFQNQRMKMKRCQ

>Zebrafish|Nanog|antp

PRKTRAAFSEEQMNALVNRFNVQRYLTPAEMKTLAGATGLTYKQVKTWFQNRRMKLKRHQ

**Section 2**

>gi_731274091_ref_XP_010606146.1__PREDICTED_homeobox_protein_NANOG_Fukomys_damarensis

MRMGLTEAPPGIPHELRVLQPPTEAPRADAAASPLPSSVGLLLQEPPDSSTSPHITPPSCLEEASGKKEEGKVPGKKQKVRTVFSQTQLCVLNDRFQRQKYLSLQQMQELSSLLNLSYKQVKTWFQNQRMKCKRWQKSNWPQNSTSTPQKGPSPAEYPSVCSNYAQGYLVNTSGNPVWGNQTWNNAGWGSPSWNPPQAWCPQAWNSPIPGCGEEALQPCLPFPQNFPASDLEAVLEAVGDNYKYLSAPQSLDLLLNYPPNPQPEDL

>gi_884915820_ref_XP_013008419.1__PREDICTED_homeobox_protein_NANOG_Cavia_porcellus (Used in Figure 1C, mammal NANOG group)

MSVAPDCPPSLSCSEAAGSGDPSPTPEVPRPAETCGALQPPEAEQPLAESAASPITSSGNLLTQETPDSSIPCATLPSSLEALGRKEEGKVAGRKLKPYTVLSKMQLCVLRDRFQRQQYLNLQHIQELSSLLNLSCKQIKTWFQNQRMKCKKWQKSTGLQNGSGAPEKVPAPAEYPGVCSNYAQSCLVNTSGNLPMWGNQTWNNSAWSSQTWGSQSWGNHSWNTETWNTQSWCPQAWNSPFPSFGEESLQPCLPFPQNFPTSNLEAMLEAVGDSQEYCTTPRPWIFS

>gi_533129096_ref_XP_005379670.1__PREDICTED_homeobox_protein_NANOG_Chinchilla_lanigera

MNMENNSQSASPLPSSGDLLTQATPDSSTSPCVTSPCVALPGSLESSGRKEEAAKVPAKKQKVRTVFSQTQLCVLNDRFQRQKYLSLQQMQELSSLLNLSYKQVKTWFQNQRMKCKRWQKSNWPKSSAGASQTGPASAEYPGSNYTQGCMVNTSGNFPVWGNQTWNSPTWSSQSWSNHSWNTQTWNTQTWNAQTWNTQTWYPQGWNSFPAGGEESLQPCLPLPQNFSASDLEATLEAAGDGHKYFNTPQALDLFLNYAMNLQPEDL

>gi_507711545_ref_XP_004647188.1__PREDICTED_homeobox_protein_NANOG_Octodon_degus

MSVDPDCPPTLSCSEAAESGDPSPKPEVPGPAETYDSPQMTEADVSLCEAASPFPSSGDLHVQETPDSSTSPCVPLPDSLEALPKKGEVKVRMKKQKVRTVFTQTQLCVLKDRFQRQKYLSLQQMQELSSFLNLNYKQVKTWFQNQRMKYKRWQKSNWQNSADAPQKGPAPVEYPGTCSNYAQGNLVNTSGNLPMWSNHTWNNSAWNTQSWANHSWNTQTWNSQTCNMQTWNAQTWNAQTWYPQGWNSPYPSCGEEPLQPCLPFPQSLPANDLEASLEVAGDSQKYLNSPQALDLFLNYAMSLQPEDL

>gi_507570058_ref_XP_004668641.1__PREDICTED_homeobox_protein_NANOG_Jaculus_jaculus

MSVDPACSHSSCSEEPSDPEDMSPMPVIYGSEENYFSLQISSTEMHHTETASLPSSVDLPVQDSPDSSTSPKLELPGSSSEEGMGKRGESKIQVKKQKVRTVFSQAQLCVLNQRFQKQKYLSLQQMQELSSSLDLSYKQVKTWFQNQRMKYKRWQKNNWSKTSHSVTQKGSTCIEYPGLHSSCLPGFLVNPSGNLPVWSNQTWNNPAWSGQNWSSHSWSTPAWHSPVWSSQPWGAPFHNSGEDSLQPYVQFQQHFSGNDLEASLETVGENSKYLNTPQALDVLNYSVNMPPDGM

>gi_674105035_ref_XP_008823725.1__PREDICTED_homeobox_protein_NANOG_Nannospalax_galili

MSVDPTDLQTLPSFEKASNSGDSSPTLPSYGPEENDSPLQTPTAEMLNKETASPPSSVDSPDSSTSPTPKLAGSSSEESPGKKEESKVQIKKQKMPMVFSQAQLCTLNERFQKQKYLSLQQMQELSSILNLTYKQVKTWFQNQRMKCKRWQKSNWPKTFNSVRKEMAIKAEKPR

>gi_589960461_ref_XP_006992807.1__PREDICTED_homeobox_protein_NANOG_Peromyscus_maniculatus_bairdii

MSVDLRGSHSLPCKDEAANSGDSSPMPEVHGPEENGCSQVAAEMLQKEIASPCPSSVDLPRQDSPDSSTRPKLKLTGPESEEDAEKEDNKVHTKKQKMRTVFSQAQLCALRERFQRQRYLNLQQMQELSAILNLSYKQVKTWFQNQRMKCKRWQKSQWQKSNGVTQKGSAFVEYPSLHSSYPQGCLNTSGSPSVWDSQTLTDTWSYQTWTNPTSSNQTWTNSACSNQALTSLAWSNQALTDPDWSTQVWNTQSLYSQAWNGQAWNTPFHNFGDDSLQPYMQYQQTFSAGDVGASLAATGGSHVYFSTPPGL

>gi_110625918_ref_NP_082292.1__homeobox_protein_NANOG_isoform_1_Mus_musculus (Used in Figure 1C, mammal NANOG group)

MSVGLPGPHSLPSSEEASNSGNASSMPAVFHPENYSCLQGSATEMLCTEAASPRPSSEDLPLQGSPDSSTSPKQKLSSPEADKGPEEEENKVLARKQKMRTVFSQAQLCALKDRFQKQKYLSLQQMQELSSILNLSYKQVKTWFQNQRMKCKRWQKNQWLKTSNGLIQKGSAPVEYPSIHCSYPQGYLVNASGSLSMWGSQTWTNPTWSSQTWTNPTWNNQTWTNPTWSSQAWTAQSWNGQPWNAAPLHNFGEDFLQPYVQLQQNFSASDLEVNLEATRESHAHFSTPQALELFLNYSVTPPGEI

>gi_198041502_ref_NP_001094251.1__homeobox_protein_NANOG_Rattus_norvegicus

MSVDLSGPHSLPSCEEASNSGDSSPMPAVHLPEENYSCLQVSATEMLCTETASPPPSSGDLPLQDSPDSSSNPKLKLSGPEADEGPEKKEENKVLTKKQKMRTVFSQAQLCALKDRFQRQRYLSLQQMQDLSTILNLSYKQVKTWFQNQRMKCKRWQKNQWLKTSNGLTQKGSAPVEYPSIHCSYSQGYLMNASGNLPVWGSQTWTNPTWNNQTWTNPTWSNQTWTNPTWSNQAWSTQSWCTQAWNSQTWNAAPLHNFGEDSLQPYVPLQQNFSASDLEANLEATRESQAHFSTPQALELFLNYSVNSPGEI

>gi_532044832_ref_XP_005365227.1__PREDICTED_homeobox_protein_NANOG_Microtus_ochrogaster

MKVDPPGPHSLPCCDEAEAVNDGDLSPMPEVHDPEENNSCFQVSVAEKLQKENGSPCPFSVDPPPQDSSDAPNSPITLPAPESEEGTENKAEDKVHPRKQKIRTVFSDTQLCALRDRFQRQRYLSLQQMQELSAILNLSYKQVKTWFQNQRMKCKRWQNNQWPRTGNSVTQKGSAFVEYPSLNSSYPQGYLNTSRSLPVWGSQTWTNTWSSQTWTNPTWSKQAWNNQSWCSQAWSSPSWNTPFHNFGEESLQPYMQYQQNFPACDVEASLEAPGEGHTYFSTPQGLELLLNYSMNMPGEV

>gi_1032915307_ref_XP_016835052.1__PREDICTED_homeobox_protein_NANOG_isoform_X1_Cricetulus_griseus

MSVDLPGPHSLPCYGEAEAPNFGDSSPLPEVHGPEENYSCLQVSATELLREETASPCPSSVDLSLQDSPDSSTSSKVKLPGPESAEGTEKKGEDKAHVKRQKIRTVFSDTQLCALRDRFQRQKYLSLQQMQELSAILNLSYKQVKTWFQNQRMKCKRWQKNQWTKTNINQLFQKDSAFVEYSSFHSGYLQGFLNTSESLPMWGSQTWTNTWSNQTWTNTTWSKQAWSNETWTNPAWSNQAWTNPTWSNQAWTNQSWCSQACNSQAWNTPFLGEDSLQPYMQCQQNLLASDVEASLEAAGEGHTYFSTPQGLELFLNYSVNTPGE

>gi_880876109_ref_XP_012967757.1__PREDICTED_LOW_QUALITY_PROTEIN_homeobox_protein_NANOG_Mesocricetus_auratus

MSVGPPNPHSLPCCDEKKAPDFGGLSPVPEVHGPEENYSCLQVSAIGMLRKETTSPCPSSVGLPLQDRPDSSTSPKLKHLSPESAEGTEKGEDKVHSKKQKIXTVFSDTQLCALKDRFQGXKYLSLQQTQELSAILNLSYNQVKTWFQNQRMKCKQWQKNQXPNTNSSVTQKGSACVGYSSFHSSYPQGSLNTXLPMWGSQTWTNTWSNQTXTNPTWSKQAWSNPTWSNQARANKPWCPQVCNSEAWNTPSGEDSLQLYIQCQKNLLANDVEPSLETAGEGHTYFSTPQGFKLLLNYSMNTLGEM

>gi_617661641_ref_XP_007537597.1__PREDICTED_homeobox_protein_NANOG_Erinaceus_europaeus

MSVDNACPRIPPCPESCSSGGSSPKPEIHSSEEKQAFVHESSEAAHLETASPLPSYMDLPAHESPDSSTSPKAKMLPSEEKSTTMEPELPVPPPVKKQKSRTVFSQSQLCVLNDRFQKQKYLSLQQMQELSTTLNLSYKQVKTWFQNQRMKSKRWLKNSSITQVSGPLEYPSFYPYPHPQGCLVNTPENLPMWNIPNWNNPPWTNQPWNPSQNWYSHAWNNQTWDNQLWPDPAYTCGEESPPLLTQFQQHFSPSDFDTIWDPSGESYSYTQQTGYFNTEQAMDFFLNCPPNTLSQDV

>gi_505830837_ref_XP_004610710.1__PREDICTED_homeobox_protein_NANOG_Sorex_araneus

MSADLECTANLPSSEEPEFRDSSPRPHTCEMEEKDEALPTPSAEEKNEASPTPSAETPPLETVSPLPPAEDLPVQDSPDSSTSPKAEVLPISEESPVKKEDPQGPGKKPKSRTVFSQSQLCVLNERFQRQKYLSLQQMQELSRILNLSYKQVKTWFQNQRMKYKRWQKNSWPKNSDSLAQMTSEYPAGLCCHQGCLVNAPESLPAWGGQPWNSAAAWGGQAAWGAQSWSGQFPGYEDPQQPALSALPQNPPAGDLESTLESPGEAQNLLQQTLKYFSTQQMLDLFPNLPTNMPLGDS

>gi_928173559_ref_XP_013963893.1__PREDICTED_homeobox_protein_NANOG_Canis_lupus_familiaris (Used in Figure 1C, mammal NANOG group)

MPEVYGPRGNPASLPMSSAETPHAETVSPLPSSMDLLTQDSPDSSTSPRVKLPPTSGEERTARKEDATQGKKQKMRTVFSQTQLYVLNDRFQRQKYLSLQQMQELSNILNLSYKQVKTWFQNQRMKSKRWQKSNWPKESNSVTQGYLLNPSGNLPLWSSQAWNNPNWSSQTWNSQSWSSHSWNSQTWCPQAWNNQAWNNPLHNCEEESLQPPIQFQQNSMGDLESIFETAGESHGVLQQSTKYFSTPQIMDFFPNYSEHST

>gi_291045284_ref_NP_001166913.1__homeobox_protein_NANOG_Felis_catus (Used in Figure 1C, mammal NANOG group)

MNTDPAQPQCPPCPEAPDSRDSSPVPEIDGPEENYAPLRMSSAETPHTETVSPLPSCMDLLAQGSPDSSTSPRVKVLPTSAEEITAKKDDPAQGKKQKIRTVFSQTQLYVLNDRFQRQKYLSLQQMQELSNILNLSYKQVKTWFQNQRMKCKRWQKNNWPKNNNTVSQNSSANPEYPGFYSYHQGYLMNTSGNLPIWGNQTWNSQSWSNQTWNSQSWSNQTWNSQSWSNQTWNSQTWCPQAWNGQGWNSQLHDCGEESPQPQIQLQQNSVSDLQSILETTGESHSVIQQTAKYFSAQQIMDLFPNYPEHTA

>gi_591327332_ref_XP_007089616.1__PREDICTED_homeobox_protein_NANOG_Panthera_tigris_altaica

MNTDPAQPRCPPCPEAPDSRDSSPVPEIDGPEENYAPLQMSSAETPHTETVSPLPSCMDLLVQDSPDSSTSPRVKVLPTSAEEITAKKDDPAQGKKQKIRTVFSQTQLYVLNDRFQRQKYLSLQQMQELSNILNLSYKQVKTWFQNQRMKCKRWQKNNWPKNNNTVTQNSSANPEYPGFYSYHQGYLMNTSGNLPIWGNQTWNSQSWSNQTWNSQSWSNQTWNSQTWCPQAWNGQAWNSQLHDCGEESPQPQIQLQQNSVSDLQSLLETTGESHSVIQQTAKYFSAQQIMDLFPNYPEHTA

>gi_961713812_ref_XP_014917042.1__PREDICTED_LOW_QUALITY_PROTEIN_homeobox_protein_NANOG_Acinonyx_jubatus

MNTDPAQPQCPPCPEAPDSRDSSPVPEIDGPEENYAPLQMSSAETPHTETVSPLPSCMDLLVQDSPDSSTSPRVKVLPTSAEEITAKKDDPAQGKKQKIRTVFSQTQLYVLNDRFQRQKYLSLQQMQELSNILNLSYKQVKTWFQNQRMKCKRWQKNNWPKNNNTVTQNSSANPEYPGFYSYHQGYLMNTSGNLPIWGNQTWNSQSWSNQTGTASRGATRPNSQSWSNQTWNSQTWCPQAWNGQAWNSQLHDCGEESPQPQIQLQQNSVSDLQSILETTGESHSVIQQTAKYFSAQQIMDLFPNYPEHTA

>gi_511894037_ref_XP_004766658.1__PREDICTED_homeobox_protein_NANOG_isoform_X1_Mustela_putorius_furo

MSADPAPPQCRSGPRPPGSRDSSPMPEVCRHEENYAFLQMSSAETPHAETVSPLPSSVDLLVQDSPDSSTSPKVKLPPTSAEERTGRREDTAQGKKQKIRTVFSQTQLYVLNDRFQRQKYLSLQQMQELSNILNLSYKQVKTWFQNQRMKCKRWQKNHWPKESKSVTQNSTAATEYPAFYSYHQGHLMNTSANLPIWSSQTWNNPNWSNQTWNSQSWSNHSWNGQSWSNHSWTSQTWCPQAWNSQLHSCGEESLQPQIQFQQNSVSDLESLLETSGESYSVIQQSAKYFSTQQIMDLFPNYSVNIQPEDV

>gi_585172740_ref_XP_006738313.1__PREDICTED_homeobox_protein_NANOG_Leptonychotes_weddellii

MSADPALPLCPPGPEAPSSRDSSPMPEIYGPEENYASLQMSSAETPHAETVSPLPSSMDLLVQDSPDSSTSPRVKPPPTSGEERTVRKEDMAQGKKQKIRTVFSQTQLYVLNDRFQRQKYLSLQQMQELSNILNLSYKQVKTWFQNQRMKCKRWQKNHWPKESKSVSQNSTPTTEYPGFYSYHQGYLRNTSGNLPIWSNQTWNNPNWSNQTWNSQSWSNHSWNGEC

>gi_752435901_ref_XP_011235450.1__PREDICTED_LOW_QUALITY_PROTEIN_homeobox_protein_NANOG_Ailuropoda_melanoleuca

MSVDPAPPQCPPGPEAPSSRDSNPMPEIYGPEENYASLQMSPAETLHTETVSPPPSSMDLLVQDSPDSSTSPRGKLPPTSVEERTVRKEDTTQGKKQKIRTVFSQTQLYVLNDRFQRQKYLSLQQMQELSNILNLSYKQVKTWFQNQRMKCKRWQKNHWPKESKSVTQNSAATVEYPGFYSYHQGYLMNTSGNLPIWSNQTWNSQSWSNHSWNGQAWSNHSWTTQTWCPQAWNSQPCNSQLHSCGEESLXPQIQFQPTSVSNVESILETAGESHSVIQQSAKYFSTQQIMDLFPNYSVNIQPEDV

>gi_671039538_ref_XP_008681710.1__PREDICTED_homeobox_protein_NANOG_isoform_X1_Ursus_maritimus

MSVDPAPPQCPPGPEAPSSRDSNPMPEVYGPEENYASLQMSPAETPHTETVSPPPSSMDLLVQDSPDSSTSPRGKLPPTSVEERTVRKEDTAQGKKQKIRTVFSQTQLYVLNDRFQRQKYLSLQQMQELSNILNLSYKQVKTWFQNQRMKCKRWQKNHWPKESKSVTQNSTATAEYPGFYSYHQGYLMNTSGNLPIWSNQTWNSQSWSNHSWNGQAWSNHSWNGQAWSNHSWTSQTWCPQAWNSQPCNSQLHSCGEESLQPQIQFQQNSVSDLESILETSGESHSVIQQSAKYFSTQQIMDLFPNYSVNIQPEDV

>gi_507710605_ref_XP_004717486.1__PREDICTED_homeobox_protein_NANOG,_partial_Echinops_telfairi

MSVDVASPQNLLEASDSKNSSPVTTSLGAKEDGTSLQMSSAEPPYSETARSPLPSSVDLLLQKSPDSSTSPKVQLPASAETDAARKESKGQGKKQKVRTVFSQTQLCILKDHFQKQKYLSLQQMQELSEVLNLSYKQVKTWFQNQRMKCKRWQKTTNWSKNNHAVTQVSAPTEYLDVCASYHQGCLPSGNLPMWSNQSWNSQAWNNQSWNSQAWNN

>gi_586490173_ref_XP_006875553.1__PREDICTED_homeobox_protein_NANOG_Chrysochloris_asiatica

MSVADLISQSLPEACDSRVSSPLSTILEAEKNDISLQMSSVEALYSDTVSPLPASIGLLLQDSPDSSTSPKVKLPTSIEKSTMKEGKGLGKKQKVRTVFSQMQLCILKDRFQKQKYLSLQQMQELSEVLNLSYKQVKTWFQNQRMKCKRWQKNTKWPNSSNGVAQKVSVPAEYLDLCASYHQGYLPSGNLPMWSNQTWSSQTWSNQTWSSSQAWCTQAWNNQSWNTQAWNNQSQICGEEFLLPQFQQNSASDLEASQTIDLFLNDSMNIQPEEV

>gi_344278136_ref_XP_003410852.1__PREDICTED_homeobox_protein_NANOG_Loxodonta_africana (Used in Figure 1C, mammal NANOG group)

MSVDLASPQSLPEACDSRDTSPISAILEAEENYTSLQMSSAEALYSETASPLPSSTDGLIQDSPDSSTSPQIKPSTSALVGKSTVKKEGKGQGKKQKVRTVFSQMQLCILKDRFQKQKYLSLQQMQELSEALNLTYKQVKTWFQNQRMKCKRWQKNTNWSKTSNSVTQKVSVPIEFLDLYSSSHQGCLPSGNLSMWSNQTWSNQTWNSQSWSNHSWNSQAWSNHSWNSQAWSNHSWNSQSSWNSQSSWNNQVWNNQFQNCGEEFLLPQVQFQQNSVSDLETSQTIDLFLNYSMNIQPEDVVTMGI

>gi_471411564_ref_XP_004387335.1__PREDICTED_homeobox_protein_NANOG_Trichechus_manatus_latirostris

MSVDLASPQSLPEACDSRDSSPISTILGAEENYTSLQMSSAEALYSETVSPLPSSMDQLIQDSPDSSTSPKIKSPASASIEKSTVKKEGKGQGKKQKVRTVFSQTQLCILKDRFQKQKYLSLQQMQELSEVLNLSYKQVKTWFQNQRMKCKRWQKNTNWSKNSSGAIQKVSVPTEFLDLYSSYHQGCFPSGNLPMWNNQTWNNSTWSNQTWNSQSWNNHSWNSQAWSNHSWNSQAWCTQSWNSQSPWNNQFQNCGEDFLLPQIQFQQNSASDLETSQTIDLFLNYSMNIQPEDA

>gi_504173922_ref_XP_004596526.1__PREDICTED_homeobox_protein_NANOG_Ochotona_princeps

MSVAAACPQRLPALEGPDATGSSPAPASPGPEENHPPLQMTSAGTPHTDPATQLLSSAGLLIQDSPDSSTSPEVKPLVSVENGAKPEEKVKKQKIRTVFSQDQLCVLHDRFQRQKYLSLQQMQELSSILNLTYKQVKTWFQNQRMKCKKWQRKNLPNNMIQKGCATAEYPGLCSPYQQGCLVNTPENLPVWSSNQTMWGNQTWNNPAWNNPSWSSPSWDSQSWYPQTWNSPLLNCGEEMLQPCVQFSQTCPATEFDSVLETAGESLNTVPPAPKHLGTPQALDLLLSYPVDMQPDDL

>gi_830180351_ref_XP_012587824.1__PREDICTED_homeobox_protein_NANOG_isoform_X2_Condylura_cristata

MSVGPVCSQSLPCPETSTSRASSPMPEVYEAEEDYSSLPKSSAEMPHTENVSPLPSSMDLLIQDSPDSSTSPRVKLCSTSEENSTEKKEERIPVKKQKSRTVFSQTQLCVLNDRFQRQKYLSLQQMQELSNLLNLSYKQVKTWFQNQRMKCKRWQKSSWPKSGMTQGCLVNTPEDLPIWSNQPWSNPTWGSQSWSQPWCTQPWNNQPWNHQAWNSPLHSSGEDPFSPLLQLQQNFSLSDLEATLEICGENHNIIQPPSKYFSTQQIMDLFPTYSMNAQSEDV

>gi_478529088_ref_XP_004438717.1__PREDICTED_homeobox_protein_NANOG_isoform_X1_Ceratotherium_simum_simum

MSVDPALPQSLPCPEASNSGELSPMPEIYEPEENYASLQLSSAETPHMETVSPLPSSMDLLIQDSPDSSTSPRVKLLPTSVEKSTVKKEEKVQAKKQKIRTVFSQTQLCVLNDRFQRQKYLSFQQMQELSNLLNLSYKQVKTWFQNQRMKCKRWQKNNWPKTCNSVTQKGSATTEYPGLYSYHQGSLVNTAGNLPMWSNQTWNNPTWSSQTWNSQSWSNHSWSSQTWCSQAWNNQAWNNQAWNNQAWNSQFHSCGEEFLQPQIQFQQNSLVSGLEATLETAGESHTVTQQTAKYFSTQQIMDLFPNYSMNIQPEDV

>gi_149712673_ref_XP_001498858.1__PREDICTED_homeobox_protein_NANOG_isoform_X1_Equus_caballus (Used in Figure 1C, mammal NANOG group)

MSVDPALPQSLPCPEASNSRGSSPVPEIYGPEENYASLQMSSAETPHMETVSPLPSSMELLIQDSPDSSTSPRVKPLPASAETSEAKKEEKVHGKKQKIRTVFSQTQLCVLNDRFQRQKYLSLQQMQELSNILNLSYKQVKTWFQNQRMKCKRWQKNNWPKTSNSVTQGSATTDYPGLYSYHQGCLVNTAGNVPMWSHQAWNNPAWSNQTWNSQSWSNHSWNSQTWCSQAWNNQAWNNQAWNSPFHSCGEDFLQPQIQFQQNSPVSDLEATLETAGESHNATQQTAKYFSTQQIMDLFPNYPMNIQPEDM

>gi_664739579_ref_XP_008527088.1__PREDICTED_homeobox_protein_NANOG_isoform_X1_Equus_przewalskii

MSVDPALPQSLPCPEASNSRGSSPVPEIYGPEENYASLQMSSAETPHMETEKSVILFPTVSPLPSSMELLIQDSPDSSTSPRVKPLPASAETSEAKKEEKVHGKKQKIRTVFSQTQLCVLNDRFQRQKYLSLQQMQELSNILNLSYKQVKTWFQNQRMKCKRWQKNNWPKTSNSVTQGSATTDYPGLYSYHQGCLVNTAGNVPMWSHQAWNNPAWSNQTWNSQSWSNHSWNSQTWCSQAWNNQAWNNQAWNSPFHSCGEDFLQPQIQFQQNSPVSDLEATLETAGESHNATQQTAKYFSTQQIMDLFPNYPMNIQPEDM

>gi_560992636_ref_XP_006218448.1__PREDICTED_homeobox_protein_NANOG_isoform_X1_Vicugna_pacos

MSADADCPQSLLCPEASNSRETSPMPEPYGPEEDYTSLQMSSAETPDTETVSPLPSSMDLLIQDSPDSSTSPRVKPLPTSAESSTEKQEEKVQAKKQKVRTVFSQTQLCVLNDRFQRQKYLSLQQMQELSNILNLSYKQVKTWFQNQRMKCKRWQKNNWPRNSSSMTQGSATTEYPGLYSYHQGCLANTSGNLPMWGNQTWNSPTWNSQTWNSQSWGSHSWNSQPWCPQAWNNQTWNNQAWNSQFNNCVEEFLQPHSQLQQNSPFSDLEAALESAGESHNVIQQAAKYVSPQQQIMDLFPSYSINMQPEDV

>gi_560932180_ref_XP_006192214.1__PREDICTED_homeobox_protein_NANOG_isoform_X1_Camelus_ferus

MSADADCPQSLLCPEASNSRETSPMPEPYGPEEDYTSLQMSSAETADTETVSPLPSSMDLLIQDSPDSSTSPRVKPLPTSAESSTEKQEEKVQVKKQKVRTVFSQTQLCVLNDRFQRQKYLSLQQMQELSNILNLSYKQVKTWFQNQRMKCKRWQKNNWPRNSSSVTQGSATTEYPGLYSYHQGCLANTSGNLPMWGNQTWNSPTWNSQTWNSQSWGSHSWNSQPWCPQAWNNQTWNNQAWNSQFSNCVEEFLQPHIQLQQNSPFSDLEAALESAGESHNVIQQAAKYVSPQQQIMDLFPSYSMNMQPEDV

>gi_744596718_ref_XP_010989109.1__PREDICTED_homeobox_protein_NANOG_isoform_X1_Camelus_dromedarius

MSADADCPQSLLCPEASNSRETSPMPEPYGPEEDYTSLQMSSAETADTETVSPLPSSMDLLIQDSPDSSTSPRVKPLPTSAESSTEKQEEKVQVKKQKVRTVFSQTQLCVLNDRFQRQKYLSLQQMQELSNILNLSYKQVKTWFQNQRMKCKRWQKNNWPRNSSSVTQGSATTEYPGLYSYHQGCLANTSGNLPMWGNQTWNSPTWNSQTWNSQSWGSHSWNSQPWCPQAWNNQTWNNQAWNSQFSNCVEEFLQPHIQLQQNSPFSDLEAALESAGESHNVIQQAAKYVSPQQQIMDLFPSYSMNMQPEDV

>gi_743702886_ref_XP_010944669.1__PREDICTED_homeobox_protein_NANOG_isoform_X1_Camelus_bactrianus

MSADADCPQSLLCPEASNSRETSPMPEPYGPEEDYTSLQMSSAETADTETVSPLPSSMDLLIQDSPDSSTSPRVKPLPTSAESSTEKQEEKVQVKKQKVRTVFSQTQLCVLNDRFQRQKYLSLQQMQELSNILNLSYKQVKTWFQNQRMKCKRWQKNNWPRNSSSVTQGSATTEYPGLYSYHQGCLANTSGNLPMWGNQTWNSPTWNSQTWNSQSWGSHSWNSQPWCPQAWNNQTWNNQAWNSQFSNCVEEFLQPHIQLQQNSPFSDLEAALESAGESHNVIQQAAKYVSPQQQIMDLFPSYSMNMQPEDV

>gi_594092986_ref_XP_006069824.1__PREDICTED_homeobox_protein_NANOG_isoform_X1_Bubalus_bubalis

MSVGPACPQSLLGPKASNSRESSPMPEESYASLQTSSADTLDTDTVSPLPSSMDLLIQDSPDSSTSPRVKPLSPSVEESTEKEETVPVKKQKIRTVFSQTQLCVLNDRFQRQKYLSLQQMQELSNILNLSYKQVKTWFQNQRMKCKKWQKNNWPRNSNGMPQGPAMAEYPGFYSYHQGCLVNSPGNLPMLGNQTWNNPTWSNQSWNSQSWSNHSWNSQAWCPQAWNNQPWNNQFNNYMEEFLQPGIQLQQNSPVCDLEATLGTAGENYNVIQQAVKYFNPQQQITDLFPNYPLNIQPEDL

>gi_555993218_ref_XP_005908374.1__PREDICTED_homeobox_protein_NANOG_isoform_X1_Bos_mutus

MSVGPACPQRLLGPEASNSRESSPMPEESYVSLQTSSADTLDTDTVSPLPSSMDLLIQDSPDSSTSPRVKPLSPSVEESTEKEETVPVKKQKIRTVFSQTQLCVLNDRFQRQKYLSLQQMQELSNILNLSYKQVKTWFQNQRMKCKKWQKNNWPRNRNGMPQGPAMAEYPGFYSYHQGCLVNSPGNLPMWGNQTWNNPTWSNQSWNSQSWSNHSWNSQAWCPQAWNNQPWNNQFNNYMEEFLQPGIQLQQNSPVCDLEATLGTAGENYNVIQQTVKYFNSQQQITDLFPNYPLNIQPEDL

>gi_70778752_ref_NP_001020515.1__homeobox_protein_NANOG_Bos_taurus (Used in Figure 1C, mammal NANOG group)

MSVGPACPQSLLGPEASNSRESSPMPEESYVSLQTSSADTLDTDTVSPLPSSMDLLIQDSPDSSTSPRVKPLSPSVEESTEKEETVPVKKQKIRTVFSQTQLCVLNDRFQRQKYLSLQQMQELSNILNLSYKQVKTWFQNQRMKCKKWQKNNWPRNSNGMPQGPAMAEYPGFYSYHQGCLVNSPGNLPMWGNQTWNNPTWSNQSWNSQSWSNHSWNSQAWCPQAWNNQPWNNQFNNYMEEFLQPGIQLQQNSPVCDLEATLGTAGENYNVIQQTVKYFNSQQQITDLFPNYPLNIQPEDL

>gi_742162450_ref_XP_010847659.1__PREDICTED_homeobox_protein_NANOG_isoform_X1_Bison_bison_bison

MSVGPACPQSLLGPEASNSRESSPMPEESYVSLQTSSADTLDTDTVSPLPSSMDLLIQDSPDSSTSPRVKPLSPSVEESTEKEETVQVKKQKIRTVFSQTQLCVLNDRFQRQKYLSLQQMQELSNILNLSYKQVKTWFQNQRMKCKKWQKNNWPRNSNGMPQGPAMAEYPGFYSYHQGCLVNSPGNLPMWGNQTWNNPTWSNQSWNSQSWSNHSWNSQAWCPQAWNNQPWNNQFNNYMEEFLQPGIQLQQNSPVCDLEATLGTAGENYNVIQQTVKYFNSQQQITDLFPNYPLNIQPEDL

>gi_936975633_ref_NP_001301200.1__Nanog_homeobox_Capra_hircus

MSVDPACPQSLLGPEASNSGESSPMPEESYASLQMSSADTLDTDTVSPLPSSMDLLIHDNPDSSTSPRVKPLSPSAEESTEKEEKVPVKKQKIRTVFSQTQLCVLNDRFQRQKYLSLQQMQELSNILNLSYKQVKTWFQNQRMKCKKWQKNNWPRNSNDVPQDPATAEYPSFYSYHQGCLVNSPRNMPMWGNQTWNNPTWSNQNWNSQSWSNHSWNSQAWCPQAWNNQPWNNQCNNYMEEFLQPGIQLQQNSPVCDLEATLGTAGENYNVIQQAVKYFSSQQQITDLFPNYPLNIQPEDL

>gi_965928541_ref_XP_004006950.3__PREDICTED_homeobox_protein_NANOG_isoform_X1_Ovis_aries

MSVDPACPQSLLGPEASNSGESSPMPEESYASLQMSSADTLDTDTVSPLPSSMDLLIQDSPDSSTSPRVKPLSPSAEESTEKEEKVPVKKQKIRTVFSQTQLCVLNDRFQRQKYLSLQQMQELSNILNLSYKQVKTWFQNQRMKCKKWQKNNWPRNSNGVPQGPATAEYPGFYSYHQGCLVNSPGNLPMWGNQTWNNPTWSNQSWNSQSWSNHSWNSQAWCPQAWNNQPWNNQFNNYMEEFLQPGIQLQQNSPVCDLEATLGTAGENYNIIQQTVKYFSSQQQITDLFPNYPLNIQPEDL

>gi_556772569_ref_XP_005981449.1__PREDICTED_homeobox_protein_NANOG_Pantholops_hodgsonii

MSVDPACPQSLLGPEASSSGESSPMPEESYASLQMSSADNLDTDTVSPLPSSMDLLIQDSPDSSTSPRVKPLSPSAEESTEKEEKVPIKKQKIRTVFSQTQLCVLNDRFQRQKYLSLQQMQELSNILNLSYKQVKTWFQNQRMKCKKWQKNNWPRNSTGVPQGPATAEYPGFYSYHQGCLVNSPGNLPMWGNQTWNNPTWSNQSWNSQSWSNHSWNSQAWCPQAWNNQPWNNQFNNYMEEFLQPGIQLPQNSPVCDLEATLGTAGENYNVIQQTVKYFSSQQQITDLFPNYPLNIQPEDL

>gi_194018700_ref_NP_001123443.1__homeobox_protein_NANOG_Sus_scrofa

MSVDPACPQSLLCPEASISSESSPMPEVYGPEENYASLQMSSAETLDTETVSPLPSSMDLLIQDSPDSSTSPRVKPLPTSAEKSTEKEEKVPVKKQKIRTVFSQTQLCVLNDRFQRQKYLSLQQMQELSNILNLSYKQVKTWFQNQRMKCKRWQKNHWPRNSNSVIQGSASTEYPGFYSYHQGCLVNASGNLPVWGNQSWSNPTWSNQTWNSQSWSNQTWNSQTWCPQAWNNQTWNSQLNNYVEEFLQPQLQFQQNSISDLEAVLETAGENHNVIQQTSKYCGTQQQIMDLFPNYSMNIQPEDM

>gi_594698097_ref_XP_007196184.1__PREDICTED_homeobox_protein_NANOG_Balaenoptera_acutorostrata_scammoni

MSVDPACPQSLRGPEASNSRESSPMPEIYGPEENYVSLQMSSVETHDMETVSPLPSFSMDLLIQDSPDSSTSPRVKLLATAADKSTEKKEEKVLIKKQKTRTVFSQTQLCVLNDRFQRQKYLSLQQMQELSNILNLSYKQVKTWFQNQRMKCKRWQKNNWPRNSNTVTQGPATTEYPGFYSYHQGCLANSSGNLPMWGNQTWNNPTWSNQSWNSQSWSNHPWNNQTWCPQAWNNQTSNNQFNNYVEEFLQPQTQFQQNSPVSDLEATLETAGESYNIIQQTAKYFNSQQQIMDLFPNYSLNIQPEDL

>gi_466054927_ref_XP_004279145.1__PREDICTED_homeobox_protein_NANOG_isoform_X1_Orcinus_orca

MSVDPACPQSLRGPEASNSRESSPVPEIYGPEENYVSLQMSSAESHDMETVSPLPSFSMDVLIQDSPDSSTSPRVKLLATAADKSTEKKEEKVLVKKQKTRTVFSQTQLCVLNDRFQRQKYLSLQQMQELSNILNLSYKQVKTWFQNQRMKCKRWQKNNWPRNSNIVTQGPATTEYPGFYSYHQGCLVNSSGNLPMWGNQTWNNPTWSNQSWNSQSWNNQSWNSQTWCPQAWNNQTWNNQFNNYVEEFLQPQIQYQQNSPVSDLEATLETAGESYNIIQQTAKYFNSQQQIMDLFPNYSLNIPPEDL

>gi_602718510_ref_XP_007469435.1__PREDICTED_homeobox_protein_NANOG_Lipotes_vexillifer

MSVDPACPQSLRGPEASNSRESSPMPEIYGPEENYVSLQMSSAETHDMETVSPLPSFSMDLLIQDSPDSSTSPRVKLLATAADKSTEKEEKVLVKKQKTRTVFSQTQLCVLNDRFQRQKYLSLQQMQELSNILNLSYKQVKTWFQNQRMKCKRWQKNNWPRNSNIVTQGPATTEYPGFYSYHQGCLVNSSGNLPMWGNQTWNNPTWSNQSWNSQSWNNQSWNSQTWCPQAWNNQTWNNQFNNYVEEFLQPQIQYQQNSPVGDLEATLETAGESYNIIQQTAKYFNSQQQIMDLFPNYSLNIQPEDL

>gi_1012271826_ref_XP_015983542.1__PREDICTED_homeobox_protein_NANOG_isoform_X1_Rousettus_aegyptiacus

MSVDPAGSQSSPCPEASSSREPSPVPDIYGSEENYASLQMSSAETLNMETVSPLPSNMDLLIQNSPDSSTSPNIILPTFVENSTVKKEEKVQIKKQKIRTVFSQTQLCVLNERFQRQKYLSLQQMQELSNILNLSYKQIKTWFQNQRMKCKRWQKYNWPKNSNCAVQGSTTPEYPGLSSYHQGCLVNASGNLSMWSSNQTCNNPTWSYHSWNSQTWCPQAWNNQAWNNQLYSCGEEALQPQIQFQQNRPVSDLEATLEMAGESYDVIQQIPKYCSVQQSMDLFPNYFMNVQPEDV

>gi_586562969_ref_XP_006914738.1__PREDICTED_homeobox_protein_NANOG_isoform_X1_Pteropus_alecto

MSVDPACSQSSPCPEASSSREPSPVPDIYGPEGNYASLQMSSAETLNTETVSPLPSNMDLLIQNSPDSSTSPNIILPTFVENSTVKKEEKVQIKKQKIRTVFSQTQLCVLNERFQRQKYLSLQQMQELSNILNLSYKQIKTWFQNQRMKCKRWQKYNWPKNSNCAMRKGSATTEYPGLSSCHQGCLVNASGNLSMWSNQTCSNPTWSYHSWNSQTWCPQAWNNQAWNNQLYSCGEEALQPQIQFQQNRPVSDLEATLEMAGESYDVIQQIPKYCSVQQTMDLFPNYFMNVQPEDV

>gi_759136379_ref_XP_011364079.1__PREDICTED_homeobox_protein_NANOG_Pteropus_vampyrus

MSSAETLNTETVSPLPSNMDLLIQNSPDSSTSPNIILPTFVENSTVKKEEKVQIKKQKIRTVFSQTQLCVLNERFQRQKYLSLQQMQELSNILNLSYKQIKTWFQNQRMKCKRWQKYNWPKNSNCAMRKGSATTEYPGLSSCHQGCLVNASGNLSMWSNQTCSNPTWSYHSWNSQTWCPQAWNNQAWNNQLYSCGEEALQPQIQFQQNRPVSDLEATLEMAGESYDVIQQIPKYCSVQQTMDLFPNYFMNVQPEDV

>gi_1016714809_ref_XP_016077207.1__PREDICTED_homeobox_protein_NANOG_Miniopterus_natalensis

MSVDSACPQSLPCSELSNSREPSPAPEIYEPEESYASLQMSSAETLHTETVSPLPSSTDLLIQNSPDSSTSPRVILPASGESSAGKGEKAQAKKQKIRTVFSQTQLCVLNERFQRQKYLSLQQMQELSTILNLSYKQIKTWFQNQRMKCKRWQKYNWPKNSNRMPQMSSATTEYLGLSSCHQGCLVNASGNLSVWSNQTCGNLPWSNHSWNSPSWNYHSWNSQTWCPQAWNNQAWNNQFYSYGEESLQPQIQFQQNPVSELEATLETAGETYDAILQTAKYCNTQQTADLFPDYSMNMQPDSTNIQP

>gi_641700941_ref_XP_008139043.1__PREDICTED_homeobox_protein_NANOG_Eptesicus_fuscus

MSVDSAYPQSLPCSESSDSREPSPVPEIYEPEENYASLQMSSAETLHTETVSSLPSSMDLLIQNSPDSSTSPSSPSATLPTSVENRTGKEEKAQVKKQKIRTVFSQTQLCVLNERFQRQKYLSLQQMQELSSILNLSYKQIKTWFQNQRMKCKRWQKYNWPKNSHRMTQMSSATTEYLGLSSCHQGCLVNTSGNLSIWSNPSWTSPSWNYPAWNGQTWCPQAWNNQAWNNQAWNNQFCSYGEESLQPQIQFQQNPVSDLEASLEMAGEAYDAILQAPKYYGTQQSADLFPDFSMNIV

>gi_987946193_ref_XP_015419562.1__PREDICTED_LOW_QUALITY_PROTEIN_homeobox_protein_NANOG_Myotis_davidii

MSVDSAYPQSLPCSEPSNSREPSPVPEMYEPEENYASLPMSSGETPHAETVSPLPSSMDQLIQNRPDSSTSPSPSSPSATPPTSEENGTGKEEKAQVKKQKIRTVFSQTQLCVLNERFQRQKYLSLQQMQELSSILNLSYKQIKTWFQNQRMKCKRWQKYNWPKNSHRVTQMSSATTDYLGLSSCHQGCLVNTSGNLSICSNHSWTSPSWNYPAWNGQTWCPQAWNNQAWNNQAWNNQLLSYGEEALQPQIQFQQNPVSDLEASLEIAGETYDAILQAPKYYGTQQSTDLFPDYSMSIXPESTNT

>gi_946754953_ref_XP_014392335.1__PREDICTED_LOW_QUALITY_PROTEIN_homeobox_protein_NANOG_Myotis_brandtii

MSVDSAYPQSLPCSESSNSREPSPVPEIYEPEENYASLQMSSAETLHTETVSPLPSSMDQLIQNSPDSSTSPSPSSPSATPPTSVENGTGKEEKAQVKKQKIRTVFSQTQLCVLNERFQRQKYLSLQQMQELSSILNLSYKQIKTWFQNQRMKCKRWQKYNWPKNSHRVTQMSSATTDYLGLSSCHQGCLVNASGNLSMWSNHSWTSPSWNYPAWNGQIWCPQAWNNQAWNNQAWNNQFLSYGEEALQPQIQFQQNPVSDLEASLEIAGETFDAILQAPKYYGTQQSTDLFPDYSMSIXPESTNT

>gi_940717893_ref_XP_014307231.1__PREDICTED_LOW_QUALITY_PROTEIN_homeobox_protein_NANOG_Myotis_lucifugus (Used in Figure 1C, mammal NANOG group)

MSVDSAYPQSLPCSESSNSREPSPVPEIYEPEENYASLQMSSAETLHTEAVSPLPSSMDQLIQNSPDSSTSPSPSSPSATPPTSVENGTGKEEKAQVKKQKIRTVFSQTQLCVLNERFQRQKYLSLQQMQELSSILNLSYKQIKTWFQNQRMKCKRWQKYNWPKNSHRVTQMSSATTDYLGLSSCHQGCLVNASGNLSMWSNHSWTSPSWNYPAWNGQTWCPQAWNNQAWNNQAWNNQFLSYGEEALQPQIQFQQNPVSDLEASLEIAGETYDAILQAPKYYDTQQPTDLFPDYSMSIXPESTNT

>gi_984139981_ref_XP_015362048.1__PREDICTED_homeobox_protein_NANOG_isoform_X1_Marmota_marmota_marmota

MSMDPAFPQSLPCLEASDSRVFSPIPVINGPEEIYPPVQMSSAEMPLTETAVSPSPSSMDLLIQDSPDSSTSPKVKPSTSVQESTTKKEDSKIQVKKQKMRTVFSQTQLCILNDRFQKQKYLSLQQMQELSSNLNLSYKQVKTWFQNQRMKCKRWQKNNWPKNSNSVTQGSLPTEYSGPYSNYNQGYQVNSPGNLPPWSNQTWNNPTWSNQTWNSQSWSSQSWNSQSWSNHSWNTQTWYTQSWNNQAWNNSFYNYGEESLQPSMQFQQNFPASDLETTLAFTGESHRYFSTPQSMDLFLNYSLNRQSENM

>gi_914929973_ref_XP_013221338.1__PREDICTED_homeobox_protein_NANOG_Ictidomys_tridecemlineatus

MSMDPAFPQSLPCLEASDSRVFSPIPVINGPEEIYPPVQMSSAEMPLTETVSPSPSSMDLLIQDSPDSSTSPKVKPSTSVQESTIKKEDSKIQVKKQKIRTVFSQTQLCILNDRFQKQKYLSLQQMQELSSNLNLSYKQSVFTYIENEFISRHSGMGLYSQGSLPTEYSGPYSNYNQGYQVNSPGNLPPWSNQTWNNPTWSNQTWNSQSWSSQSWNSQSWSNNSWNTQTWYTQSWNNQAWNNQTWNNSFYNYGEESLQPSMQFQQNFPASDLETTLAITGESHRYFSTPQSMDLFLNYSLNRQSENM

>gi_562834711_ref_XP_006172155.1__PREDICTED_LOW_QUALITY_PROTEIN_homeobox_protein_NANOG_Tupaia_chinensis

MSVDPACPQRLPCSEPLDSMGSPPPPVSCGPEEQDPALPMSSAEVPHAETVSSLPSSVDPLILESPDSSTSPTVDKSSVKKEEKVQVKKQKSRTVFSATQLCVLNARFQRQKYLSLXQMQELSDILNLSYKQVKTWFQNQRMKCKRWQKNKWPKNCSAVTQKGPAPAEYPGIYAAYHQGCPVNPSGNFPGWPAGTPQSWTTQAWSPQSWSTQSWNSQTWCSPAWDAQFYNCGEESLQSSMQFQQVLPAGDLEAILETSGEGQNVLQXTTKYLSTAQTMDFMLNYSMSMLPEDA

>gi_291392835_ref_XP_002712808.1__PREDICTED_homeobox_protein_NANOG_Oryctolagus_cuniculus (Used in Figure 1C, mammal NANOG group)

MSVDPACPASLPASEALDDQDSSLMPVVPEPEGKHPTLQLPCAQVPLTDTASPLPSSMDLPTQESPDSSTSPSPRVVKTPDSAEKSTKKEEKVKKQKIRTVFSQTQLCVLNDRFQRQKYLSLQQMQELSSLLNLSYKQVKTWFQNQRMKCKRWQRNNWPNICNSVTQKGSGPAEYPGFYPAYHQGCLVNTPGSLPMWNNQTWNNQTWNNPAWNSQSWSNHSWDSQAWYSQAWNNQAWNSPFLNCGDEVLQSSMQLQQNCPVSDLESILETVGESQNTAQQTTKHFSASQALDLLLNYSVNMQPENV

>gi_488527008_ref_XP_004455326.1__PREDICTED_homeobox_protein_NANOG_isoform_X1_Dasypus_novemcinctus (Used in Figure 1C, mammal NANOG group)

MSVDLASPQSLSCPKSDSRESSPMPEIYKAEENYASLQISSAEVPQSETVSPLPSPMDLLIQDSPDSSTSPKMKLPAAGENSTAEKEDEVPVKKQKSRTVFSQAQLCILNDRFQRQKYLSLQQMQELSHVLNLSYKQVKTWFQNQRMKCKRWQKNVSWSKSSNSMTQKGLAPTEYLGLYSPYPQGCLVNASANLPMWSNQAWNNQAWNNPTWSSHSWNTQTWCTQAWNNQFQNSGEESLQSQIQFQQNYASDLEAILGSIGESHNVIQQAVKYYSTAQTMDLFPSYYCMNGQPEDM

>gi_829830038_ref_XP_012633070.1__PREDICTED_homeobox_protein_NANOG_Microcebus_murinus

MPNVNLPFCQETQRRALFRLFPAASPLPSSTDLLIQDSPDSSTSPKVRLPALAQKSALKEDKAQVKKQKSRTVFSATQLCVLNDRFQRQKYLSLQQMQELSNILNLSYKQVKTWFQNQRMKSKRWQKNNWSKNVNSLTQFQTASAPTEYPGLYSSYHQACLMNTSGNLPVWSNQTWNNQTWNSSQPWSNHSWNSQTWCSPACNNQAWNNNPFLNCGEETLQSCLQFQQNFLASDLEAALETAGESLNVIQQAAKYFSSPQAMESFLNYSMSMPPEDV

>gi_395847517_ref_XP_003796416.1__PREDICTED_homeobox_protein_NANOG_isoform_X1_Otolemur_garnettii

MSMNPACSQRLPCPESSDSSDCSSMPVICGHEENYPSLQMASTEVPLTETASPLSTSMDLLAQDSPDSSTSPKVKQPTSVEKSVVKKEGKVQVKKQKTRTVFSTTQLCILNDRFQRQKYLSLQQMQELSNILNLSYKQVKTWFQNQRMKSKRWQKNNWLKNTNSMTQKASAPMEYPSFYSSYHQACLMNTSGNLPVWSNQTWNNPTWGNQTWSSQSWSNHSWNSQTWCTQAWNSQAWNNPFFNCGEEPLQPCLQFQQNFLASDLEIALEAAGESHNVIHQTTKYFSSPQTADSFLNYSMNMQPEDV

>gi_826275194_ref_XP_012493250.1__PREDICTED_homeobox_protein_NANOG_Propithecus_coquereli

MNMTASPLPSSVDLLIQDSPDSSTSPKVKLPTSAQNSAVKKEDKAQGKKQKSRTVFSTTQLCILNDRFQRQKYLSLQQMQELSNILNLSYKQVKTWFQNQRMKSKRWQKNNWSKNFNSLTQKASAPTEYPGLCPSYHQACLMNTSGNLPAWSNQTWSNPAWNNQTWNSQPWSNHSWNSQTWCTPAWNNQAWNNPFLNCGEETLQSCLQFQQNFLASDLEVALEAAGESLNVIQQATKYFSSPQAVESFLNYSMDMQPEDL

>gi_667308314_ref_XP_008583676.1__PREDICTED_homeobox_protein_NANOG_isoform_X1_Galeopterus_variegatus

MNVDPPCPQSQPCPEASDSRDSSPTPVICGPEEKYPPLQMSSAEMPHAETVSPLPSSTDVLIQDSPDSSTSPKVKLPASAEKSTVKKEEKVKKQKTRTVFSPTQLCVLNDRFQRQKYLSLQQMQELSSVLNLSYKQVKTWFQNQRMKCKRWQKTNWPKNSNSAAQKGSAPTEYPSLYSSYHQGCLVNTSGNFPLWSNQPWNNPAWSNQTWNSQSWSNHSWNSQTWCTQAWNNQAWNNPFHNCGEECLQPCTQFQQNYASDVESTMQTAGESHSVIQQTTKYFSNTQTMDLFLNYYMNMQPEDV

>gi_685570933_ref_XP_009178399.1__PREDICTED_homeobox_protein_NANOG_Papio_anubis

MSVDPACPQSLPCLAASDGKESSPMPVICGPEENYPSLQMSSAEMPHTETGKKDIYPFERLPLLFRAPCKTEEIVNFAVLEPVHLFCDCRTVFSSAQLCVLNDRFQRQKYLSLQQMQELSNILNLSYKQVKTWFQNQRMKSKRWQKNNWPKNSNGVTQKASAPTYPSLYSSCHQGCLVNPTGNLPTWSNQTWNNSSWSNQTQNIQSWSHHSWNAQTWCTQSWNNQAWNSPFSNCGEESLQSCLQFQPNSPASDLEAALEAAGEGLNVIQQTTRYLSTPQTMDLLLNYSTNMQPEDV

>gi_817319050_ref_XP_012330222.1__PREDICTED_homeobox_protein_NANOG,_partial_Aotus_nancymaae

MSMDPVCSQSLPCSEASDRGESSPTPVICGPEENYPTLQMCSAEMPHTETVSPLPSSMDLLIQDSPDSSTSPKGKQPIAAESSAAAKEDKVPVKRQKTRTVFSSAQLCVLNDRFQRQKYLSLQQMQELSSILNLSYKQVKTWFQNQRMKSKRWQKNNWPRNTSDATQKASAPTYPSLYSSYHQGCLVNTTGNFPVWNNQTWSNSTWSNQTRNIQSWSNHSWNTQTWCTQSWNTQAWNSPFYNCGEEPLQSCMQFQPNSPASDLEAALETAGE

>gi_296211302_ref_XP_002752348.1__PREDICTED_homeobox_protein_NANOG_isoform_X1_Callithrix_jacchus

MSMDPVCSQSLPCSEASDCGESSPTPVICEPEENYPSLQMCSAEMPHTETVSPLPSSMDLLTQDSPDSSTSPKGKRPTAAESSPAVKEDKVPVKKQKTRTVFSSTQLCVLNDRFQRQKYLSLQQMQELSNILNLSYKQVKTWFQNQRMKSKRWQKNNWPRNTSDVTQKASAPTYPSLYSSYHQGCLVNTAGNLPVWNNQTWSNSTWSNQTRNIQSWSNHSWNTQTWCTQSWNTQAWSSPFYNCGEEPLQSCMQFQPNSPASDLEAALETAGENHNVIQQTTRYLNTSQTMDLFLNYSTNMPPEDV

>gi_795569677_ref_XP_011909856.1__PREDICTED_LOW_QUALITY_PROTEIN_homeobox_protein_NANOG_Cercocebus_atys

MSVNPACPXNLPCSEASDCKDWSPPPAVCGSEENHPSLQMSSAEMPHTETVSPLPSSTDLLIQDSPDSSTSPKGKQPTAAENSATKKEXVKKQTRTLFSSAQLCVLNDRFQRQKYLSLQQMQELSNILNLSYKQVKTWFQNQRMKSKRWQKNNWPKNSNGVTQKASAPTSPSLYSSCHQGRLVNPTGNLPPWSNQTWNNSSWSNQTQNIQSWSNHSWNAQTWCTQSWNNQAWNSPFSNCGEESLQSCLQFQPNSPASDLEAALEAAGEGLNVIQQTTRYLSTPQTMDLFLXNMQPEVV

>gi_297691032_ref_XP_002822902.1__PREDICTED_homeobox_protein_NANOG_isoform_X1_Pongo_abelii

MSVDPACPQSLPCFEASDCKESSPMPMICGPEENHPSLQMSSAEMPHTETVSPLPSSMDLLIQDSPDSSTSPKGKQPTAAENSATKKEDKVPVKKQKTRTVFSSTQLCVLNDRFQRQKYLSLQQMQELSNILNLSYKQVKTWFQNQRMKSKRWQKNNWPKNSNGVTQKASAPTYPSLYSSYHQGCLVNPTGNLPMWSNQTWSNSSWSNQTQNIQSWSNHSWNTQTWCTQSWNNQAWNSPFYNCGEESLQSCMQFQPNSPASDLEAALEAAGEGLNVIQQTARYFSTPQTMDLFLNYSTNMQPEDM

>gi_332249388_ref_XP_003273845.1__PREDICTED_homeobox_protein_NANOG_isoform_X1_Nomascus_leucogenys

MSVDPACPQSLPCFEASDCKESSPMPVICGPEENYPSLQMSSAEMPHTETVSPLPSSMDLLIQDSPDSSTSPKGKQPTSAENSATKKEDKVPVKKQKTRTVFSSTQLCVLNDRFQRQKYLSLQQMQELSNILNLSYKQVKTWFQNQRMKSKRWQKNNWLKNSNGVTQKASAPTYPSLHSSYHQGCLVNATGNLPMWSNQTWSNSTWSNQTQNIQSWSNHSWNTQTWCTQSWNNQAWNSPFYNCGEESLQSCMQFQPNSPASDLEAALEAAGEGLHVIQQTARYFSTPQTMDLFLNYSTNMQPEDV

>gi_115392123_ref_NP_001065295.1__homeobox_protein_NANOG_Pan_troglodytes (Used in Figure 1C, mammal NANOG group)

MSVDPACPQSLPCFEASDCKESSPMPVICGPEENYPSLQMSSAEMPHTETVSPLPSSMDLLIQDSPDSSTSPKGKQPTSAENSVTKKEDKVPVKKQKTRTVFSSTQLCVLNDRFQRQKYLSLQQMQELSNILNLSYKQVKTWFQNQRMKSKRWQKNNWPKNSNGVTQKASAPTYPSLYSSYHQGCLVNPTGNLPMWSNQTWNNSTWSNQTQNIQSWSNHSWNTQTWCTQSWNNQAWNSPFYNCGEESLQSCMQFQPNSPASDLEAALEAAGEGLNVIQQTARYFSTPQTMDLFLNYSTNMQPEDM

>gi_397481205_ref_XP_003811843.1__PREDICTED_homeobox_protein_NANOG_Pan_paniscus

MSVDPACPQSLPCFEASDCKESSPMPVICGPEENYPSLQMSSAEMPHTETVSPLPSSMDLLIQDSPDSSTSPKGKQPTSAENSVTKKEDKVPVKKQKTRTVFSSTQLCVLNDRFQRQKYLSLQQMQELSNILNLSYKQVKTWFQNQRMKSKRWQKNNWPKNSNGVTQKASAPTYPSLYSSYHQGCLVNPTGNLPMWSNQTWNNSTWSNQTQNIQSWSNHSWNTQTWCTQSWNNQAWNSPFYNCGEESLQSCMQFQPNSPASDLEAALEAAGEGLNVIQQTARYFSTPQTMDLFLNYSTNMQPEDV

>gi_724942328_ref_XP_010386322.1__PREDICTED_homeobox_protein_NANOG_Rhinopithecus_roxellana

MSVDPACPQSLPCLEASDGKESSPMPVICGPEENYPSLQMSSAEMPHTETVSPLPSSMDLLIQDSPDSSTSPKGKQPAAENSATKKEDKVLVKKQKARTVFSSAQLCVLNDRFQRQKYLSLQQMQELSNILNLSYKQVGLFWKNDNGVTQKASAPTYPSLYSSCHQGCLVNPTGNLPTWSNQTWNNSSWSNQTQNIQSWSNHSWNAQTWCTQSWNNQAWNSPFYNCGEESLQSCLQFQPNSPASDLEAALEAAGEGLNVIQQTTRYLSTPQTMDLFLNYSTNMQPEDV

>gi_795107832_ref_XP_011808042.1__PREDICTED_homeobox_protein_NANOG_isoform_X1_Colobus_angolensis_palliatus

MSVDPARPQSLPCLEASDGKESSPTPVICGPEENYPSLPMSSAEMPHMETVSPLPSSMDLLIQDSPDSSTSPKGKQPTAAENSATKKEEKVLVRKQKARTVFSSAQLCVLNDRFQRQKYLSLQQMQELSNILNLSYKQVKTWFQNQRMKSKRWQKNNWPKKSNGVTQKASAPTYPSLYSSCHQGCLVNPTGNLPTWSNQTWNNSSWSNQTQNIQSWSNHSWNAQTWCTQSWNNQAWNSPFYNCGEESLQSCLQFQPNSPASDLEAALEAAGEGLTVIQQTTRYLSTPQTVDLFLNYSTNMQPEDV

>gi_635063215_ref_XP_007965674.1__PREDICTED_homeobox_protein_NANOG_isoform_X2_Chlorocebus_sabaeus

MSVDPACPQSLPCLEASHGKESSPMPVICGPGENYPSLQMSSAEMPHTETVSPLPSSMDLLIQDSPDSSTSPKGKQPTAAENSATKKEDKVPVKKQKARTVFSSAQLCVLNDRFQRQKYLSLQQMQELSNILNLSYKQVKTWFQNQRMKSKRWQKNNWPKNSNGVTQASAPTYPSLYSSCHQGCLVNPTGNLPTWSNQTWNNSSWSNQTQNIQSWSNHSWNAQTWCTQSWNNQAWNSPFYNCGEESLQSCLQFQPNSPASDLEAALEAAGEGLNIIQQTTRYLSTPQTMDLFLNYSTNMQPEDV

>gi_795229356_ref_XP_011851453.1__PREDICTED_homeobox_protein_NANOG_Mandrillus_leucophaeus

MSVDPACPQSLPCLEASDGKESSPMPVICGPEENYPSLQMSSAEMPHTETVSPLPSSMDLLIQDSPDSSTSPKGKQPTAAENSATKKEDKVPVKKQKARTVFSSAQLCVLNDRFQRQKYLSLQQMQELSNILNLSYKQVKTWFQNQRMKSKRWQKNNWPKNSNGVTQKASAPTYPSLYSSCHQGCLVNPTGNLPTWSNQTWNNSSWSNQTQNIQSWSNHSWNAQTWCTQSWNNQAWNSPFSNCGEESLQSCLQFQPNSPASDLEAALEAAGEGLNVIQQTTRYCSTPQTMDLFLNYSTNMQPEDV

>gi_567316091_ref_NP_001274577.1__homeobox_protein_NANOG_Macaca_fascicularis

MSVDPACPQSLPCLEASDSKESSPMPVICGPEENYPSLQMSSAEMPHTETVSPLPSSMDLLIQDSPDSSTSPKGKQPTAAENSATKKEDKVPVKKQKARTVFSSAQLCVLNDRFQRQKYLSLQQMQELSNILNLSYKQVKTWFQNQRMKSKRWQKNNWPKNSNGVTQKASAPTYPSLYSSCHQGCLVNPTGNLPMWSNQTWNNSSWSNQTQNIQSWSNHSWNAQTWCTQSWNNQAWNSPFSNCGEESLQSCLQFQPNSPASDLEAALEAAGEGLNVIQQTTRYLSTPQTVDLLLNYSTNMQPEDV

>gi_109095459_ref_XP_001112791.1__PREDICTED_homeobox_protein_NANOG_Macaca_mulatta

MSVDPACPQSLPCLEASDSKESSPMPVICGPEENYPSLQMSSAEMPHTETVSPLPSSMDLLIQDSPDSSTSPKGKQPTAAENSATKKEDKVPVKKQKARTVFSSAQLCVLNDRFQRQKYLSLQQMQELSNILNLSYKQVKTWFQNQRMKSKRWQKNNWPKNSNGVTQKASAPTYPSLYSSCHQGCLVNPTGNLPMWSNQTWNNSSWSNQTQNIQSWSNHSWNAQTWCTQSWNNQAWNSPFSNCGEESLQSCLQFQPNSPASDLEAALEAAGEGLNVIQQTTRYLSTPQTVDLLLNYSTNMQPEDV

>gi_795496091_ref_XP_011709333.1__PREDICTED_homeobox_protein_NANOG_Macaca_nemestrina

MSVDPACPQSLPCLEASDSKESSPMPVICGPEENYPSLQMSSAEMPHTETVSPLPSSMDLLIQDSPDSSTSPKGKQPTAAENSATKKEDKVPVKKQKARTVFSSAQLCVLNDRFQRQKYLSLQQMQELSNILNLSYKQSWNNQAWNSPFSNCGEESLQSCLQFQPNSPASDLEAALEAAGEGLNVIQQTTRYLSTPQTVDLLLNYSTNMQPEDV

>gi_513016540_ref_XP_004869600.1__PREDICTED_homeobox_protein_NANOG_Heterocephalus_glaber

MSAAAACAPGLSDSEAADNGKPAPRPPDAHSCPPPPAPVPPADAAASSLPSSMDLLLQEPPDSSTSPCIALPGSQEEVSGKEEGKVPAKKQKTPGSLKSEIIHTAEPGVPERVAVKTWFQNQRMKCKRWQKSNWPKNSSSIPQKGPAPAEYPGACSNYAQGYLVNTSGNLPVWGNQTWNNAGWSSPSWGSPSWNAQSWCPQAWSAPLPGFGEEALQPCLPFPQNFPASDLEAALEAVGDSYKYLSTPQSLDLFLNYPTNPQPGDL

>gi_612051977_ref_XP_007503681.1__PREDICTED_homeobox_protein_NANOG_Monodelphis_domestica (Used in Figure 1C, mammal NANOG group)

MSSCSHSQQCQSCRPKGDASRPYYTWISASQPESLTSPSRYSNCHIQSPGKVQPNTAISPAPSSMDKCIQDTPDSATSPTSNSLSSQNKPKTHQGKDQSPIKKPKMRTVFSQAQLNVLNSRFVEQKYLSPQQIRNVAENLNLTYKQVKTWFQNQRMKSKRWQKDTMWTKNGNRNVQNGSALGEYISLYSPFHQDYMVSSSGTLPVWSNQTWNNQFQNSGEGSYQHQIFQHSYPASDLGATFGNNTGGAYSMKSQTSLSFNTPYPMEYLPSYSMNMQLTHSKSEEDYDYRQASDAQTQFLDPSVVPVFQS

>gi_1019018285_ref_XP_016082410.1_PREDICTED_homeobox_protein_NANOG_Ornithorhynchus_anatinus (Used in Figure 1C, mammal NANOG group)

MIIITMKQPSEPGSSSMSAQLALPSYQPCPPDVAHAGPYFPEGGWAPQPEAYPAAPPFLPFQMPGAEKGRPDPELSPASSCSEKVSQSTPDSATSPSAHPSSPQAWPGSQAEEDQTKGQMKKSKIRTAFTQTQLNTLNRRFQTQKYLSPQQIRDLAMSLNLTYKQVKTWFQNQRMKSKRDRKDNLWTGRRVSMVQNGSLPATYMSLYSALQHSYLVNPATGNLPPWAGQAWASEPQGFVDGVGQQQQHPAEASQPFAKAMVGNYPPQQAALTFSSPGAADCLPAFPTDLPLAHIKAEDSYSDPTFLAAHSQFPDTSGLHLYQP

>gi_148357118_ref_NP_001091862.1__nanog_homeobox_Danio_rerio (Used in Figure 1C)

MADWKMPVSYNFNPSYHAYAYGLMYPQVSEHGVPNLSWPDAAYTHSGGVTAGYFTAQTAQSPPWSPENGGASSTYSQYPGHSQNGRLFLSYNKTEPDQKAKDAEQTSSDTPSDSEAHTPDSWSSASSREGVPLTNLNLPSWRDRDYETDSGSPDSGERNLTSTAGEEPVNLNLGVDTQPPLPALTASPVRPPTLPRKTRAAFSEEQMNALVNRFNVQRYLTPAEMKTLAGATGLTYKQVKTWFQNRRMKLKRHQRDSSWMTERYVVNAVPNTPASQSQFQSEPPGANQDHYINPQVREPVFKRSPPKTPFYPSYPQPRSPTQATSRPPGTWPLPPAVTHYEFPNPISYMPARDGSNAVNKESSPSPLATSPTAGLWATKGITLL

>gi_238054048_ref_NP_001153902.1__Nanog_protein_Oryzias_latipes (Used in Figure 1C)

MAEWKTQVNYNPTFHAYTYGFVYQTGPEQNHVTGNDWSQNCEQNGYNGGPTQSHFPARSREESPPRSPEQQPESGHYYQDSGVVYIREAQTGRLVMAGQHRVGLDSGENCTRRTGSDSASDSEAHTSPDSWSSCSNYDRSVPQTDPVVWVKNEEQTGARSPDHSEDVSSSLMVESQSFAVQDTGDASSSTHAPFTTTKKQASSTPNAPKAKVRAAFSESQMSTLVQRFSVQRYLAPAEMKNLADVTGLTYKQVKTWFQNRRMKLRRHQKDTSWVSERYTINKDNTAADTVFSNVAPHVPPYQGDGMSHLRHHYNQHMMGAAFKNTPHNLAFYLAAMGNPPGTAGYPPWSSSPPQAAVPSRPQVPGWPLPPGRSQFGFCPIPYDPSDAASLNNFERNAIPDSKDGESAGGANAAILHNAVQ

>gi_852770487_ref_XP_012879028.1__PREDICTED_homeobox_protein_NANOG_Dipodomys_ordii

MSVDCVCAPNVPRPEAADGEDGSPGPGSCQAREEEPRPSPEPTLHADTASPPPASVALRTQESRGSVSPLPARPATPDSRPGSVPGGQGDQGKPPAKRQKIRTVFTQMQLYYLNERFQKQKYLSLQQMQELSTLLNLTYKQVKTWFQNRRMKCKRWQKSRGLLDGGQPAQVSGLHPGHAPGWAGAPAASGGLPPWSDQMCSSQSWSGPAPGAHPWDPQPWNSTGPAWSHAFHSHPPQQPAQPCLPSQPDLPGASLEASMEISGGSHPYLDAPLGSDCYVNYAAGVL

>gi_637264368_ref_XP_003216891.2__PREDICTED_homeobox_protein_NANOG_Anolis_carolinensis Used in Figure 1C, NANOG 1 group)

MGLKAPATLDVLSSFPPWARGGCDGPCGEWHPGSRGSLLEGPRPSSALPPAPGSPREAPSLRGAPPPEADSGIKGPQGPSPPSGMGFDDYYVYPSGQRDAAAHYPEGFPARESLGGGEEEEEKEPRSQPDDSPQSYSSGTHTFYTPDSATSPNGPPSPQSTSQKLNGENNKGKKVKTRAAFSQKQLQILHHRFQNQKYLSPQQIRELAAALDLTYKQIKTWFQNQRMKFKRTQKESLWLRKGMCPPQNGFLQMNPNYHQGYGVGESRNIHALAGLHENFTSNQFYANNQNYTSDHQIYGNPQNLYPIANSEDGSFFGKATGASFNQQAVAYNGQQAVGYISQQKINFFHGFPTNMEYATVKTEDGYPFPNMSTTEAGSFPSSSGFQIYQLPLQTQGAQSNC

>gi_564257398_ref_XP_006267653.1__PREDICTED_homeobox_protein_NANOG_Alligator_mississippiensis

MSTHLAVPAAPRYGGYYWDCPPEPAAQPSEQDAAAPAPAPFPGDKTPQHPDLSPASSSSGMFIQYAPDSATSPNAEPLSPHLNPQRSGAGGEGGVKKAKTRTAFSPEQLQILHQRFQSQKYLSPYQIRELGSALGLTYKQVKTWFQNQRMKYKRYQKESQWIEKGTCLPQNGFYQAGYLDVGSGYYQGCSVSANRNIQTVANVHQTYNSSPAYSSSQSLYTFMAIEDEGTFFGKAASPCNTQQAVGFLSPQKVNFYHGFPTNVDYASVELEESYSFQNTPGTPVSFPGSGVRQPYQPVWHPQGTQSNYNS

>gi_557319049_ref_XP_006032699.1__PREDICTED_homeobox_protein_NANOG_Alligator_sinensis

MPYSLSHLLSVGSAISVLRNSTDIYVVKCCPELWVGYGDNCYLFCKERKDWNSSQESCAAESSSPGDQRHPGNDLSPASSSSGMFIQYTPDSATSPNAEPLSPHLNPQRSGAGGEGGVKKAKTRTAFSPEQLQILHQRFQSQKYLSPYQIRELGSALGLTYKQVKTWFQNQRMKYKRYQKESQWIEKGTCLPQNGFYQAGYLDVGSGYYQGCSVSANRNIQTVANVHQAYNSSPAYSSSQSLYTFMAIEDEGTFFGKAASPCNTQQAVGFLSPQKVNFYHGFPTNVDYASVELEESYSFQNTPGTPVSFPGSGVRQPYQPVWHPQGTQSNYNS

>gi_558147891_ref_XP_006119990.1_PREDICTED_homeobox_protein_NANOG_Pelodiscus_sinensis Used in Figure 1C, NANOG 1 group)

MSAHLAMPAYQAYPAGVGTGIKYGDYYWNCPGEMDSAPHKEAADADVAVPEPEEKPLPNPELSPASSSSGTLLRYTPDSATSPNAAPPSPHPAIRMGGGGSGGGVKKAKTRTAFSQEQLQTLHQRFQSQKYLSPQQIRELGSALGLTYKQVKTWFQNQRMKFKRCQKETQWMEKGTCLSQSGFHQAGYLDMNPSYHQGCPVSASRNIQTVTNVHQSYSSSNTYGSGQSLYPFMAIEEEGFFGKPGGACSAQQTMGFFSQQKVNLYHGYPANMDYASRETEDGYHFQNASVNAMSFPGSAGRQQYQPAWYPQGTQSNFNS

>ENSFALT00000007600_peptide:ENSFALP00000007568_pep:KNOWN_protein_coding_NANOG_Ficedula_albicollis Used in Figure 1C, NANOG 1 group)

MSAHLATPPYAPYPGAARYGDCYWLSAGSTDSAPAEEAAGLDALPLPAAPPPPPPLDSPASSSSGTLTQYHTPDSATSPTAAGSPSPHSSAQKVKAQGKGGVKSKSRTAFSQEQLRALHQRFQNQKYLSPQQIRELAAALELTYKQVKTWFQNQRMKFKRCQKESQWVDKGMYLPQNAAHQAAYLDMAPAFHQVFPVGGTTRSFQAVHNVHQAYSSGQSYGNGQNLYSFVSVEDVESVGLFGKGGTSCNTQQTVGLLSQQMNFYRSCFDNVDYVSVEVEDTFNFQNTSDNVTPYSSSPIENQCQLPWHPMGNQSGYESQV

>gi_449485131_ref_XP_002190766.2_PREDICTED_homeobox_protein_NANOG_Taeniopygia_guttata Used in Figure 1C, NANOG 1 group)

MLGAGGPGPAMSSHLGTPPYVLYPGAAKYGDYYWFSAGSMDSAPTEEAPAPDALPLPAAKTPSPSVDSPASSSSGTLTQYHTPDSATSPTAAGSPSPYSSLQKVKAQGKGVVKTGKSRTAFSQEQLKALHQRFQSQKYLSPQQIRELAAALELTYKQVKTWFQNQRMKFKRCQKESQWMDKGMYLPQNGVDQAAYLDMAPAFHQVFPAGSGRNFQAVSNVHQAYSSGQTYGNGQNLYSFSSVEDEGLFGKGGTSCNTQQTMGLLSQQMNFYHSYFDNIDYVSVEVDDTFNFQSTSDTVTPFSSSPIQNQCQLPWHPMGTQSGYESQV

>gi_930241053_ref_XP_014165108.1__PREDICTED_homeobox_protein_NANOG_Geospiza_fortis

MNSLRQSQELVMVSYRKKHKRIDSPASSSSGTLTQYHTPDSATSPTAAGSPSPHSSLQKVKAQGKGVVKTGKSRTAFSQEQLKALHQRFQSQKYLSPQQIRELAAALQLTYKQVKTWFQNQRMKFKRCQKESQWMDKGMYLPQNGVHQAAYLDIAPTFHQVFPAGSSRNFQAVSNVHQAYSSGQTYGNGQNLYSFPSVEDEGLFGKGGTSCNTQQTMGLLSQQMNFYHSCFDNIDYVSVEVEDTFNFQSSSDTVTPFSSSPIQNQCQLPWHPMGTQSGYESQV

>gi_1003728051_ref_XP_015727430.1__PREDICTED_homeobox_protein_NANOG_Coturnix_japonica

MSAHLAMPSYGSVRCGHYYWPSPSSMDSASTEEAPATDHSTAEQKTPCHPDASPASSSSGTLIQYTPDSATSPTGDHPSQHPTYQKVKDKGDSGARKGKSRTAFSQEQLQTLHQRFQSQKYLSPHQIRELAAVLGLTYKQVKTWFQNQRMKFKRCQKESQWVDKGIYLPQSGFHQAAYLDMTPTFYQGFPVGASRNLQAVTTAHQAYSSGQTYGNGPGLYQIVAMEDDEFFGKGGTSCNTQQAMGLLSQQMNFYHGYSTSVDYDSLQAEDTYSFQSASDSITQFSSSPVRHQYQGPWHTLGTQSGYET

>gi_225784819_ref_NP_001139614.1_homeobox_protein_NANOG_Gallus_gallus (Used in Figure 1C, NANOG 1 group)

MSAHLAMPSYGSVRCGHYYWPSPGSMDSASAAEAPAADLSLTTEQKTPCHPDASPASSSSGTLIQYTPDSATSPTADHPSHRPTFQKVKDKGESGTRKAKSRTAFSQEQLQTLHQRFQSQKYLSPHQIRELAAALGLTYKQVKTWFQNQRMKFKRCQKESQWVDKGIYLPQNGFHQAAYLDMTPTFHQGFPVVANRNLQAVTSAHQAYSSGQTYGNGQGLYPFMAVEDEGFFGKGGTSCNTQQAMGLLSQQMNFYHGYSTNVDYDSLQAEDTYSFQSTSDSITQFSSSPVRHQYQAPWHTLGTQNGYET

>gi_1121864621_ref_XP_003202657.2__PREDICTED_homeobox_protein_NANOG_Meleagris_gallopavo (Used in Figure 1C, NANOG 1 group)

MSAHLAMPSYGAVRCGHYCWPSPGSMDSASAEEAPAADLSLTAAQKTPSHPDASPASSSSGTLIQYTPDSATSPTADHPSHHPTFQKIKDKGESGARKAKSRTAFSQEQLQTLHQRFQSQKYLSPHQIRELAAALGLTYKQVKTWFQNQRMKFKRCQKESQWVDKGIYLPQNGFHQAAYLDMTPTFHQGFPVGANRNLQAVTSAHQAYTSGQTYGNGQGLYPFMAVEDEGFFGKGGTSCNTQQAMGLLSQQMNFYHGYSTNVDYDSLQAEDAYSFQSTSDSITQFSSSPIRHQYQAPWHTLGTQSGYET

>gi_874453809_ref_XP_012948354.1__PREDICTED_homeobox_protein_NANOG_Anas_platyrhynchos (Used in Figure 1C, NANOG 2 group)

MDRPADGCSPHYIWKYHSLYYFFPLSFQQRTPSHPDASPASSSSGTLVQYTPDSATSPTAERPSPHPSFQVKEKDEGAAKKGKSRTAFSEEQLQTLHQRFQAQKYLSPHQIRELAASLGLTYKQVKTWFQNQRMKFKRCQKESQWVEKGMYLSQNGFHQASYLDMTPTFHQGFPAGANRNLQAVTNMHQAYSSGQTYGNGQCLYPFMAVEDEGFFGKGGTSCNSQQAVGLLTQQMNFYHGYPTSVDYDGLQSEDTYSFQSTSDGIMQFSSSPVRHQYQAPWHTLETQSGYES

>gi_699644435_ref_XP_009894969.1__PREDICTED_homeobox_protein_NANOG_Picoides_pubescens

MSTHLAMPPYMPYPGAFSCGDYYWLSPGSTESSEPAEEAPPADTVPCAAPEKTPSQPDVSPASSSSGMLTQYTPDSATSPTAERPSPHPSSQKVKEEGEGVVKKTKSRTAFSQEQLQILHQRFQSQKYLSPQQIRELAAVLGLTYKQVKTWFQNQRMKFKRCQKESQWVEKGVYLPQQGFHQAAYLDITSNYHQGFPVGANRNLQGVTNVHQAYSSGQTYGNGQNLYSFMAVEDEGLFGGKGGTSCNTQQAMGLLTQQMNYYHGYSADMDYVSLEPEDTYGFQNTSDNIAPFSGSPVQHQYQAPWHPLGTQSGYES

>gi_541990976_ref_XP_005446141.1__PREDICTED_homeobox_protein_NANOG_Falco_cherrug

MLQPDVSPTSSSSGTLTHYTPDSATSPTAERPSPHAPLQKAKEEGEGMMKKAKSRTAFSQGQLQILHQWFQTQKYLSPQQIRELAALLGLTYKQVKTWFQNQRMKFKRCQKETQWMEKGMYLPQNGFHQAAYLDITPTFHQGFPVSASRNLQAVTNMHQAFSSGQTYGNGQSLYSFMAVEDEGLFGKGGTSCNTQQAMGLLSQQMNFYHGYPADVDYVSLESEDTYSFPSTSDSGTPFSGSPVWHQYQAPWHPLGTQGGYES

>gi_529429355_ref_XP_005234494.1__PREDICTED_homeobox_protein_NANOG_Falco_peregrinus

MSAHLAVQPYLPYPDAVMYGDYFWFSPGSMDCVPAEEAAAADPLPFLAAEKTPSHPDVSPTSSSSGTLTHYTPDSATSPTAERPSPHAPLQKAKEEGEGMMKKAKSRTAFSQGQLQILHQWFQTQKYLSPQQIRELAALLGLTYKQVKTWFQNQRMKFKRCQKETQWMEKGMYLPQNGFHQAAYLDITPTFHQGFPVSASRNLQAVTNMHQAFSSGQTYGNGQSLYSFMAVEDEGLFGKGGTSCNTQQAMGLLSQQMNFYHGYPADVDYVSLESEDTYSFPSTSDSGTPFSGSPVWHQYQAPWHPLGTQGGYES

>gi_543737791_ref_XP_005509821.1__PREDICTED_homeobox_protein_NANOG_Columba_livia

MNAHLAMPPYLPYPGAVRYGDYYWLSPGSMDSMPAEEAPPADALPFPAVEKTPSHPDISPASSSSGTLIQYTPDSATSPTTDRPSPHPSSQKVKEEGEGVVKKAKSRTAFSQEQLQILHQRFQSQKYLSPQQIRELAAALGLTYKQVKTWFQNQRMKYKRCQRESQWVEKGVYLPQNGFHQAAYLDIAPTFHQGFPVSTSRNLQAVTNMQQAYSAGQTYGNGQSLYSFMAVEDEGLFGKGGTSCNTQQAMGLLSQQMNFYHGYTADMDYVNLQSEDTYGFQSTSDSVTQFSSSPVRHQYQAPWHPLGTQSGYES

>gi_701426577_ref_XP_010002458.1__PREDICTED_homeobox_protein_NANOG_Chaetura_pelagica

MGKEFQDNMGDLTVAFQYLEGPTRKMRRDCLQGHVVTQQHHTETLVQFPYMDTETEEIKTTDDALLLLGELVGLTEQQSETAATRVCGQSPYTGNVEQRNEAARSLRPLQGGHGNKQAMCFMWSPLLASYTFLQAGDVSPASSSSGTLIQYTPDSATSPTAERPSPHPSSQKAKEEGEGVVKKAKSRTAFSQEQLQVLHQRFQNQKYLSPQQIREMAAALGLTYKQVKTWFQNQRMKFKRCQKESQWVEKGVYLPQNGFHQAAYLDITPTFHQGFPVSASRNLQAVTNMHQAYSGGQAYGNGQSLYSFVAVEDEGLFGKGGTSCNTQQAMGLLSQQMNFYHGYPADMDYVSLESEDTYGFQSTSSSMTLFSSSPVRHQYQAPWHPLGTQSGYES

>gi_696985274_ref_XP_009560768.1__PREDICTED_homeobox_protein_NANOG_Cuculus_canorus

MFNSAAGDSPASSSSGTLIQYTPDSATSPTAERPSPHPSFQKVKQEGEGVVKKAKSRTAFSQEQLQMLHQRFQSQKYLSPQQIRELAAALGLTYKQVKTWFQNQRMKFKRCQKESQWVEKGVYLPQNGFHQAAYLDIPPTFHQGFPVGASRNLQHVMTNMHQAYSSSQTYGNGQSLYSFMAVEDEGFFGKGGTSCNTQQAMGLLSQQMNFYHSCPANMDYVSLESEDTYGFQSTSDSIAPFTSSPVRHQYQAPWHSLGTQSGYES

>gi_558147891_ref_XP_006119990.1_PREDICTED_homeobox_protein_NANOG_Pelodiscus_sinensis Used in Figure 1C, NANOG 2 group)

MSAHLAMPLYQPYPSGTGMSYGEFYWNSAGGTERAASWPGRGAVAAGDAQFPEEERQASSSSGNFSHFTPDSATSPQTESSPPQPASKLQKDGKEGEKGVKKAKSRTAFSKEQLKTLHQRFQSQKYLSTQEMLELATALGLTYKQVKTWFQNRRMKLKTYEKHNLWSERAQYLMQTGFQPSEYLEVHPKFHQNYPISLAGTIQDVVNPHQNYSSGQNPYAFIASEEGGVFGKGGATCSVQQTVGFIAQRKVDFYHGFSGTMEYTGAKTGDGYSFHASATVAPFPGTAGHHLYLPEAELMQMAQSNCH

>gi_733871376_ref_XP_010715694.1_LOW_QUALITY_PROTEIN_homeobox_protein_NANOG_Meleagris_gallopavo Used in Figure 1C, NANOG 2 group)

MGGYTYNSGYTYGLGDEMLKSRPLERNLEILVNDVSSASSSSGNFSQFISDLAISQHSASSSRQPTTKLQNGREYDMGTVKKAKSRTAFSXEQQQTLHQHLQSQKYFSPHQIQELAAALGLTYHQVKTCFQNQRRKLKRCQKNTLWTAWIQCVMQNSFHPSSYLDVYPKFHQVYPISAASNIQIMPTPCQHYRAGQKAYIILTSEDGGVFYKGGSICSIQQTVGFIAQHKVGFYYSYPGSVEYLCTKTSGGYNFHQSAPMGASFPATAGHHLYHS

>gi_449485131_ref_XP_002190766.2_PREDICTED_homeobox_protein_Taeniopygia_guttata Used in Figure 1C, NANOG 2 group)

MCAQLALPPCPAYPGGAARGCLELRWGAAGQAAPAPAAGLSPEAAGSRHTAEVSPASSSSGNFSQFTPDSATSPHSSSTSPQPTAKSQKGREYGVEGIRKTKSRTAFSKEQLLTLHQRFQSQKYLSPQQIRELAVALGLTYKQVKTWFQNRRMKLKRCQKQSLWSERAQCLTQSGFQSGTYLDVHPKFHQGYPIATAGNIQTVPAPCQHYGAGQNAYTIVTSEDGGVFGKGSVQQTVGFIAQHKVDFYHSYPGSVEYPGSKTGDGCNFHHSATMGAPFPTAASHHLYHS

>ENSFALT00000007595_peptide:ENSFALP00000007563_pep:KNOWN_protein_coding_Ficedula_albicollis Used in Figure 1C, NANOG 2 group)

MYQACPGGAALGYLELHWGEPRPAPTAAGPAAIAAAGPCPEAAARRHTAEVSPASSSSGNFSQFTPDSATSPHSSSSSPQPTAKCQKGRECGVEGMRKTKSRTAFSKEQLQTLHQRFQSQKYLSPQQIRELAVALGLTYKQVKTWFQNRRMKLKRCQKHSLWSERAQCLTQSGFQTSTYLDVHPKFHQGYPITAAGNIQTMPAPCQHYGAGQNAYTIVTSEDGGVFGKGGGTCSVQQTVGFIAQHKVDFYHSYPGSMEYPGSKTGDGCNFHHSATMGAPFPTTASHHLYHS

>gi_698383276_ref_XP_009810570.1__PREDICTED_LOW_QUALITY_PROTEIN_homeobox_protein_NANOG,_partial_Gavia_stellata

MCAHLALPPYQAYPSGTGMGYLEFYWNSAGEAXGXXXXTNASQSPEAGGKRQTAEVSPASSSSGNFSQFTPDSATSPHSASSSPQPTAKSQKGREDGMEGVRKAKSRTAFSKEQLQTLHQRFQSQKYLSPQQIRELAAALGLTYKQVKTWFQNRRMKLKRCQKHSLWAERAQCLTQ

>Unanotated_Chr_1:75359239_75360897_homeobox_protein_NANOG_Gallus_gallus (Used in Figure 1C, NANOG 2 group)

MCAQLALPPYQAYPGGSGMGYLEFCWSSAGDTGHVPVSAGPAAAASASPSPEAGQHRQTTEISPASSGSGNFSQFMPDSATSPHSASSSPQPTAKPQKGREDVMGTVKKGKSRTAFSEEQLQTLHRRFQAQKYLSPHQIRELAVALGLTYQQVKTWFQNRRMKLKRCQKHILWTARAQCLTQNSFQPSTYLDMHPKFHQGYPINAASNIQTVSSPRQHYGAGQNAYATVTSEDGGVFGKGGGTCSVQQTVGFIAQHKVDFYHSCPGSVEYPGTKTGDGCNFHHSAPMGASFPTTAGYHLYHS

>gi_878283600_ref_NP_001297341.1_homeoboc_protein_NANOG_Anas_platyrhynchos (Used in Figure 1C, NANOG 1 group)

MCAQLALPPYQAYPGGSGMGYLEFCWSSAGDTGHVPVSAGPAAAASASPSPEAGQHRQTTEISPASSSSGNFSQFMPDSATSPHSASSSPQPTAKPQKGREDVMGTVKKGKSRTAFSEEQLQTLHQRFQAQKYLSPHQIRELAVALGLTYQQVKTWFQNRRMKLKRCQKHILWTARAQCLTQQNSFQPSTYLDMHPKFHQGYPINAASNIQTVSSPRQHYGAGQNAYAIVTSEDGGVFGKGGGTCSVQQTVGFIAQHKVDFYHSCPGSVEYPGTKTGDGCNFHHSAPMGASFPTTAGYHLYHS

>sarcopus_nanog___Unanotated_protein (Used in Figure 1C, mammal NANOG group)

GRGVWRPVPLPLPSPESLVARAPASLPGKWDADRAPRLSLLSPPPAFAPAPQSLNALVQDPPGSLSGPTVQENKVDQSKGKKQKTRTTFTQEQLNLLRSDFALSRYITPQRGRQLAQLLGLSYKQVQVKTWFQNQRLKAKRPQAALASSPHDPRLPQVKRSPLALGFPALSGSRAPMGGPVLSGNPSPPSFSWDRLLDHKASASPRHFPWTL

>gi_395541126_ref_XP_003772498.1_PREDICTED_NANOG_homeobox_protein_Sarcophilus_harrisii (Used in Figure 1C, mammal NANOG group)

MTSCSLSQQCQSSQPEGDASCPYNTWISASQPEPFTASSPYSNFQIQSPEKLQPSTAISPATPTSVDMFVQDIPDSATSPTSNNLSSQNKPKIHQGKEDQNLVKKSKMRTVFSQAQLNVLNSRFLEQKYLSPQQIRNVAENLNLTYKQVKTWFQNQRMKSKRWQKDSMWSKNSNNMVQNGSTVGEYISFYSPFHQDYMVNPSGNLPMWSNQTWNNQFQNGGEGSYQHQVFQHSYPASDLGAPFGNTISEVYPMKPQMTMSFSAPHTMEYLPSYSMNMQLTHKSEEDYDYQQVSDVVQTQFLDPSGVPIFQS

>gi_620949491_ref_XP_007661306.1_PREDICTED_NANOG_neighbor_homeobox_like_Ornithorhynchus_anatinus (Used in Figure 1C, mammal NANOGNB group)

MAPLDQTHGSGSSSGLISIARNNDEGNEMEKKEEEGKERNVAKRKMDRVVVHKTSQNRPVSKSIMETLWMTFKMKHRVRTPEAVRLAGDLAISVPQVTAWFQSTRRKYREMATVQKKATKSVQVS

>gi_620967693_ref_XP_007657462.1_PREDICTED_NANOG_neighbor_homeobox_like_partial_Ornithorhynchus_anatinus (Used in Figure 1C, mammal NANOGNB group)

LQGHAATVVHSLHEAPIWKAKSLQSARFKERIYTRLLLPESIVSRGASFRSFIQAFPKVSLVEMEMMSSVTFPLFEGDKEIDQLWNENYTQTCRHPPRKNASQSAAPFCPLELLMAPPDQAHGSVPSSGLISIARNKDEGDGVEETEAEGKESNAAETNRMVVHKTSRNHPVSKSIMETLWATFKVKHRVRTPEAVRLAGDLAISVLQVKAWFRSTRRKHRKMAKGQRRVTKSKMQSVLRMKSSVICYMCCA

>gi_830179254_ref_XP_012587790.1__PREDICTED_NANOG_neighbor_homeobox_Condylura_cristata

MPGEEAPEPSTRHRSKERSGTKRSEQKEKNGQVEQYPQTRLISKPLMNTLWTKFKLRKYLTIGDRLSLAFEFSLTDKQISQWFCEKREKYKEEIYKQKYKKKHKKLVKDALLTDKIKIKEQLAECENVFNEVQEEQVATSLKDEDSDCETKRKTKRCLRAPTPIRDTEGSTSLSEEESELEKGIEELEGRLKRVNPLAPGSTSPMTTAPPPYNPGWDPGTEKPIWVPERCTRPVEESSRQNEAPTVADPVADISNPSSECQVTPHALDPADDIPSAQVR

>gi_1008791739_ref_XP_015841837.1__PREDICTED_NANOG_neighbor_homeobox_Peromyscus_maniculatus_bairdii

MKQSLDMENVTTSERNLLKKKLIAHHGRQDPEHSSWSSSKGGRKKKTEERKKPEAKLKKRVKQKKQVPRPEKSVSKPLMDTLWARFKSRLLPTAKDCYLLSFQFSLTDKQIFQWFWEKRKKYKEERKTGPAAEQKKQDPGDADPLC

>gi_852770483_ref_XP_012879027.1__PREDICTED_NANOG_neighbor_homeobox_Dipodomys_ordii

MVMMMVMVMVVLVLTVALAVAGLVMKMAEVSSRRRATSSGSANPQPSDTMELEGKGRERQKCRRCGKWKRQKEPKKTQKEEDLTEIARKEEQEACSTKPRVSKSLLETLWAKFKLTRTPSVRDYLWLSFEFNMTGAQIRQWFYEKRKQYSKEMYKHRRKKRQER

>gi_731480294_ref_XP_010589223.1__PREDICTED_LOW_QUALITY_PROTEIN_NANOG_neighbor_homeobox_Loxodonta_africana (Used in Figure 1C, mammal NANOGNB group)

MGKRILLHGKKTAMPYNQNPEQSTRNHSEDEKKGKEKWENEEGEEERKEEKRKWEETLYPKKRLLSKSLMDNLWANFKXNKCPRERDYLSLSFQFNMTDKQIKEWFSEKRKKYKKDMFKGNITKDTRSDRNSEKHGWMPKETKPQRKGEFNHRRARQSGRVRQGPVSKPLLDVLWIHFKHNSNVNVRQSQVLAKFLDLSPYQDSQL

>gi_640805862_ref_XP_008059375.1__PREDICTED_LOW_QUALITY_PROTEIN_NANOG_neighbor_homeobox_Tarsius_syrichta

MSQVVRNNGYITSDVSYWILDHLVQELLHVSFTSHDYLSDLHIKAKQSNWNYREDEEKGEQKWRKEGRGGSRNEXEKERKEEKGKSKEPYPRRXLVKSFMDTLWAHFKLNKSPIIQDSLSLSFELGMTHHQISQXFYKKRKKYKKEMSKWKHNR

>gi_931580426_ref_XP_014202566.1__PREDICTED_LOW_QUALITY_PROTEIN_NANOG_neighbor_homeobox_Pan_paniscus

MHRVRWLTPVIPALWEAEAGRSRGQEIETILANKPPWTALDCLSLFNIVMLDEDEQNGKQKWREEGGEAGRKREREKEXKNEKELQDEQENKRKRENEKQKQYPEKRLVSKSLMDTLWAKFKLNRCPTIQESLSLSFEFDMTHKQISQWFCKTRKKYNKEMSKRKHKKKHTGWRSLCCQGWSRTPALK

>gi_821033309_ref_XP_012357801.1__PREDICTED_LOW_QUALITY_PROTEIN_NANOG_neighbor_homeobox_Nomascus_leucogenys

MPVIPALWEAEAGRSRGQEIETILANMKQPAMPWDQNPEQSTGNYSEDEQNRKQKWREGGGEAGGKTEREKEDKNEKELQDEQEKKRKRENEKQKQYPEKRLVSKSLMDTLWAKFKLNRCPTIQESLSLSFEFDMTHKQISQWFCKRRKKYNKEMSKRKHKRKHMRWRSLCCQGWSRTPALK

>gi_686739556_ref_XP_009245676.1__PREDICTED_NANOG_neighbor_homeobox_Pongo_abelii

MPVIPAPWEAEAGGSRGQEIETILANKRQPAMPWDQNPEQSTGNYSEDEQNGKQKWREGGGEAGRKREREKEEKNEKELQDEQENKRKRENEKQYPEKRLVSKSLMDTLWAKFKLNRCPTIQESLSLSFEFDMTHKQISQWFCKTRKKYNKEMSKRKHKEKHMRWRSLCCQGWSRTPALK

>gi_1034100027_ref_XP_001164884.3__PREDICTED_NANOG_neighbor_homeobox_Pan_troglodytes (Used in Figure 1C, mammal NANOGNB group)

MHRARWLTPVIPALWEVEAGRSRGQEIETILANKKQPAMPWDQNPEQSTGNYSEDEQNGKQKWREEGGEAGRKREREKEEKNEKELQDEQENKRKRENEKQKQYPEKRLVSKSLMDTLWAKFKLNRCPTIQESLSLSFEFDMTHKQISQWFCKTRKKYNKEMSKRKHKKKHTRWRSLCCQGWSRTPALK

>gi_74759191_sp_Q7Z5D8.1_NANGN_HUMAN_RecName_Full=NANOG_neighbor_homeobox;_AltName_Full=Homeobox_protein_C14 (Used in Figure 1C, mammal NANOGNB group)

MHRARWLTPVIPALWEAEAGRSRGQEIETILANKKQSAMPWDQDPEQSTGNYSEDEQNGKQKWREEGEAGRKREREKEEKNEKELQDEQENKRKRENEKQKQYPEKRLVSKSLMHTLWAKFKLNRCPTIQESLSLSFEFDMTHKQISQWFCKTRKKYNKEMSKRKHKKKHMRWRSLCCQGWSRTPALK

>gi_426371498_ref_XP_004052683.1__PREDICTED_NANOG_neighbor_homeobox_Gorilla_gorilla_gorilla

MKQSAMPWDQDPEQSTGNYSEDEQNGKQKWREEGGEAGRKREREKEEKNEKELQDEQENKRKRENEKQKQYPEKRLVSKSLMHTLWAKFKLNRCPTIQESLSLSFEFDMTHKQISQWFCKTRKKYNKEMSKRKHKKKHMRWRSLCCQGWSRTPALK

>gi_725592896_ref_XP_010347133.1__PREDICTED_NANOG_neighbor_homeobox_Saimiri_boliviensis_boliviensis

MLTPPASYQKAGTHYDSHQKPQETSRIFFGRARWLTPVIPALWEAEVGGSRGQEIETILVNRKQPAMPWDQNPEQSNGNYSEDEQKEKQKSREGGGEAGGKIEQEKEEEMEKDVDNEQEKKRTMENEKQKQYPKTRLVSKSLMDTLWAKFKLNRFPTIQESLWLSFEFGMTYKQISQWFYKKRKKYNKEMSKKKRKKKHEKGSLRCRGWS

>gi_966970433_ref_XP_015006323.1__PREDICTED_LOW_QUALITY_PROTEIN_NANOG_neighbor_homeobox,_partial_Macaca_mulatta

WWLMPIIPAPWEAKAGRSXGQEIKTILANKKRPAMSXDQNPEQSTENYSEDEQNGKQKWREGGREAGRKREREKEEENEKELEDEPENKRKRENKKHKQYPEKRLVSKSLMDTLWAKFKLSRCPTIQESLSLSFEFDMTHKQISQWFCKKRKKYNKEMSKRKHKKNIRDFIKYFDIEMAFIIHMRNHLTPT

>gi_544469178_ref_XP_005596075.1__PREDICTED_LOW_QUALITY_PROTEIN_NANOG_neighbor_homeobox_Macaca_fascicularis

MTRVLQKLQLKRTNLDKNCRAQWLTPVIPALWEGEAGRSQGQEIKTILANKKRPAMSXDQNPEQSTGNYSEDEQNGKQKWREGGREAGRKREREKEEENEKELEDEPENKRKRENKKHKQYPEKRLVSKSLMDTLWAKFKLSRCPTIQESLSLSFEFDMTHKQISQWFCKKRKKYNKEMSKRKHKKNIRGKKVFLVK

>gi_724942376_ref_XP_010386348.1__PREDICTED_NANOG_neighbor_homeobox,_partial_Rhinopithecus_roxellana

RWLKPVIPALWEAEAGGSQGQEIETILANTKQPAMSWDQNPEQSTGNYSEDEQNGKQKWREGGGEAGRKREREKEEENEKELEDEPENKRKRENEKQKQYPEKRLVSKSLMDTLWAKFKLNRCPTIQESLSLSFEFDMTHKQISQWFCKKRKKYNKEMSKRKHKKKTYKVRKCFL

>gi_795150726_ref_XP_011838477.1__PREDICTED_NANOG_neighbor_homeobox_Mandrillus_leucophaeus

MKDSVLPQQWNSPLSSLLLSQGVYLGRAWWLTPIIPGLWEAEAGRSQGQEIETILANTKRPAMSYDQNPEQSTGNYSEDEQNGKQKWREGGREAGRKREREKEEENEKELEDEPENKRKRENEKHKQYPEKRLVSKSLMDTLWAKFKLNRCPTIQESLSLSFEFDMTHKQISQWFCKKRKKYNKEMSKRKHKKKHTR

>gi_685570929_ref_XP_003905976.2__PREDICTED_NANOG_neighbor_homeobox,_partial_Papio_anubis

RWLKPVIPALWEAEAGGSRGQEIETILANMKRPAMSYDQNPEQSTGNYSEDEQNGKQKWREGGREAGRKREREKEEENEKELEDEPENKRKRENEKHKQYPEKRLVSKSLMDTLWAKFKLNRCPTIQESLSLSFEFDMTHKQISQWFCKKRKKYNKEMSKRKHKKKHTR

>gi_826346799_ref_XP_012518544.1__PREDICTED_NANOG_neighbor_homeobox,_partial_Propithecus_coquereli

MDLNQQGLEDLVIQKQPAMAWDPGPEQSSRNYGKDEGKRKQEWREETGGGEKEEEVEKELEEEQKKEKKKE

>gi_831236958_ref_XP_012664751.1__PREDICTED_NANOG_neighbor_homeobox,_partial_Otolemur_garnettii

RWLTPVIPAFWEAETGRQLETLVSRKKKVAMPWDQNPEQSHRNYSEHERKRKQKWREEERGGQGTGKEKEEEIEKELEEEQKEEKKKEKENEEQHPRKRLVSKPLMDTLWGKFKLNKYLTIQDSFSLSFEFSMTHKQINQWFCKKRKKYNKAMSEQKYNKSLRGKQNRTLRAYCKHQGRTPLQTLNATCLQL

>gi_829830075_ref_XP_012633078.1__PREDICTED_NANOG_neighbor_homeobox_isoform_X1_Microcebus_murinus

MLWDPSPEQSSKNCKEEEGTKKQKCREEETAGGGDKEEEVEKELEEEQKKEKENGEGCPRKRLVGKSLMDTLWGKFKLNKYLTIQDTLSLSFEFSMTNRQINQWFCRKRKTYNKEMSKRKYNKRRKGESSTKYGVMTTHTRGGLPLHRI

>gi_674105033_ref_XP_008823724.1__PREDICTED_LOW_QUALITY_PROTEIN_NANOG_neighbor_homeobox_Nannospalax_galili

MDKLIKKFANARITPVTGREREDEIEEKRMDTANTVKNKVGMKSTSMKSELTKPETVRLILERSNKGEAPALLVKTIQDLDKDRLIGKRLALNVESDTGKSAQHAEVIAAKLAVQQSFDEKQKVCYIFTDSWCVANGIAIWSEIHKLRWVWWRTPLIPAXWEAEAGNLVVIPSQPDTHCAHNPEQSKRNPGEDEAKQELKWREGGVKEKEKKREEKKQDATTCPRKMSVSRSLMDTLWAKFKSRQCPTVQDCLSLSFEFSITDKQIRQWFCKKRKKYKKEMSKQKQGRKRKKAKCGAAHL

>gi_741878262_ref_XP_010826221.1__PREDICTED_NANOG_neighbor_homeobox_Bos_taurus (Used in Figure 1C, mammal NANOGNB group)

MDRAPNTPSQSEYLISESQGQHLVQKTQRKTITDLCQQRLKMGDNFTKSESWNSGAQKQPVMPCDQNHLEVERKTKKRKRGGGEKEEVIEENLEKEEDEKEKKNKKRYQKKKFVSRPLMDTLWAMFKFKKNPTFQDISSLALEFSMTATQINRWFCKKRKIYKKEIHWRAYKNRRDPK

>gi_594093047_ref_XP_006069852.1__PREDICTED_NANOG_neighbor_homeobox_Bubalus_bubalis

MDRAPNTPNKRGLGIDHWIFAMLRSLIGDFDKRDVCGVGYHERYQIFICVSSSTYARGGEKEEVIEENLEKEKDEKEKKNKKRYQKKKFVSRPLMDTLWAMFKFKKNPTFQDISSLALEFSMTETQINRWFCKKGKIYKKEIHWRAYKNRRDVKYIWCLSFLTVVWKKLI

>gi_532113471_ref_XP_005341413.1__PREDICTED_NANOG_neighbor_homeobox_Ictidomys_tridecemlineatus

MNDYQQGLEVDDNLIKPKSSNLVTHKEPAMPQSQSPEHSPECPSEDEGKEERNWREEAEEEEEEEEEEEEKLKDTVKNAEEHEELCPRKRRVSKSLMDTLWAKFKLSAWPTVQDRLSLSFEFSMTDKQIQQWFSKKRKKYMKELSEQKRSKRLKNKKASTHKTSKLQ

>gi_987946191_ref_XP_015419561.1__PREDICTED_NANOG_neighbor_homeobox_Myotis_davidii

MDSPAKRNTPSTQEFSQTPGEGYAADPQAVSGLAQNLSNLTLTPSTESPSLPPKPSPQQQDIGNNLKASELGLNQGMLYTRRRGARTVASVQREMKEMRERIRLMMIRRADAIKYRTLYLGTPEHDCLEVLMEVYSSQSDLTDGPLQNPDRVLFTDGSSFLDAGKRQAGKAKHANIYTDSRYAFMEVYSSQSDLTDGPLQNPDRVLFTDGSSFLDEGKRQAGSRKHLSDGDLSPALRETDLAIQKQPVMPCDTNPEQSNRNHSKDERRKNTKWREGEGEMEEEMEKDGKKKLEEEQEEEKKEEKMECEEEYPEKRLVSKPLMDTLWATFKLNKCPTKGHSLSLAFEFNMTEKQVNQWFFKKRKKFKQEMYKQKYKRKLKRSGLLFLPLYVPIALGQGFLLKGMDMGKLFCQAQISPAGVAEAAVSPRTSDTARTR

>gi_989963297_ref_XP_015446944.1__PREDICTED_NANOG_neighbor_homeobox_Pteropus_alecto

MERGPDTSEVPDTALALQKQPFVPRDQNPEQSNRNHIKDERQEKKKERKGGGGKEENEKEVEEKLEEEQETEEEKELKMENDEEYPEKRLVSKPLLDTLWAMFKLNKCPTIGDSLSLAFTFNMTEKQINQWFFKKRKKFNKEMYKQKYKKKRKRYSAITYLIDYSGVLEKVSN

>gi_759136848_ref_XP_011364240.1__PREDICTED_NANOG_neighbor_homeobox_Pteropus_vampyrus

MDSPQKLNPTPTLESSQKPSEDSAGSQAVSEVLAKNLSNLTLNPSTKFPFLLPECSPQQQNREKDLQGLGLGLGQGILYRRRRGVRTLATARKERMQRMLQIIRYRTQSLLRKDPQERKIEAQLKSKPALLSDEQEGSIHSVDVLNKRTIWVQDRMAWGSQGFHHAAQNALALQKQPFVPRDQNPEQSNRNHIEDERQEKKKERKGGGGKEENEKEVEEKLEEEQETEEEKELKMENDEEYPEKRLVSKPLLDTLWAMFKLNKCPTIGDSLSLAFTFNMTEKQINQWFFKKRKKFNKEMYKQKYKKKRKRKKNNEEITNFCGYVQRIKRKATVESNGFSAFCNLSLDL

>gi_927132211_ref_XP_005655747.2__PREDICTED_NANOG_neighbor_homeobox_Sus_scrofa

MDLCPQGLEPGDSFTESASENLAAQKPPALPCDPIPERSQSNHPEAERMGKKRRREEEEGEKEEVIEENVAKKEKEENEELHDKKRLASKPLLDTLWATFKLIRFPTRGVISSLAFEFSVTETQINQWFRQKRKLYKREIYKRWKRC

>gi_671009744_ref_XP_008695737.1__PREDICTED_NANOG_neighbor_homeobox_Ursus_maritimus

MDSPKQLYPTWAPGSSQMSSEENSQEASGDSQTISEILTKNLGKLTLDSSTKLPSPLPEYPSQQQDREKKPQGLVECILSSSRRRTGVRTLLIARKERMAKMIRMIQYQRYLSPGSQLQKDLQQKETEEKEPTVLCDQNPERSKRNHSEDERRGNKKWNNVPRIVSKPLMDTLWATFKLNKCPTRGESQLLAFEFNMTAKQIKQWFRNRRKKYNKDMCKQKHKKRLKR

>gi_545219587_ref_XP_005611089.1__PREDICTED_NANOG_neighbor_homeobox_Equus_caballus (Used in Figure 1C, mammal NANOGNB group)

MDLYQQGLEAGDNFTKSKSWSTPSSQNNWIGLKCNINPLLEDGINGIDGINGMSFEETLSSALQFLAQDEQYMNHLQHLQDTKTCDKAFLNSSDATRSADLATQKQLPKPRDQNSDQSNRNHSEGERSGEKKQEEDQNERLVSKPLMDALWATFKMNRCPTIGDKLSLAFEFNMTEKQIDQWFCKKRKKYNKEMKKRKYKKRLKSCFILSYSFEAT

>gi_664739596_ref_XP_008527097.1__PREDICTED_NANOG_neighbor_homeobox_Equus_przewalskii

MDLYQQGLEAGDNFTKSKSWSTPSSQNNWIGLKCNINPLLEDGINGIDGIDGMSFEETLSSALQFLAQDEQYMNHLQHLQDTKTCDKAFLNSSDATRSADLATQKQLPKPRDQNSDQSNRNHSEGERSGEKKQEEDQNERLVSKPLMDALWATFKMNRCPTIGDKLSLAFEFNMTEKQIDQWFCKKRKKYNKEMKKQKYKKRLKSCFILSFYSFEAT

>gi_928173446_ref_XP_005637249.2__PREDICTED_NANOG_neighbor_homeobox_Canis_lupus_familiaris (Used in Figure 1C, mammal NANOGNB group)

MRRKLFKVERTPDTLKAPDTSNFAIQKEPAMLCGQNPEQSNRNHHEDERRKEKWRERGEEKEVEVEEKLEEEEEKEERCPQERLVSKPLMDTLWAMFKLNKCPTRGDSQSLAFEFNMTVKQIKQWFRKRRKSYNKDMYKQKYKKRSKSH

>gi_859859290_ref_XP_004766798.2__PREDICTED_NANOG_neighbor_homeobox_Mustela_putorius_furo

MLCDQNPGQAKRNRSEDERKGKKKWKEREREEKEVEIEDGLEEEQEKEEKNEEQYPQKRIVSKPLMDTLWANFKLNKCPTIGDIRSLAFEFNMTVKQIKQWFHKRRKKYNKDMYKQKPKKRPKRRLCVALFLADTGALDPRAGAGTPEDKRQGPQSAVPAHDVQRPAAIAVQLQRTRLLSLRELTVAGYGDTPIS

>gi_961713726_ref_XP_014916992.1__PREDICTED_NANOG_neighbor_homeobox_Acinonyx_jubatus

MSLHQRGLEAGDNFTKSTSWNFAIQKEPDVLCDQNPEQSNRKHSDDERKGKKKWKEREGEEKKEELEEKLEEEQEMEEENEEQHPQERLVSKPLMDTLWATFKLNKCPTMGDSRSLAFEFNMTVKQIKQWFHKRRKKYNKVMYKRKHKKRPKR

>gi_755747243_ref_XP_011282107.1__PREDICTED_NANOG_neighbor_homeobox_Felis_catus

MPNSCEGHRTRNPGDAIGPGVFRRARNLPQGPRDGLSRGLSGSKEVSPQPNPTSACWLFLLRPRGHPDKAFALIGTAGVSQPGSGTSHRLQRAQGGVASPRAGCSAACTLNPCQSEAPVPAQKLSVSASNSPGWLLQEFVVAEACAPCSNFQANHDPRPECGSTGGPVEGDYPTDNPPFIKAKLHKSGSTRSFTVRGPPCSPDPSTPWALPSGWLDSSASGNVLAAMVDVPKTQPTFGKKCGNHQPHKVTKYKEGKDSLCAQGKRRYARKQRGYGGQTKPIFWKKAKTAEKIVLFEWVEPNCRSKRMLAIKSKCFEPQGQSTKSTEGAPRGRGWAGSGSPLPHRNSLSAAEIRDLRDEGKGLFMIESGRVPDSNVSNLVGIFNPDFAIQKEPDVLCDQNPEQSNRNHSDDERKGKKKWNEREGEEKKEELEEKLEEEQEMEEENEEQHPQERLVSKPLMDTLWATFKLNKCPTMGDSRSLAFEFNMTVKQIKQWFHKRRKKYNKVMYKRKHKKRPKRC

>NANOG_1_Lepisosteus_oculatus gi|973216295|ref|XP_015194659.1|:1-446 PREDICTED: homeobox protein Hox-A4-like isoform X1 [Lepisosteus oculatus] (Used in Figure 1C)

meewnslpnyssayhtyaygmtyppppvqnhpslgwsgtgftpsgsdglfldvpptapqnalpqdatrvpgvplagspsdsssetepstpdswsspstdesyalrpppapcspaetacaepapcspaetacaepapcspaeiawaepasssqaegvgeqgallepgpalaagkrshrlrmayserqvmvlcecymikmyltpaemksladkiglsykqvkswfqnrrmrekrlqkkmtsggelvlssvypsiqaqaskgpssvypkispqtseslssvypdispqmltdptcpyssiphqplessssvypnispqvlvnpsccypsipprisespssaypyippqfrgfystsqfgnqgyfkknpppyypsqpcypsrvnvaagsleswalpsagrfkygslngpirysgkvigyecstaplqpqasatlpqwggeelqipasfrk

>NANOG_2_Lepisosteus_oculatus gi|973216279|ref|XP_015194651.1|:1-352 PREDICTED: homeobox protein NANOG-like [Lepisosteus oculatus] (Used in Figure 1C)

Msplfgadsvesgappwppgeldswsspsadesyalrpppapcspaetacaepvpapcswakgaeeqgqqqgalldmdpgaapaagkrarklrtafsetqmtalcerymikmyltpaemksladkiglsykqvktwfqnrrmkqkrqqktltegeeqvpssgypiiqplllessssaypnipplalvstssaytniqaqtsessaypnaqaeasesspypsiqtqsfedlgsvypnfqaqtlesptsvhasvspqtsesptavypnvspqtskssssvypnislqfsvnpncpypniplvtseipsslynnvspnilegpssaypnippqlsfrfpasrpadpradlptllp

>XP_011519152.1 PREDICTED: homeobox protein NANOG isoform X1 [Homo sapiens] (Used in Figure 1C, mammal NANOG group)

MSVDPACPQSLPCFEASDCKESSPMPVICGPEENYPSLQMSSAEMPHTETVSPLPSSMDLLIQDSPDSSTSPKGKQPTSAEKSVAKKEDKVPVKKQKTRTVFSSTQLCVLNDRFQRQKYLSLQQMQELSNILNLSYKQVKTWFQNQRMKSKRWQKNNWPKNSNGVTQASAPTYPSLYSSYHQGCLVNPTGNLPMWSNQTWNNSTWSNQTQNIQSWSNHSWNTQTWCTQSWNNQAWNSPFYNCGEESLQSCMQFQPNSPASDLEAALEAAGEGLNVIQQTTRYFSTPQTMDLFLNYSMNMQPEDV

>nanog_Choloepus_hoffmanni_ENSCHOT00000000973_peptide:ENSCHOP00000000858_pep:NOVEL_protein_coding (Used in Figure 1C, mammal NANOGNB group)

MSVDLASTQSLPCPETDNSRESSPMPEIYEAEKNYASLQMSSAEVHQPETVSPLPSSVDLLIQDSPDSSTSPKVKLPMSGGKSAGRKEDEGQVKKQKIRTVFSQTQLCILNDRFQKQKYLSLQQMQELSNILNLSYKQVKTWFQNQRMKCKKWQKNTNWSKNGNSVIQKGSAPTEFPGLYSSYPQGCLANTSGNFPMWSNQTWNSPIWSSHSWSSQHWYTQAWNNNPVWNNQFQNSGEESLQLQVQFQQNSPASDLEATLGSAGENHNVIEQAAKYFSTLHTMDLFPNYFMNTQPE

>nanognb_Choloepus_hoffmanni_ENSCHOT00000004719peptide:ENSCHOP00000004164pep:KNOWN_BY_PROJECTION_protein_coding (Used in Figure 1C, mammal NANOGNB group)

RQQAMSCNQNPEQSNRKHGKDERRRKKKWKKGAAAGRSRGGAAAEKQQKQKQEEQEEPQQQNKECCQHRERKSVSRPLMDKLWAKFKLNNCPSARQHQSLSLEFGLTDKQISQWFNKMRKKYNKEMSQLKHNIRQQ

>nanog_Procavia_capensis_ENSPCAT00000015420peptide:ENSPCAP00000014417pep:NOVEL_protein_coding (Used in Figure 1C, mammal NANOG group)

SPLPSSMDQLAQDSPDSSTSPKIKPLASASEKSLVKKEGKGQGKKQKVRTVFSQTQLCVLKDRFQKQKYLSLQQMQELSEVLNLSYKQVKTWFQNQRMKCKRWQKNTTWSKNGSGFTQKILTPNKFLELCSSYHQGCLNNQTWNNPVWSNQTWNNSAWSNQTWNTQAWGNHSWNSQAWCTQSWNSQSSWNNQFQSCGEEFLLPQIQFQPNSVSDLETSQALDLFLNYSMNMQPEDV
